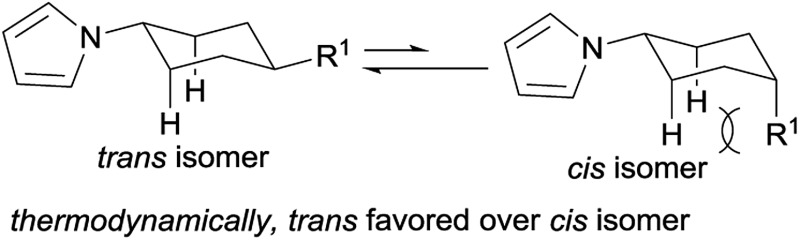# Formal aromaticity transfer for palladium-catalyzed coupling between phenols and pyrrolidines/indolines†
†Electronic supplementary information (ESI) available. See DOI: 10.1039/c7sc02578e



**DOI:** 10.1039/c7sc02578e

**Published:** 2017-08-10

**Authors:** Zihang Qiu, Jiang-Sheng Li, Chao-Jun Li

**Affiliations:** a Department of Chemistry , FQRNT Centre for Green Chemistry and Catalysis , McGill University , 801 Sherbrooke St. W. , Montreal , Quebec H3A 0B8 , Canada . Email: cj.li@mcgill.ca; b School of Chemistry and Biological Engineering , Changsha University of Science & Technology , Changsha 410114 , China

## Abstract

We herein describe a palladium-catalyzed formal aromaticity transfer coupling reaction between phenols and pyrrolidines or indolines to generate the corresponding *N*-cyclohexyl pyrroles or indoles. In this transformation, the aromaticity of phenols is formally passed on to the pyrrolidine or indoline units. Substituted phenols thus can serve as latent cyclohexyl equivalents for the fast construction of various *N*-cyclohexyl pyrroles and indoles.

## Abstract 2

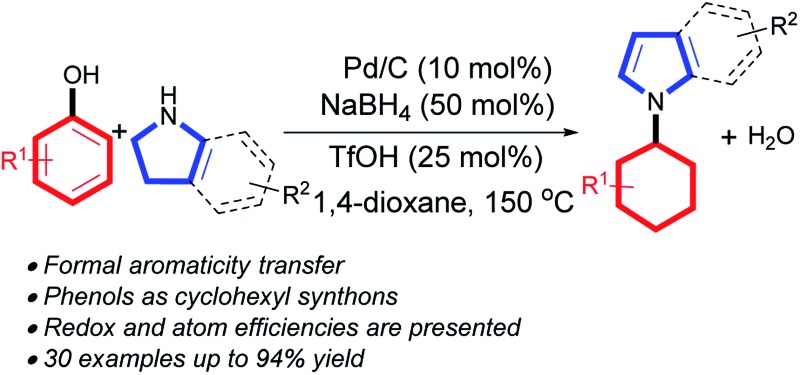
A formal aromaticity transfer reaction between phenols and pyrrolines/indolines has been developed: a redox- and atom-efficient method to synthesize *N*-cyclohexylpyrroles/indoles.

## 

Phenols are widely available and can be obtained at low cost from nature, as they comprise one of the basic units in lignocellulosic biomass as well as coal.^1^ Phenols are therefore ideal aromatic coupling partners and cyclic C-6 feedstocks because of their renewable and sustainable profiles. The past few decades have witnessed tremendous progress in the cross-coupling (C–O bond cleavage) of phenol derivatives, pioneered by Shi,^2^ Chatani,^3^ Martin^4^ and others.^5^ In addition, phenols can potentially be used as cyclohexyl synthons due to their facile reduction, in which cyclohexanones or cyclohexenones can be obtained under controlled hydrogenation conditions.^6^ The combination of their reductive and renewable features has led to increasing interest in the selective reduction and transformation of phenols and cyclohexanones or cyclohexenones (the reduced forms of phenols) in recent years.^7^

Previously, we and others developed a homogeneous palladium-catalyzed oxidative aromatization process to synthesize aromatic amines, utilizing cyclohexanones or cyclohexenones as cyclic aromatic synthons (Scheme 1a).^7g,l^ Recently, a reductive coupling reaction between phenols and amines to form cyclohexylamines succeeded using phenols as cyclohexyl synthons under Pd/C-catalyzed transfer hydrogenation conditions (Scheme 1b),^7*k*^ which was also successful in a flow reactor.^8^ Later, a formal direct coupling of phenols with amines to generate aromatic amines was realized *via* an *in situ* hydrogenation–dehydrogenation (“H-borrowing”) strategy to maintain the aromatic nature of the starting phenol ring overall (Scheme 1c).^7*j*^ Inspired by these early successes, we are intrigued by the possibility of an aromaticity transfer reaction between phenols and pyrrolidines/indolines to afford *N*-cyclohexyl pyrroles/indoles (Scheme 1d). Such a transformation is both atom-^9^ and redox-economical,^10^ as the aromaticity of the phenols will be formally passed on to the pyrrolidine or indoline motifs in this process. Furthermore, the aromaticity transfer products, *N*-cyclohexyl pyrroles/indoles, are valuable units in various bioactive molecules, such as antitumor, antibacterial and antiviral agents.^11^

**Scheme 1:**
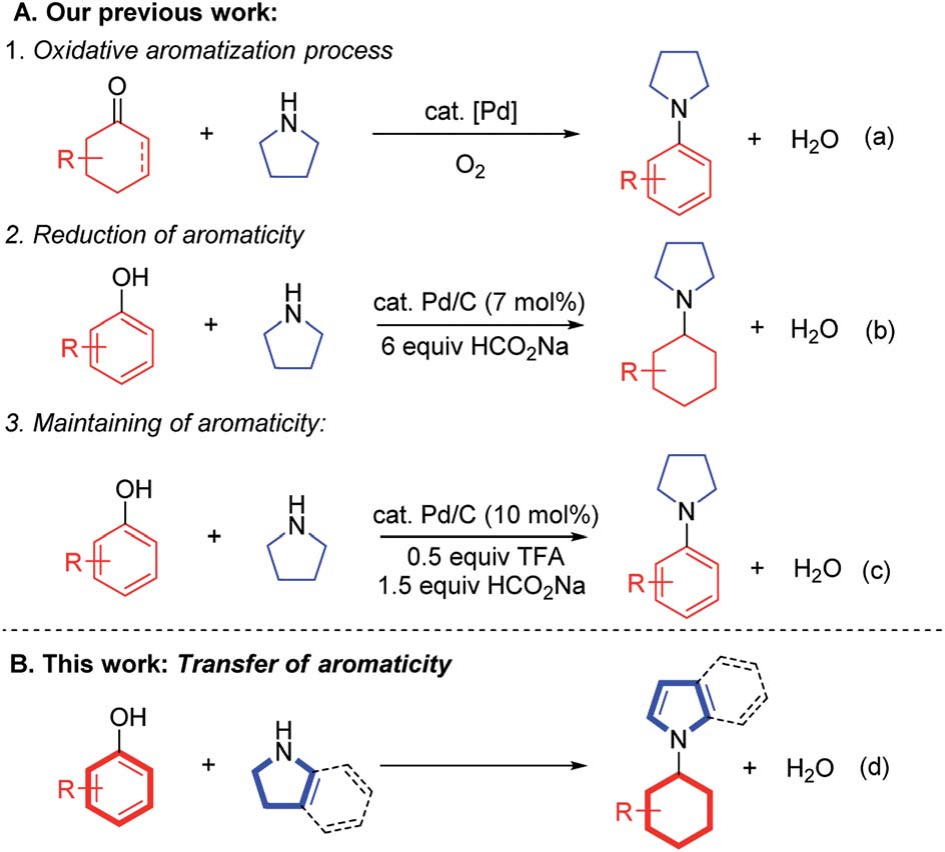
Design of the aromaticity transfer reaction.

To investigate the feasibility of our hypothesis, we initially tested the reaction of phenol with pyrrolidine using Pd/C as the catalyst, 1.5 equiv. HCO_2_Na as the hydride source and toluene as the solvent at 140 °C (Table 1, entry 1). Gratifyingly, the desired aromaticity transfer product, *N*-cyclohexyl pyrrole (**3a**), could be obtained in 9% NMR yield along with two by-products: the direct cross-coupling product, *N*-phenylpyrrole (**4**), as well as the net reduction product, *N*-cyclohexylamine (**5**). A strong solvent effect was observed in this transformation. When 1,4-dioxane was used as the solvent instead of toluene (Table 1, entry 2), the yield of desired product **3a** was significantly increased to 31%. Since acids can promote the condensation of ketones with amines, various acids were evaluated (Table 1, entries 3–5 and Table S1, ESI†). Generally, Brønsted acids worked better than Lewis acids (Table S1†) and among all of the Brønsted acids examined, stronger acids gave better results than weaker acids. Following this trend, TfOH showed the best efficiency, generating the desired product in 69% NMR yield (Table 1, entry 5). Elevating the reaction temperature from 140 °C to 160 °C slightly increased the desired product yield (Table 1, entry 6). Increasing the amount of HCO_2_Na from 1.5 equiv. to 2.0 equiv. decreased the yield slightly; however, when the amount of TfOH was simultaneously increased from 0.5 equiv. to 1.0 equiv., the yield was increased to 77% (Table 1, entries 7 and 8). This might suggest that a balanced combination of acidity and hydride donor amount is essential in this reaction system. Lowering the temperature from 160 °C to 150 °C resulted in a similar reaction efficiency (Table 1, entries 8 and 9). When we tried to use the entry 9 conditions to explore the reaction scope initially, unfortunately, we found that the conversion of phenols was low with phenols bearing bulky substituents, such as 3-*tert*-butyl-phenol. To overcome this problem, NaBH_4_, which is a stronger hydride donor than HCO_2_Na,^12^ was used as the hydride source to re-examine the reaction system (Table 1, entries 10–12 and Table S2†). Ultimately, by carefully selecting the combination and amount of acid and NaBH_4_ (Table S2†), the desired product could be obtained in 80% yield (Table 1, entry 12).

**Table 1:** Optimization of the reaction conditions^*a*^

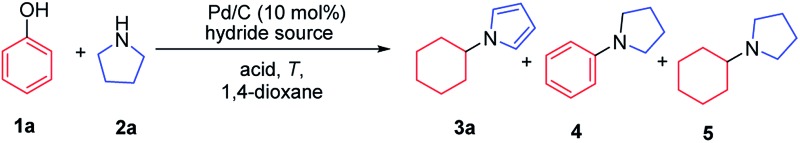						
Entry	Hydride source (mol%)	Acid (mol%)	*T* (°C)	Yield (%)		
				**3a**	**4**	**5**
1^*b*^	HCO_2_Na (150)	—	140	9	27	32
2	HCO_2_Na (150)	—	140	31	41	14
3	HCO_2_Na (150)	PhCO_2_H (50)	140	23	43	6
4	HCO_2_Na (150)	TFA (50)	140	56	20	13
5	HCO_2_Na (150)	TfOH (50)	140	69	8	10
6	HCO_2_Na (150)	TfOH (50)	160	71	13	2
7	HCO_2_Na (200)	TfOH (50)	160	61	20	9
8	HCO_2_Na (200)	TfOH (100)	160	77	7	7
9	HCO_2_Na (200)	TfOH (100)	150	75	5	11
10	NaBH_4_ (50)	TfOH (100)	150	40	20	18
11	NaBH_4_ (50)	TfOH (50)	150	71	5	10
**12**	**NaBH**_**4**_ **(50)**	**TfOH (25)**	**150**	**80 (80)**	**8**	**4**

^*a*^Reaction conditions: phenol (0.2 mmol, 1 equiv.), pyrrolidine (0.28 mmol, 1.4 equiv.), 10 mol% of 5 wt% Pd/C and the chosen acid and hydride source were stirred in 1,4-dixoane (1 mL) under argon in a 10 mL sealed tube for 12 h. NMR yields are given with 1,3,5-trimethoxylbenzene as the internal standard, with the isolated yield given in parentheses.

^*b*^Toluene was used as the solvent. For details of the optimization, please see the ESI. TFA = trifluoroacetic acid; TfOH = trifluoromethanesulfonic acid.

With the optimized conditions in hand, the substrate scope of phenols was explored next. As shown in Table 2, various alkyl substituted phenols reacted smoothly to afford the corresponding products in moderate to excellent yields (Table 2, **3b–3i**). Bulky alkyl substituted phenols such as 3- or 4-*tert*-butyl phenols, which are relatively more difficult to reduce due to their steric hindrance, reacted well to give 63% and 79% yields, respectively. The *cis*/*trans* ratio followed the trend of the steric size of the alkyl substituents on the phenols. For example, as shown in **3d**, **3f** and **3h**, the *cis*/*trans* ratio changed from 1/4.9 to 1/16 with the increase in substituent size, suggesting a thermodynamically controlled process in which the *trans*-isomer is more favourable with the increasing size of the 4-alkyl substituent in the cyclic six-membered ring conformation.^16^ Phenols bearing both electron-withdrawing and electron-donating substituents all worked well to give the corresponding products in moderate to excellent yields, with electron-donating substituents giving a higher yield than electron-withdrawing ones (**3m** *vs.* **3l**). As expected, both *ortho*- and *para*-methoxy phenols afforded the desired products smoothly; however, with the *meta*-methoxy phenol, the Ar–OMe bond was completely cleaved during the reaction to give *N*-cyclohexylpyrrole (**3a**) in 43% yield, likely due to the facile β-elimination of the 3-methoxycyclohexanone intermediate.^7*k*^ When 4-fluorophenol was used as the substrate, the defluorination product was obtained, which is consistent with our previous report.^7*j*^ It is worth mentioning that the trifluoromethyl group (**3n**), an extra phenolic OH (**3p**) and a sterically bulky amide (**3q**) were all tolerated in this transformation. Moreover, bioactive phenol derivatives, such as tyramine and tyrosine, were suitable substrates for this transformation (**3r** and **3u**). In addition, di-substituted phenols also reacted efficiently to give the desired products in moderate yields (**3s** and **3t**). Importantly, carvacrol, a natural product from the essential oil of *Origanum vulgare*, underwent this transformation as well (**3t**).

**Table 2:** Substrate scope of phenols^*a*^

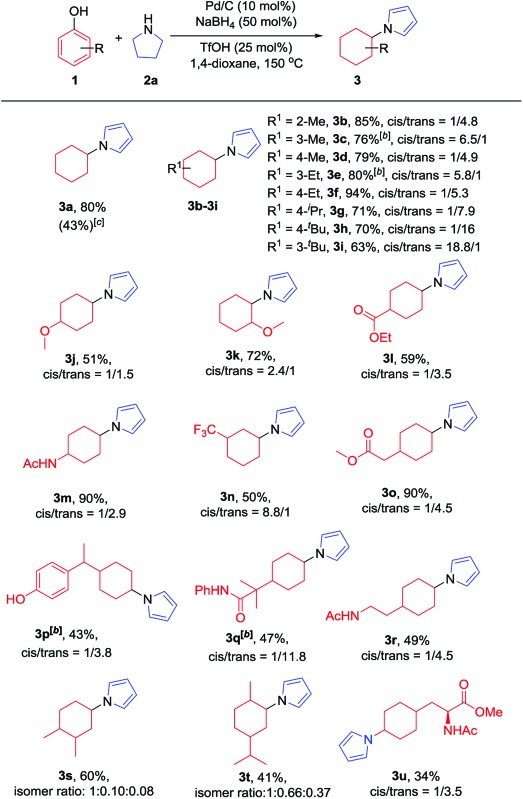

^*a*^Reaction conditions: phenols (0.2 mmol, 1 equiv.), pyrrolidine (0.28 mmol, 1.4 equiv.), Pd/C (10 mol%), NaBH_4_ (50 mol%) and TfOH (25 mol%) were stirred in 1,4-dioxane (1 mL) at 150 °C under argon in a 10 mL sealed tube for 12 h. Isolated yields are given unless otherwise noted. The *cis*/*trans* (isomer) ratio was determined by crude ^1^H NMR.

^*b*^62.5% NaBH_4_ was used.

^*c*^3-Methoxyphenol was used as the substrate and the NMR yield is given with 1,3,5-trimethoxybenzene as the internal standard.

Additionally, the substrate scope of pyrrolines/indolines was investigated, as summarized in Table 3. Indoline worked well to give the corresponding *N*-cyclohexylindole (**6b**) in 90% isolated yield. The alkyl-substituted pyrroline and indolines, such as 2-methylpyrrolidine and 2- or 3-methylindoline, were all effective substrates, affording the corresponding pyrrole and indole derivatives in good to excellent yields (**6a**, **6c** and **6d**), while 7-methylindoline gave the corresponding product in a relatively lower yield, probably due to the steric hindrance of the 7-Me group (**6g**). Interestingly, when proline or indoline-2-carboxylic acid were used as substrates, the corresponding decarboxylation products were generated in moderate yields;^7l,13^ however, the corresponding methyl esters of proline and indoline-2-carboxylic acid failed to give the desired products. Indoline-3-carboxylic methyl ester gave a low yield, while indoline-3-ethyl acetate could afford the product in moderate yield (**6f** and **6e**). The indoline with an electron-donating substituent worked better than that with an electron-withdrawing one (**6h** and **6i**), in line with the nucleophilicities of the indoline derivatives.

**Table 3:** Substrate scope of pyrrolidines/indolines^*a*^

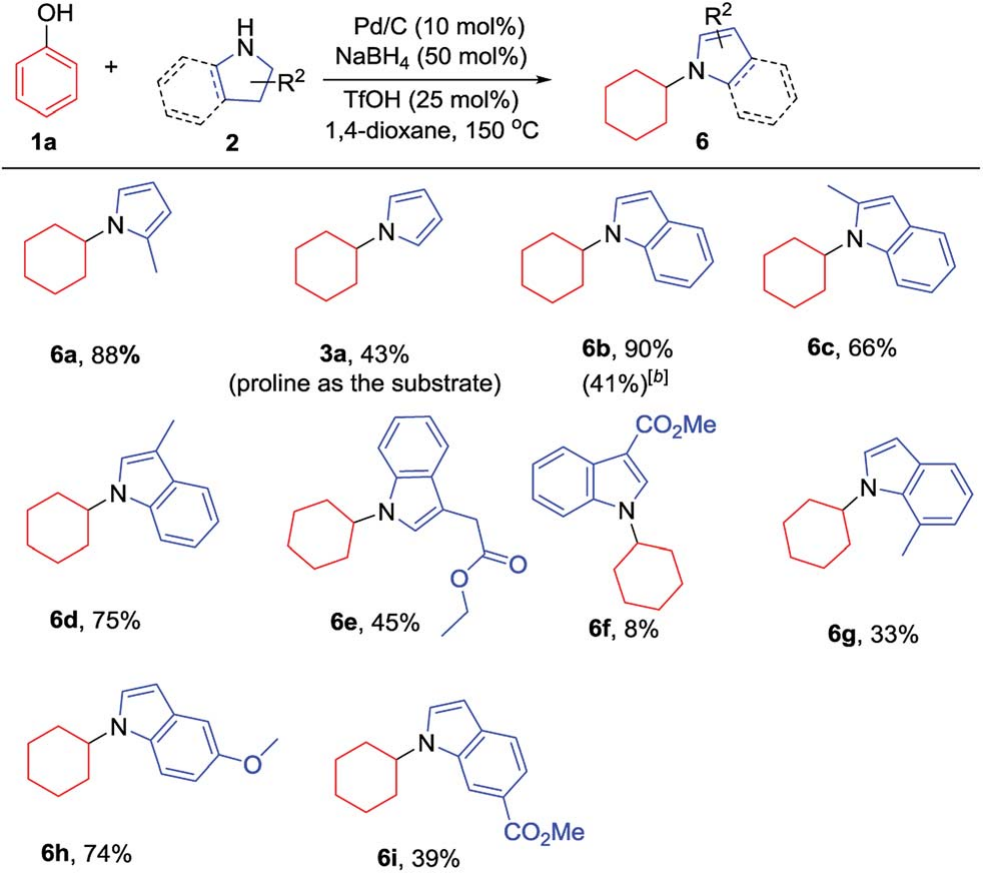

^*a*^Reaction conditions: phenol (0.2 mmol, 1 equiv.), pyrrolidines (0.28 mmol, 1.4 equiv.) or indolines (0.48 mmol, 2.4 equiv.), Pd/C (10 mol%) and TfOH (25 mol%) were stirred in 1,4-dioxane (1 mL) at 150 °C under argon in a 10 mL sealed tube for 12 h. Isolated yields are given.

^*b*^Indoline-2-carboxylic acid was used as the substrate.

To investigate the possible mechanism, cyclohexanone and cyclohexenone (the two possible reduced forms of phenol) were used (Scheme 2a and b). Both compounds could afford the desired product in 62% and 47% NMR yields, respectively. To further understand the mechanism, the kinetics profile of this transformation was studied, as shown in Fig. 1 (see ESI for details†). The yield of the desired product, **1a**, increased as the reaction proceeded. Interestingly, the two by-products, *N*-phenylpyrrolidine (**4**) and *N*-cyclohexylpyrrolidine (**5**), were formed in reasonable amounts at the onset of this transformation, and they decreased to relatively small amounts at the end of the reaction. This observation indicated that the two redox isomers, *N*-phenylpyrroline (**4**) and *N*-cyclohexylpyrrolidine (**5**), could be potentially converted into the desired product, *N*-cyclohexylpyrrole (**3a**). To examine this possibility, control experiments were performed (Scheme 2c and d). As expected, both **4** and **5** could be converted into **3a** in 48% and 55% NMR yields, respectively.

**Scheme 2:**
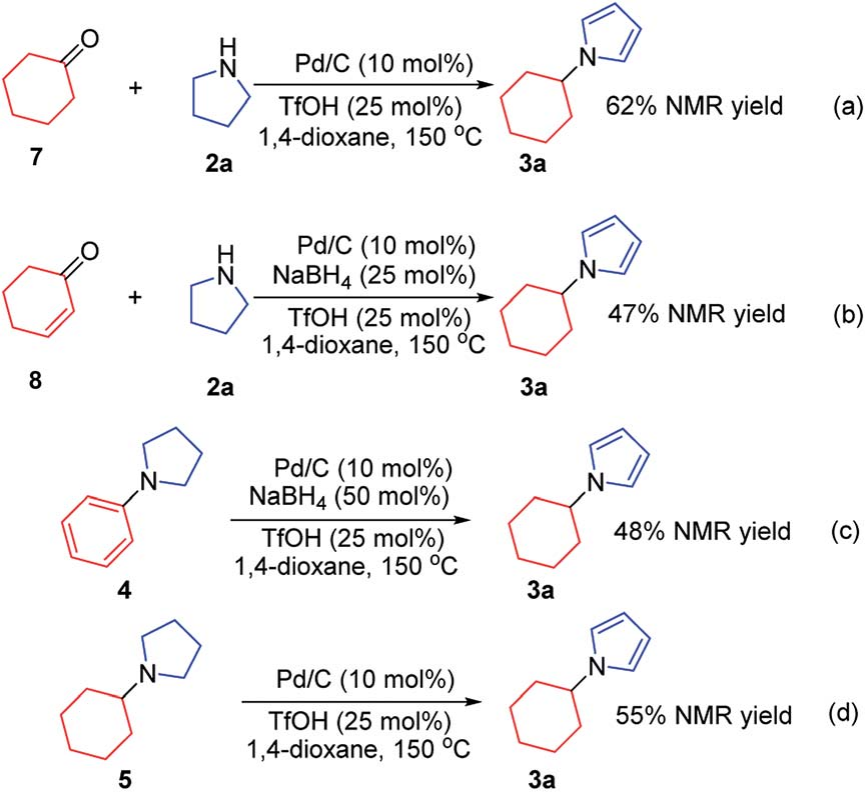
Control experiments.

**Fig. 1:**
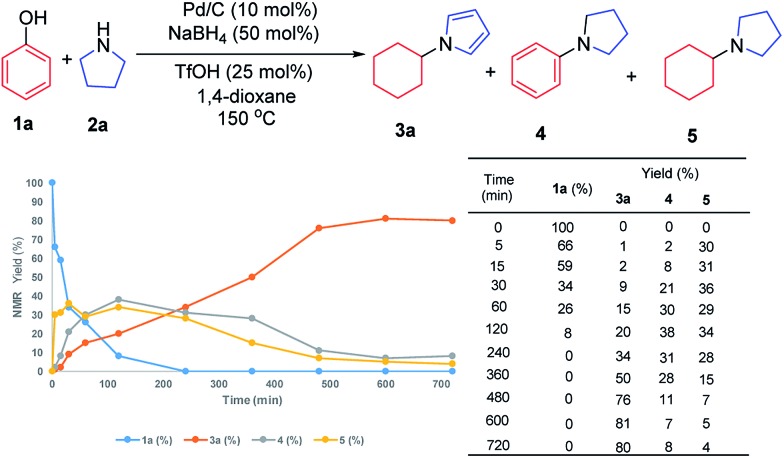
Kinetics profile.

Based on the above results, a tentative mechanism for this formal aromaticity transfer reaction is proposed in Scheme 3. This reaction could be initiated by NaBH_4_ reacting with the palladium catalyst to generate the HPd^II^H species,^7j,15^ which would reduce phenol (**1a**) to form the cyclohexanone or cyclohexenone (**I**). Then, intermediate **I** could undergo fast condensation with the pyrroline (**2a**), catalyzed by TfOH, to give the key intermediate **II**. If intermediate **II** underwent 1,3-hydride transfer to give intermediate **III**,^14^ intermediate **IV** could be generated after deprotonation. Next, the desired product (**3a**) could be obtained by dehydrogenation of intermediate **IV**, catalyzed by palladium, to regenerate the HPd^II^H species,^7j,15^ as stated in **Pathway A**. Alternatively, based on our previous work (Scheme 1b and c),^7k,7j^ intermediate **II** could also be transformed into intermediates **4** or **5**. Based on the results of the control experiments (Scheme 2), intermediates **4** and **5** could both afford the desired product **3a**, by either a hydrogenation process (**Pathway B**) or a dehydrogenation process (**Pathway C**), catalyzed by palladium.

**Scheme 3:**
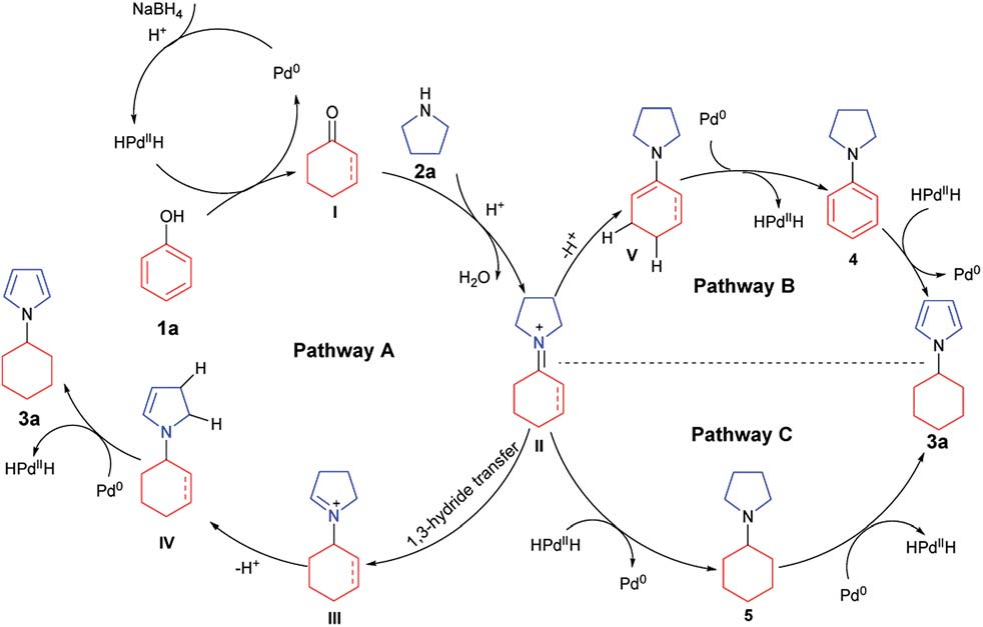
Tentative mechanism.

## Conclusions

In conclusion, we have developed a formal aromaticity transfer reaction between phenols and pyrrolines/indolines that is both atom- and redox-economical. A wide range of *N*-cyclohexylpyrroles and *N*-cyclohexylindoles bearing various functional groups were obtained by this novel method, starting from naturally abundant and sustainable phenols. Several bioactive phenols, such as tyramine, tyrosine and carvacrol, are all suitable substrates in this transformation. Ongoing studies regarding the synthetic applications of such transformations are currently underway in our laboratory.

## Conflicts of interest

There are no conflicts to declare.
