# Supplementary material for: Formal aromaticity transfer for palladium-catalyzed coupling between phenols and pyrrolidines/indolines
Source: Chem Sci. 2017 Aug 10;8(10):6954–8. doi: 10.1039/c7sc02578e (PMC5642148; doi:10.1039/c7sc02578e)
Supplement: Supplementary file 1 [file SC-008-C7SC02578E-s001.pdf]

Electronic Supplementary Material (ESI) for Chemical Science.
This journal is © The Royal Society of Chemistry 2017

Supporting Information

Formal Aromaticity-Transfer for Palladium-Catalyzed Coupling between Phenols and Pyrrolidines/Indolines

Zihang Qiu[a], Jiang-Sheng Li[ab] and Chao-Jun Li*[a]

Table of Contents:

I. GENERAL EXPERIMENTAL INFORMATION 3
II. EXPERIMENTAL PROCEDURE 3
III. SELECTED OPTIMIZATION OF THE REACTION CONDITIONS 4
IV. PROCEDURES FOR KINETICS EXPERIMENTS 5
V. GENERAL PROCEDURES TO SYNTHESIZE INDOLINES 6
VI. SPECTROSCOPIC DATA OF PRODUCTS 7
VII. REFERENCES 26
VIII. NMR SPECTRA OF PRODUCTS 27

I. General Experimental Information

All reactions were carried out in flame-dried 10 mL U-shaped biotage microwave reaction tubes, covered by aluminum seals with PTFE-faced silicone septa, under an atmosphere of argon, unless otherwise stated. All reported reaction temperatures correspond to oil bath temperatures. Solvents and reagents were purchased from Sigma-Aldrich chemical company and Fisher Scientific, and were used without further purification unless otherwise specified. 1,4-Dioxane and toluene were purified by the *Pure Solvent MD-7* purification system (Innovative Technology). Pentane and pyrrolidine were distilled under atmosphere pressure prior to use. Product purifications were preformed either with preparative chromatography on a Biotage Isolera One automated chromatography system with neutral aluminum oxide (activated, ~150 mesh) or with preparative analytical thin-layer chromatography (TLC) using E. Merck silica gel 60 F_{254} pre-coated plates (0.25 mm).

NMR Spectroscopy: Nuclear magnetic resonance (^{1}H, ^{13}C, ^{19}F) spectra were recorded on a Bruker AV500 equipped with a 60-position Sample Xpress sample changer (^{1}H, 500 MHz; ^{13}C, 125 MHz, ^{19}F 471 MHz). Chemical shifts are expressed in parts per million (ppm) units downfield from TMS, with the solvent residue peak as the chemical shift standard ($CDCl_3$: δ 7.26 ppm in ^{1}H NMR; δ 77.16 ppm in ^{13}C NMR, CD_3OD: δ 3.31 ppm in ^{1}H NMR; δ 49.00 ppm in ^{13}C NMR). Data are reported as following: chemical shift, multiplicity (s = singlet, d = doublet, dd = doublet of doublets, t = triplet, td = triplet of doublets, q = quartet, quint = quintet, sext = sextet, sep = septet, m = multiplet, br = broad singlet), coupling constants *J* (Hz), and integration.

Mass Spectrometry: Mass spectrometry (MS) was performed by the McGill Chemistry Department Mass Spectrometry Facility. High Resolution Mass spectra were recorded using electrospray ionization (ESI+) and/or atmospheric pressure chemical ionization APCI(+/-), performed either on "Exactive Plus Orbitrap" a ThermoScientific high resolution accurate mass (HR/AM) FT mass spectrometer, or a Bruker Daltonics Maxis Impact quadrupole-time of flight (QTOF) mass spectrometer. Protonated molecular ions $(M+H)^+$ or sodium adducts $(M+Na)^+$, were used for empirical formula confirmation.

II. Experimental Procedure

General procedures: Pd/C (5 wt%,10 mol% based on Pd content) was added into a flame-dried biotage 10 mL U-shape microwave tube charged with a magnetic stir bar. The tube was then vacuumed, stirred and heated in oil-bath at 140 °C for 1 h to pre-activate the Pd/C. Next, phenols (0.2 mmol, 1 equiv) and $NaBH_4$ (0.1 mmol, 0.5 equiv) were added under argon protection. After three cycles of evacuation/backfilling sequence with argon, 1,4-dioxane (1.0 mL), pyrrolines (0.28 mmol, 1.4 equiv) or indolines (0.48 mmol, 2.4 equiv) and TfOH (25 mol%) were added. Then the tube was sealed and the mixture was stirred at 150 °C in the pre-heated oil bath at 750 rpm for 12 h. After completion, the reaction mixture was diluted with pentane or EtOAc and filtered through a pad of celite. The filtrate was then concentrated *in vacuo*, and the resulting residue was purified by

column chromatography on neutral alumina or preparative TLC to afford the corresponding products.

III. Selected Optimization of the Reaction Conditions

Table S1. Evaluation of acids[a]

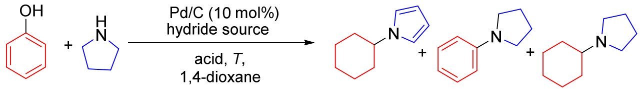

1a **2a** **3a** **4** **5**

Entry	Hydride source (x mol%)	Acid (y mol%)	T (°C)	Yield (%)		
				**3a**	**4**	**5**
1	HCO_2Na (150)	$PhCO_2H$ (50)	140	23	43	6
2	HCO_2Na (150)	H_3PO_4 (50)	140	53	10	16
3	HCO_2Na (150)	CSA (50)	140	23	48	19
4	HCO_2Na (150)	TFA (50)	140	56	20	13
5	HCO_2Na (150)	$HBF_4 \cdot OEt_2$ (50)	140	60	10	14
6	HCO_2Na (150)	TfOH (50)	140	69	8	10
7	HCO_2Na (150)	$Sc(OTf)_3$ (50)	140	45	22	14

[a] Reaction conditions: phenol (0.2 mmol, 1 equiv), pyrrolidine (0.28 mmol, 1.4 equiv), 10 mol% of 5 wt% Pd/C, acid with hydride source in 1,4-dixoane (1 mL) were stirred under argon in a 10-mL sealed tube for 12 h; NMR yields were given with 1,3,5-trimethoxylbenzene as the internal standard; TFA = trifluoroacetic acid; TfOH = trifluoromethanesulfonic acid; CSA: camphorsulfonic acid.

Table S2. $NaBH_4$ was used as the hydride source to re-examine the reaction system[a]

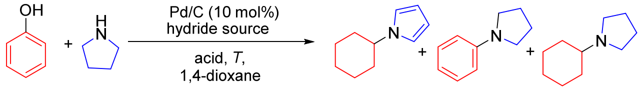

1a **2a** **3a** **4** **5**

Entry	Hydride source (x mol%)	Acid (y mol%)	T (°C)	Yield (%)		
				**3a**	**4**	**5**
1	$NaBH_4$ (25)	TfOH (50)	140	44	13	8
2	$NaBH_4$ (37.5)	TfOH (50)	140	66	9	5
3	$NaBH_4$ (50)	TfOH (50)	140	65	6	6
4	$NaBH_4$ (50)	TfOH (100)	150	40	20	18
5	$NaBH_4$ (50)	TfOH (50)	150	71	5	10
6	$NaBH_4$ (50)	TfOH (37.5)	150	76	5	5
**7**	**$NaBH_4$ (50)**	**TfOH (25)**	**150**	**80**	**8**	**4**
8	$NaBH_4$ (50)	TFA (25)	150	78	4	8
9	$NaBH_4$ (50)	TfOH (17.5)	150	50	20	19
10	$NaBH_4$ (50)	TfOH (10)	150	41	26	28

[a] Reaction conditions: phenol (0.2 mmol, 1 equiv), pyrrolidine (0.28 mmol, 1.4 equiv), 10 mol% of 5 wt% Pd/C, acid with hydride source in 1,4-dixoane (1 mL) were stirred under argon in a 10-mL sealed tube for 12 h; NMR yields were given with 1,3,5-trimethoxylbenzene as the internal standard; TfOH = trifluoromethanesulfonic acid; TFA = trifluoroacetic acid.

Table S3. Effect of pyrrolidine amount[a]

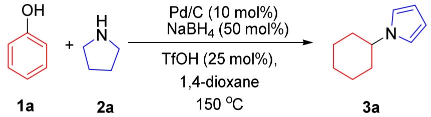

1a **2a** **3a**

Entry	Amount of 2a (x equiv)	Yield (%) **3a**
1	1.0	55
2	1.2	66
**3**	**1.4**	**80**
4	1.6	76

[a] Reaction conditions: phenol (0.2 mmol, 1 equiv), pyrrolidine (x equiv), 10 mol% of 5 wt% Pd/C, TfOH (25 mol%) with $NaBH_4$ (50 mol%) in 1,4-dixoane (1 mL) were stirred under argon in a 10-mL sealed tube for 12 h; NMR yields were given with 1,3,5-trimethoxylbenzene as the internal standard; TfOH = trifluoromethanesulfonic acid.

Table S4. Effect of indoline amount[a]

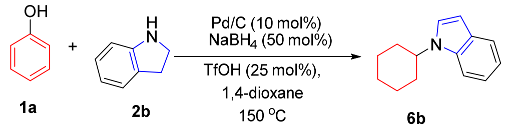

1a 2b 6b

Entry	Amount of 2b (x equiv)	Yield (%) **6b**
1	1.4	28
2	1.6	40
3	1.9	57
4	2.2	72
**5**	**2.4**	**90**
6	2.6	80

[a] Reaction conditions: phenol (0.2 mmol, 1 equiv), indoline (x equiv), 10 mol% of 5 wt% Pd/C, TfOH (25 mol%) with $NaBH_4$ (50 mol%) in 1,4-dixoane (1 mL) were stirred under argon in a 10-mL sealed tube for 12 h; NMR yields were given with 1,3,5-trimethoxylbenzene as the internal standard; TfOH = trifluoromethanesulfonic acid.

IV. Procedures for Kinetics Experiments

Parallel reactions were set up following the general procedures as stated in Section II, the reactions were stopped and put in the cold water at 5, 15, 30, 60, 120, 240, 360, 480, 600 and 720 mins, respectively. After cooling down the reactions, the mixtures were diluted with pentane and filtered through a pad of celite. The filtrate was then concentrated *in vacuo* and the internal standard (1,3,5-trimethoxylbenzene, 11.2 mg) as well as 0.8 mL $CDCl_3$ were added to run the ^{1}H-NMR. The NMR yields were collected in Table 3 and the kinetics profile was demonstrated in Figure S1.

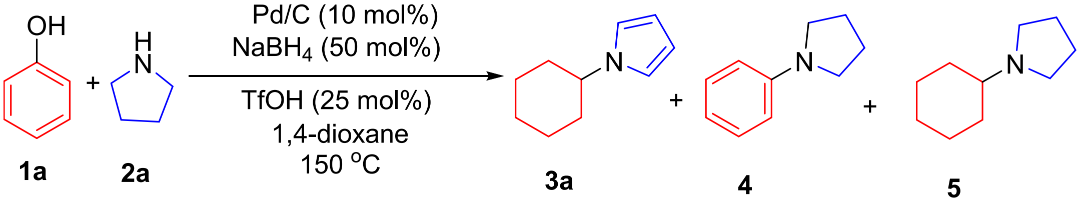

1a 2a 3a 4 5

Table 3. NMR yields data

Time (min)	**1a** (%)	**3a** (%)	**4** (%)	**5** (%)
0	100	0	0	0
5	66	1	2	30
15	59	2	8	31
30	34	9	21	36
60	26	15	30	29

120	8	20	38	34
240	0	34	31	28
360	0	50	28	15
480	0	76	11	7
600	0	81	7	5
720	0	80	8	4

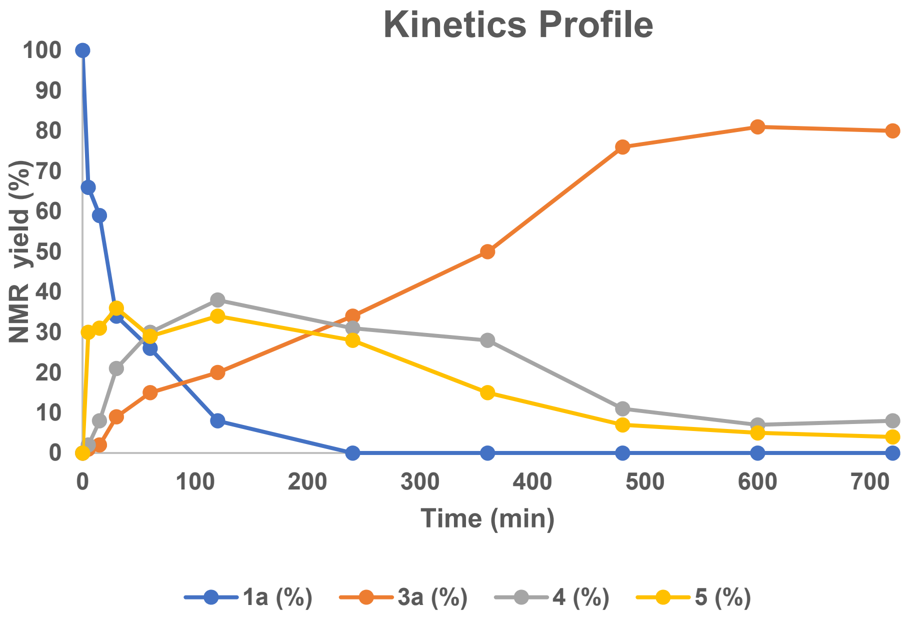

Figure S1. Kinetics profile.

V. General Procedures to Synthesize Indolines

(A) General procedures:

(a) For indolines' synthesis (except methyl indoline-3-carboxylate):

The method was according to the reported literature with minor modifications:[1]

To a solution of the corresponding indole (2 mmol, 1 equiv) in AcOH (10 mL) was added $NaBH_3CN$ (12 mmol, 6 equiv) slowly at 0 °C. The resulting mixture could be raised to room temperature slowly. After no starting material could be detected by TLC analysis (typically 4h), 25 mL H_2O and NaOH pellets were added until pH>12, extracting the solution with Et_2O or EtOAc (3x25 mL). The organic phases were combined, dried over Na_2SO_4 and the solvent was evaporated under reduced pressure. The resulting indolines were purified by flash chromatography.

(b) Method to synthesize methyl indoline-3-carboxylate:

The method was according to the reported literature with minor modifications:[2]

1-(*tert*-Butyl) 3-methyl 1*H*-indole-1,3-dicarboxylate: Methyl 1*H*-indole-3-carboxylate (10 mmol, 1 equiv) was dissolved in THF (50 mL) and cooled to 0 °C, before NaH (13 mmol, 1.3 equiv) was added. After effervescence had ceased, Boc_2O (13 mmol, 1 equiv) was added in one portion, under vigorous stirring, whereupon a precipitate was formed. The mixture was allowed to warm to room temperature and stirred overnight, then extracted with CH_2Cl_2 (3x25 mL) against saturated NH_4Cl solution (100 mL). The combined organic layers were dried over Na_2SO_4, filtered and concentrated under reduced pressure. The residue was purified by flash chromatography (hexanes:EtOAc = 10:1) to afford 1-(*tert*-butyl) 3-methyl 1*H*-indole-1,3-dicarboxylate as a colorless powder in 91% yield.

1-(*tert*-Butyl) 3-methyl indoline-1,3-dicarboxylate: To a solution of 1-(*tert*-butyl) 3-methyl 1*H*-indole-1,3-dicarboxylate (5 mmol, 1 equiv) in MeOH (40 mL) was added Mg turnings (15.5 mmol, 3.1 equiv) at 0 °C and gas evolution was observed afterwards (ca 10 mins). The mixture was stirred at 0 °C for 7 h (if not completed, 2 equiv additional Mg turnings were added). After no starting material could be detected by TLC, saturated NH_4Cl solution was added. The mixture was extracted with CH_2Cl_2 (3x25 mL) and EtOAc (1x50 mL) and the combined organic layer was washed with brine, dried over Na_2SO_4, and concentrated *in vacuo*. The residue was purified by flash chromatography (hexanes:EtOAc = 50:1 to 20:1) to afford the corresponding 1-(*tert*-butyl) 3-methyl indoline-1,3-dicarboxylate as colorless oil in 50% yield.

Methyl indoline 3-carboxylate: Trifluoroacetic acid (2.9 mL) was added to a stirred solution of 1-(*tert*-butyl) 3-methyl indoline-1,3-dicarboxylate (2.5 mmol) in dry CH_2Cl_2 (13 mL), under vigorous stirring at room temperature. The reaction was stirred until no starting material could be detected by TLC (1 h) and was then neutralized by the addition of small portions of aqueous saturated $NaHCO_3$ solution at 0 °C. The mixture was extracted with CH_2Cl_2 (3x20 mL) and the combined organic layers were dried over Na_2SO_4. After filtration and concentration under reduced pressure, the residue was purified by flash chromatography (hexanes:EtOAc = 4:1) to give methyl indoline 3-carboxylate in 76% yield as yellow oil.

VI. Spectroscopic Data of Products

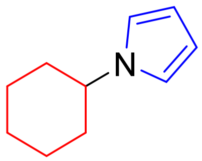

1-Cyclohexyl-1*H*-pyrrole (3a):[3]

Following the general procedure, pentane was used to work up. After column chromatography on neutral alumina (eluent: pentane), **3a** was isolated as a colorless oil (24.0 mg, 80% yield).

^{1}H NMR: (500 MHz, $CDCl_3$, ppm): δ 6.75 (t, *J* = 2.1 Hz, 2H), 6.16 (t, *J* = 2.1 Hz, 2H), 3.83 (tt, *J* = 2.1 Hz, 1H), 2.14 – 2.11 (m, 2H), 1.92 – 1.88 (m, 2H), 1.77 – 1.73 (m, 1H), 1.65 (qd, *J* = 12.5, 3.3 Hz, 2H), 1.42 (qt, *J* = 13.1, 6.7 Hz, 2H), 1.25 (qt, *J* = 12.9, 3.7 Hz, 1H).

^{13}C NMR: (125 MHz, $CDCl_3$, ppm): δ 118.5, 107.4, 58.8, 34.8, 25.9, 25.6

HRMS: (APCI, *m/z*) calcd for $C_{10}H_{16}N$ $[M+H]^+$ 150.1277, found: 150.1276

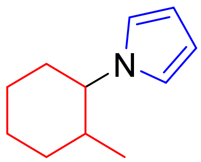

1-(2-Methylcyclohexyl)-1*H*-pyrrole (3b)

Following the general procedure, pentane was used to work up. After column chromatography on neutral alumina (eluent: pentane), **3b** was isolated as a colorless oil (27.7 mg, 85% yield). The major isomer (trans isomer) can be separated as pure compound, the spectroscopic data are as follows:

^{1}H NMR: (500 MHz, $CDCl_3$, ppm): δ 6.67 (t, *J* = 2.1 Hz, 2H), 6.13 (t, *J* = 2.1 Hz, 2H), 3.35 (td, *J* = 3.8 Hz, 1H), 2.06 – 2.01 (m, 1H), 1.89 – 1.83 (m, 2H), 1.76 – 1.64 (m, 3H), 1.42 – 1.29 (m, 2H), 1.14 – 1.06 (m, 1H), 0.68 (d, *J* = 6.5 Hz, 3H).

^{13}C NMR: (125 MHz, $CDCl_3$, ppm): δ 119.0, 107.3, 65.7, 39.5, 35.0, 34.9, 26.3, 26.0, 19.1

HRMS: (ESI, *m/z*) calcd for $C_{11}H_{18}N$ $[M+H]^+$ 164.1434, found: 164.1436

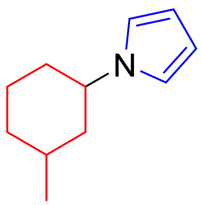

1-(3-Methylcyclohexyl)-1*H*-pyrrole (3c):

Following the general procedure, pentane was used to work up. After column chromatography on neutral alumina (eluent: pentane), **3c** was isolated as a colorless oil (24.8 mg, 76% yield). The cis/trans isomers cannot be separated, the spectroscopic data are as follows:

^{1}H NMR: (500 MHz, $CDCl_3$, ppm): δ 6.77 – 6.73 (t, *J* = 2.1 Hz, 2H), 6.15 – 6.16 (t, *J* = 2.1 Hz, 2H), 4.15 – 3.82 (trans isomer: δ 4.15 – 4.10 (m, 0.14H), cis isomer: δ 3.85 (tt, *J* = 12.0, 7.6 Hz, 0.90H), total 1H), 2.10 – 1.99 (m, 2H), 1.90 – 1.87 (m, 1H), 1.79 – 1.72 (m, 1H), 1.64 – 1.52 (m, 2H), 1.47 – 1.38 (m, 1H), 1.36 – 1.28 (m, 1H), 1.05 – 0.97 (m, 4H).

^{13}C NMR: (125 MHz, $CDCl_3$, ppm): δ 118.5, 107.5, 58.7, 43.3, 34.3, 34.2, 32.5, 25.3, 22.5

HRMS: (ESI, *m/z*) calcd for $C_{11}H_{18}N$ $[M+H]^+$ 164.1435, found: 164.1435

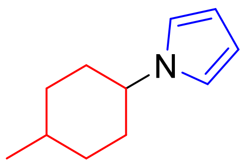

1-(4-Methylcyclohexyl)-1*H*-pyrrole (3d):

Following the general procedure, pentane was used to work up. After column chromatography on neutral alumina (eluent: pentane), **3d** was isolated as a colorless oil (25.8 mg, 79% yield). The cis/trans isomers cannot be separated, the spectroscopic data are as follows:

^{1}H NMR: (500 MHz, $CDCl_3$, ppm): δ 6.79 – 6.74 (t, *J* = 2.1 Hz, 2H), 6.17 – 6.15 (t, *J* = 2.1 Hz, 2H), 3.92 – 3.77 (cis isomer: δ 3.92 – 3.87 (m, 0.17H), trans isomer: δ 3.80 (tt, *J* = 12.0, 3.9 Hz, 0.90H), total 1H), 2.12 – 1.96 (m, 2H), 1.89 – 1.83 (m, 2H), 1.73 – 1.63 (m, 2H), 1.55 – 1.42 (m, 1H), 1.16 – 1.08 (m, 2H), 1.02 – 0.95 (cis isomer: δ 1.02 (d, *J* = 7.05, 0.5H), trans isomer: δ 0.96 (d, *J* = 6.6, 2.7H), total 3H).

^{13}C NMR: (125 MHz, $CDCl_3$, ppm): δ 118.6, 107.5, 58.7, 34.5, 34.4, 32.1, 22.3

HRMS: (APCI, *m/z*) calcd for $C_{11}H_{18}N$ $[M+H]^+$ 164.1434, found: 164.1435

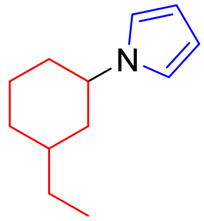

1-(3-Ethylcyclohexyl)-1*H*-pyrrole (3e):

Following the general procedure, pentane was used to work up. After column chromatography on neutral alumina (eluent: pentane), **3e** was isolated as a colorless oil (28.2 mg, 80% yield). The cis/trans isomers cannot be separated, the spectroscopic data are as follows:

^{1}H NMR: (500 MHz, $CDCl_3$, ppm): δ 6.77 – 6.73 (t, *J* = 2.1 Hz, 2H), 6.16 – 6.14 (t, *J* = 2.1 Hz, 2H), 4.09 – 3.81 (trans isomer: δ 4.09 – 4.04 (m, 0.12H), cis isomer: δ 3.84 (tt, *J* = 11.8, 7.6 Hz, 0.92H), total 1H), 2.12 – 2.08 (m, 2H), 2.0 – 1.86 (m, 1H), 1.81 – 1.77 (m, 1H), 1.61 – 1.53 (m, 1H), 1.45 – 1.24 (m, 5H), 0.93 – 0.89 (m, 4H).

^{13}C NMR: (125 MHz, $CDCl_3$, ppm): δ 118.6, 107.5, 58.8, 41.1, 39.2, 34.7, 32.0, 29.8, 25.3, 11.5

HRMS: (APCI, *m/z*) calcd for $C_{12}H_{20}N$ $[M+H]^+$ 178.1590, found: 178.1589

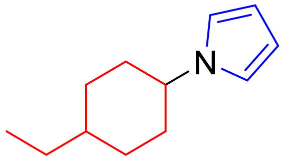

1-(4-Ethylcyclohexyl)-1*H*-pyrrole (3f):

Following the general procedure, pentane was used to work up. After column chromatography on neutral alumina (eluent: pentane), **3f** was isolated as a colorless oil (33.2 mg, 94% yield). The cis/trans isomers cannot be separated, the spectroscopic data are as follows:

^{1}H NMR: (500 MHz, $CDCl_3$, ppm): δ 6.78 – 6.74 (t, *J* = 2.1 Hz, 2H), 6.17 – 6.15 (t, *J* = 2.1 Hz, 2H), 3.94 – 3.78 (cis isomer: δ 3.94 – 3.89 (m, 0.18H), trans isomer: δ 3.81 (tt, *J* =

12.0, 7.7 Hz, 0.87H), total 1H), 2.15 – 2.12 (m, 2H), 1.94 – 1.91 (m, 2H), 1.72 – 1.62 (m, 2H), 1.44 – 1.20 (m, 3H), 1.12 – 1.04 (m, 2H), 0.95 – 0.91 (m, 3H).

^{13}C NMR: (125 MHz, $CDCl_3$, ppm): δ 118.6, 107.5, 59.0, 38.7, 34,5, 32.0, 29.6, 11.7

HRMS: (APCI, *m/z*) calcd for $C_{12}H_{20}N$ [M+H]$^+$ 178.1590, found: 178.1589

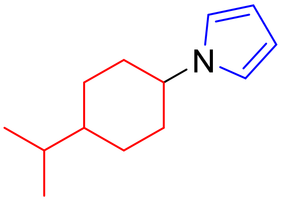

1-(4-*iso*-Propylcyclohexyl)-1*H*-pyrrole (3g):

Following the general procedure, pentane was used to work up. After column chromatography on neutral alumina (eluent: pentane), **3g** was isolated as a colorless oil (27.2 mg, 71% yield). The cis/trans isomers cannot be separated, the spectroscopic data are as follows:

^{1}H NMR: (500 MHz, $CDCl_3$, ppm): δ 6.79 – 6.74 (t, *J* = 2.1 Hz, 2H), 6.17 – 6.12 (t, *J* = 2.1 Hz, 2H), 4.02 – 3.76 (cis isomer: δ 4.02 – 3.89 (m, 0.12H), trans isomer: δ 3.80 (tt, *J* = 12.0, 7.7 Hz, 0.90H), total 1H), 2.15 – 2.14 (m, 2H), 1.90 – 1.88 (m, 2H), 1.71 – 1.63 (m, 2H), 1.59 – 1.47 (m, 1H), 1.23 – 1.14 (m, 3H), 0.92 (d, *J* = 6.8 Hz, 6H).

^{13}C NMR: (125 MHz, $CDCl_3$, ppm): δ 118.6, 107.5, 59.0, 43.4, 34,7, 32.7, 29.0, 20.0

HRMS: (APCI, *m/z*) calcd for $C_{13}H_{22}N$ [M+H]$^+$ 192.1747, found: 192.1747

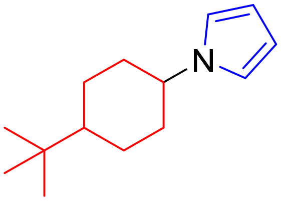

1-(4-(*tert*-Butyl)cyclohexyl)-1*H*-pyrrole (3h):

Following the general procedure, pentane was used to work up. After column chromatography on neutral alumina (eluent: pentane), **3h** was isolated as a colorless oil

(28.6 mg, 70% yield). The major isomer (trans isomer) can be separated as pure compounds, the spectroscopic data are as follows:

^{1}H NMR: (500 MHz, $CDCl_3$, ppm): δ 6.74 (t, *J* = 2.1 Hz, 2H), 6.15 (t, *J* = 2.1 Hz, 2H), 3.78 (tt, *J* = 12.0, 7.8 Hz, 1H), 2.19 – 2.15 (m, 2H), 1.94 – 1.91 (m, 2H), 1.70 – 1.62 (m, 2H), 1.23 – 1.16 (m, 2H), 1.15 – 1.07 (m, 1H), 0.90 (s, 9H).

^{13}C NMR: (125 MHz, $CDCl_3$, ppm): δ 118.6, 107.5, 58.9, 47.4, 34,9, 32.5, 27.7, 26.7

HRMS: (APCI, *m/z*) calcd for $C_{14}H_{24}N$ $[M+H]^+$ 206.1903, found: 206.1901

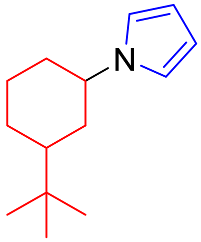

1-(3-(*tert*-Butyl)cyclohexyl)-1*H*-pyrrole (3i):

Following the general procedure, pentane was used to work up. After column chromatography on neutral alumina (eluent: pentane), **3i** was isolated as a colorless oil (25.9 mg, 63% yield). The major isomer (cis isomer) can be separated as pure compounds, the spectroscopic data are as follows:

^{1}H NMR: (500 MHz, $CDCl_3$, ppm): δ 6.75 (t, *J* = 2.1 Hz, 2H), 6.16 (t, *J* = 2.1 Hz, 2H), 3.83 (tt, *J* = 11.9, 7.5 Hz, 1H), 2.17 – 2.13 (m, 1H), 2.10 – 2.07 (m, 1H), 1.98 – 1.92 (m, 1H), 1.83 – 1.80 (m, 1H), 1.61 – 1.53 (m, 1H), 1.42 – 1.32 (m, 2H), 1.26 – 1.18 (m, 1H), 1.04 – 0.95 (m, 1H), 0.88 (s, 9H).

^{13}C NMR: (125 MHz, $CDCl_3$, ppm): δ 118.6, 107.5, 59.6, 47.8, 36.4, 34.5, 32.6, 27.7, 26.6, 25.6

HRMS: (APCI, *m/z*) calcd for $C_{14}H_{24}N$ $[M+H]^+$ 206.1903, found: 206.1901

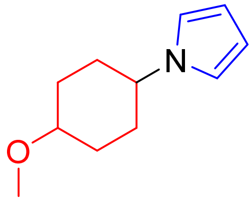

1-(4-Methoxycyclohexyl)-1*H*-pyrrole (3j):

Following the general procedure, EtOAc was used to work up. After preparative TLC isolation (eluent: hexanes: EtOAc: DCM = 40:1:2), **3j** was isolated as a colorless oil (18.3 mg, 51% yield). The cis/trans isomers can be separated as pure compounds, the spectroscopic data are as follows:

cis isomer:

^{1}H NMR: (500 MHz, $CDCl_3$, ppm): δ 6.75 (t, *J* = 2.1 Hz, 2H), 6.14 (t, *J* = 2.1 Hz, 2H), 3.84 (tt, *J* = 11.8, 3.9 Hz, 1H), 3.48 (quint, *J* = 2.9 Hz, 1H), 3.34 (s, 3H), 2.11 – 2.07 (m, 2H), 2.05 – 1.97 (m, 2H), 1.87 – 1.84 (m, 2H), 1.54 – 1.48 (m, 2H).

^{13}C NMR: (125 MHz, $CDCl_3$, ppm): δ 118.7, 107.5, 73.6, 58.2, 55.8, 28.9, 28.7

trans isomer:

^{1}H NMR: (500 MHz, $CDCl_3$, ppm): δ 6.72 (t, *J* = 2.1 Hz, 2H), 6.15 (t, *J* = 2.1 Hz, 2H), 3.86 (tt, *J* = 11.9, 7.5 Hz, 1H), 3.38 (s, 3H), 3.22 (tt, *J* = 10.9, 4.0 Hz 1H), 2.22 – 2.14 (m, 4H), 1.76 – 1.68 (m, 2H), 1.42 – 1.34 (m, 2H).

^{13}C NMR: (125 MHz, $CDCl_3$, ppm): δ 118.6, 107.8, 78.4, 57.9, 56.2, 32.3, 31.0

HRMS: (APCI, *m/z*) calcd for $C_{11}H_{18}ON$ $[M+H]^+$ 180.1383, found: 180.1384

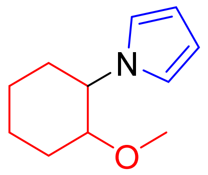

1-(2-Methoxycyclohexyl)-1*H*-pyrrole (3k):

Following the general procedure, EtOAc was used to work up. After preparative TLC isolation (eluent: hexanes: EtOAc: DCM = 20:1:1), **3k** was isolated as a colorless oil (25.6 mg, 78% yield). The cis/trans isomers can be separated as pure compounds, the spectroscopic data are as follows:

cis isomer:

^{1}H NMR: (500 MHz, $CDCl_3$, ppm): δ 6.80 (t, *J* = 2.1 Hz, 2H), 6.13 (t, *J* = 2.1 Hz, 2H), 3.87 (dt, *J* = 12.7, 6.4 Hz, 1H), 3.55 (m, 1H), 3.12 (s, 3H), 2.23 – 2.14 (m, 1H), 2.10 – 2.06 (m, 1H), 1.89 – 1.85 (m, 1H), 1.82 – 1.78 (m, 1H), 1.62 – 1.53 (m, 1H), 1.49 – 1.45 (m, 1H), 1.43 – 1.34 (m, 2H)

^{13}C NMR: (125 MHz, $CDCl_3$, ppm): δ 119.8, 107.3, 79.6, 61.5, 57.2, 28.8, 27.3, 25.6, 19.4

trans isomer:

^{1}H NMR: (500 MHz, $CDCl_3$, ppm): δ 6.75 (t, *J* = 2.1 Hz, 2H), 6.15 (t, *J* = 2.1 Hz, 2H), 3.68 – 3.62 (m, 1H), 3.15 (td, *J* = 10.0, 3.7 Hz, 1H), 2.97 (s, 3H), 2.20 – 2.16 (m, 1H), 2.11 – 2.08 (m, 1H), 1.84 – 1.82 (m, 2H), 1.79 – 1.70 (m, 1H), 1.37 – 1.25 (m, 3H).

^{13}C NMR: (125 MHz, $CDCl_3$, ppm): δ 119.3, 107.7, 83.5, 64.1, 57.7, 33.0, 31.8, 25.4, 24.6

HRMS: (APCI, *m/z*) calcd for $C_{11}H_{18}ON$ $[M+H]^+$ 180.1383, found: 180.1382

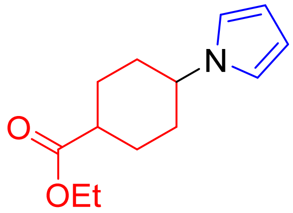

Ethyl 4-(1*H*-pyrrol-1-yl)cyclohexane-1-carboxylate (3l):

Following the general procedure, EtOAc was used to work up. After preparative TLC isolation (eluent: hexanes: EtOAc: DCM = 20:1:1), **3l** was isolated as a colorless oil (26.2 mg, 59% yield). The cis/trans isomers can be separated as pure compounds, the spectroscopic data are as follows:

cis isomer:

^{1}H NMR: (500 MHz, $CDCl_3$, ppm): δ 6.72 (t, *J* = 2.1 Hz, 2H), 6.14 (t, *J* = 2.1 Hz, 2H), 4.18 (q, *J* = 7.1 Hz, 2H), 3.86 (tt, *J* = 11.0, 4.0 Hz, 1H), 2.66 (quint, *J* = 4.1 Hz, 1H), 2.28 – 2.25 (m, 2H), 2.00 – 1.97 (m, 2H), 1.93 – 1.85 (m, 2H), 1.69 – 1.62 (m, 2H), 1.28 (t, *J* = 7.1 Hz, 3H).

^{13}C NMR: (125 MHz, $CDCl_3$, ppm): δ 174.6, 118.7, 107.7, 60.6, 57.7, 38.7, 30.9, 26.6, 14.4

trans isomer:

^{1}H NMR: (500 MHz, $CDCl_3$, ppm): δ 6.72 (t, *J* = 2.1 Hz, 2H), 6.15 (t, *J* = 2.1 Hz, 2H), 4.15 (q, *J* = 7.1Hz, 2H), 3.84 (tt, *J* = 11.7, 7.6 Hz, 1H), 2.33 (tt, *J* = 11.2, 3.5 Hz, 1H), 2.21 – 2.13 (m, 4H), 1.74 – 1.55 (m, 4H), 1.27 (t, *J* = 7.1Hz, 3H).

^{13}C NMR: (125 MHz, $CDCl_3$, ppm): δ 175.4, 118.5, 107.8, 60.6, 57.9, 42.6, 33.6, 28.4, 14.4

HRMS: (ESI, *m/z*) calcd for $C_{13}H_{19}NNaO_2$ $[M+Na]^+$ 244.1308, found: 244.1311

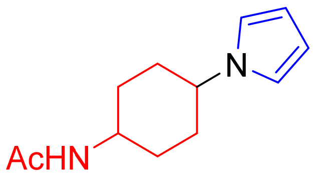

N-(4-(1H-pyrrol-1-yl)cyclohexyl)acetamide (3m):

Following the general procedure, EtOAc was used to work up. After preparative TLC isolation (eluent: hexanes: EtOAc: MeOH = 5:1:1), **3m** was isolated as a white solid (37.2 mg, 90% yield). The cis/trans isomers can be separated as pure compounds, the spectroscopic data are as follows:

cis isomer:

^{1}H NMR: (500 MHz, $CDCl_3$, ppm): δ 6.76 (t, *J* = 2.1 Hz, 2H), 6.17 (t, *J* = 2.1 Hz, 2H), 5.56 (br, 1H), 4.14 – 4.09 (m, 1H), 3.97 – 3.91 (m, 1H), 2.04 – 2.02 (m, 5H), 1.93 – 1.83 (m, 4H), 1.78 – 1.72 (m, 2H).

^{13}C NMR: (125 MHz, $CDCl_3$, ppm): δ 169.6, 118.6, 108.0, 56.2, 44.5, 29.3, 29.0, 23.8

trans isomer:

^{1}H NMR: (500 MHz, $CDCl_3$, ppm): δ 6.70 (t, *J* = 2.1 Hz, 2H), 6.14 (t, *J* = 2.1 Hz, 2H), 5.33 (br, 1H), 3.89 – 3.79 (m, 2H), 2.16 – 2.13 (m, 4H), 1.98 (s, 3H), 1.86 – 1.78 (m, 2H), 1.34 – 1.27 (m, 2H).

^{13}C NMR: (125 MHz, $CDCl_3$, ppm): δ 169.5, 118.6, 107.9, 57.4, 47.9, 33.1, 32.3, 23.7

HRMS: (APCI, *m/z*) calcd for $C_{12}H_{19}ON_2$ $[M+H]^+$ 207.1492, found: 207.1488

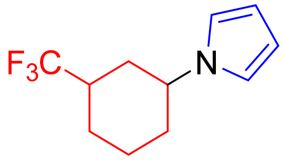

1-(3-(Trifluoromethyl)cyclohexyl)-1*H*-pyrrole (3n):

Following the general procedure, EtOAc was used to work up. After column chromatography on neutral alumina (eluent: pentane), **3n** was isolated as a colorless oil (21.6 mg, 50% yield). The major isomer (cis isomer) can be separated as pure compounds, the spectroscopic data are as follows:

^{1}H NMR: (500 MHz, $CDCl_3$, ppm): δ 6.72 (t, *J* = 2.1 Hz, 2H), 6.16 (t, *J* = 2.1 Hz, 2H), 3.88 (tt, *J* = 12.1, 3.8 Hz, 1H), 2.34 – 2.31 (m, 1H), 2.27 – 2.19 (m, 1H), 2.17 – 2.14 (m, 1H), 2.06 – 2.01 (m, 2H), 1.70 – 1.61 (m, 2H), 1.51 – 1.42 (m, 1H), 1.37 – 1.29 (m, 1H).

^{13}C NMR: (125 MHz, $CDCl_3$, ppm): δ 127.1 (q, *J* = 278.7 Hz), 118.5, 108.1, 57.3, 41.8 (q, *J* = 27.2 Hz), 33.6, 33.3, 24.3, 24.0

^{19}F NMR: (471 MHz, $CDCl_3$, ppm): δ -73.7

HRMS: (APCI, *m/z*) calcd for $C_{11}H_{15}NF_3$ $[M+H]^+$ 218.1151, found: 218.1143

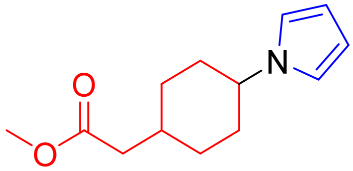

Methyl 2-(4-(1*H*-pyrrol-1-yl)cyclohexyl)acetate (3o):

Following the general procedure, EtOAc was used to work up. After preparative TLC isolation (eluent: hexanes: EtOAc: MeOH = 40:1:1), **3o** was isolated as a colorless oil (39.8 mg, 90% yield). The cis/trans isomers cannot be separated, the spectroscopic data are as follows:

^{1}H NMR: (500 MHz, CD_3OD, ppm): δ 6.77 – 6.72 (t, *J* = 2.1 Hz, 2H), 6.03 – 6.00 (t, *J* = 2.1 Hz, 2H), 3.96 – 3.80 (cis isomer: δ 3.96 – 3.91 (m, 0.19H), trans isomer: δ 3.84 (tt, *J* = 12.0, 3.9 Hz, 0.86H), total 1H), 3.66 (s, 3H), 2.44 – 2.26 (cis isomer: δ 2.44 (d, *J* =7.6

Hz, 0.35H), trans isomer: δ 2.27 (d, J = 6.9 Hz, 1.71H), total 2H), 2.06 – 2.02 (m, 2H), 2.00 – 1.80 (m, 3H), 1.77 – 1.69 (m, 2H), 1.25 – 1.17 (m, 2H).

^{13}C NMR: (125 MHz, CD_3OD, ppm): δ 175.0, 119.3, 108.2, 59.4, 51.9, 42.0, 35.3, 35.2, 33.0

HRMS: (APCI, *m/z*) calcd for $C_{13}H_{20}O_2N$ $[M+H]^+$ 222.1489, found: 222.1487

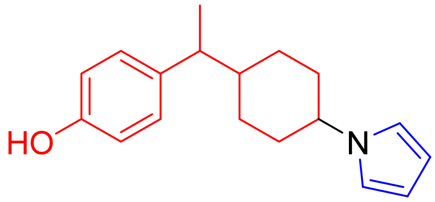

4-(1-(4-(1*H*-pyrrol-1-yl)cyclohexyl)ethyl)phenol (3p):

Following the general procedure, EtOAc was used to work up. After preparative TLC isolation (eluent: hexanes: EtOAc = 7:1), **3p** was isolated as a colorless oil (23.3 mg, 43% yield). The cis/trans isomers cannot be separated, the spectroscopic data are as follows:

^{1}H NMR: (500 MHz, $CDCl_3$, ppm): δ 7.03 – 7.04 (m, 2H), 6.79 – 6.75 (m, 2H), 6.7 (t, J = 2.1 Hz, 2H), 6.17 – 6.12 (t, J = 2.1 Hz, 2H), 4.61 (s, 1H), 3.98 – 3.71 (cis isomer: δ 3.98 – 3.93 (m, 0.21H), trans isomer: δ 3.74 (tt, J = 12.0, 7.7 Hz, 0.85H), total 1H), 2.73 – 2.41 (m, 1H), 2.16 – 2.13 (m, 1H), 2.06 – 2.02 (m, 2H), 1.96 – 1.72 (m, 1H), 1.71 – 1.61 (m, 2H), 1.47 – 1.31 (m, 1H), 1.25 – 1.20 (trans isomer: δ 1.25 (d, J = 7.1 Hz, 2.54 H), cis isomer: δ 1.21 (d, J = 6.9 Hz, 0.65 H), total 3H), 1.17 – 1.08 (m, 1H), 1.05 – 0.96 (m, 1H).

^{13}C NMR: (125 MHz, $CDCl_3$, ppm): δ 153.8, 138.8, 128.8, 118.6, 115.1, 107.5, 58.8, 44.8, 43.6, 34.6, 34.4, 30.3, 29.9, 19.3

HRMS: (APCI, *m/z*) calcd for $C_{18}H_{24}ON$ $[M+H]^+$ 270.1852, found: 270.1853

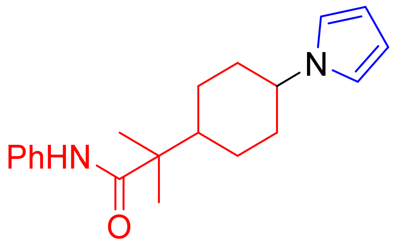

2-(4-(1*H*-pyrrol-1-yl)cyclohexyl)-2-methyl-*N*-phenylpropanamide (3q):

Following the general procedure, EtOAc was used to work up. After preparative TLC isolation (eluent: hexanes: EtOAc: DCM = 1:5:1), **3q** was isolated as a white solid (29.4 mg, 47% yield). The major isomer (trans isomer) can be separated as pure compound, the spectroscopic data are as follows:

^{1}H NMR: (500 MHz, $CDCl_3$, ppm): δ 7.55 – 7.53 (m, 2H), 7.35 – 7.32 (m, 3H), 7.14 – 7.11 (m, 1H), 6.71 (t, J = 2.1 Hz, 2H), 6.14 (t, J = 2.1 Hz, 2H), 3.80 (tt, J = 12.0, 7.8 Hz, 1H), 2.20 – 2.16 (m, 2H), 1.89 – 1.87 (m, 2H), 1.83 – 1.68 (m, 3H), 1.35 – 1.26 (m, 8H).

^{13}C NMR: (125 MHz, $CDCl_3$, ppm): δ 176.0, 137.9, 129.2, 124.6, 120.2, 118.6, 107.7, 58.6, 45.9, 45.2, 34.5, 26.8, 22.5

HRMS: (APCI, *m/z*) calcd for $C_{20}H_{27}ON_2$ $[M+H]^+$ 311.2118, found: 311.2116

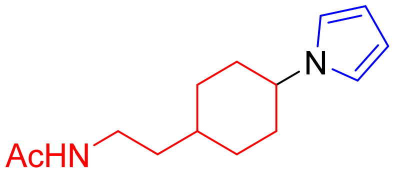

***N*-(2-(4-(1*H*-pyrrol-1-yl)cyclohexyl)ethyl)acetamide (3r):**

Following the general procedure, EtOAc was used to work up. After column chromatography on neutral alumina (eluent: DCM: MeOH = 96: 4), **3r** was isolated as a white solid (28.6 mg, 70% yield). The cis/trans isomers cannot be separated, the spectroscopic data are as follows:

^{1}H NMR: (500 MHz, $CDCl_3$, ppm): δ 6.75 – 6.71 (t, J = 2.1 Hz, 2H), 6.15 – 6.13 (t, J = 2.1 Hz, 2H), 5.48 (br, 1H), 3.94 – 3.76 (cis isomer: δ 3.94 – 3.89 (m, 0.18H), trans isomer: δ 3.79 (tt, J = 12.0, 7.7 Hz, 0.84H), total 1H), 3.32 – 3.27 (m, 2H), 2.14 – 2.11 (m, 2H), 1.98 (s, 3H), 1.94 – 1.90 (m, 2H), 1.71 – 1.55 (m, 3H), 1.48 – 1.33 (m, 2H), 1.17 – 1.08 (m, 2H).

^{13}C NMR: (125 MHz, $CDCl_3$, ppm): δ 170.2, 118.5, 107.6, 58.7, 37.3, 36.7, 34.7, 34.3, 32.2, 23.5

HRMS: (APCI, *m/z*) calcd for $C_{14}H_{23}ON_2$ $[M+H]^+$ 235.1805, found: 235.1803

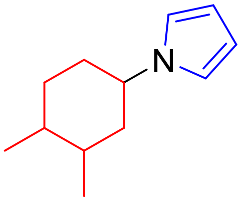

1-(3,4-Dimethylcyclohexyl)-1*H*-pyrrole (3s):

Following the general procedure, pentane was used to work up. After column chromatography on neutral alumina (eluent: pentane), **3s** was isolated as colorless oil (21.2 mg, 60% yield). The isomers cannot be separated, the spectroscopic data are as follows:

^{1}H NMR: (500 MHz, $CDCl_3$, ppm): δ 6.80 – 6.72 (m, 2H), 6.17 – 6.13 (m, 2H), 4.20 – 3.83 (isomer 1: δ 4.20 – 4.16 (m, 0.07H), isomer 2: δ 4.02 (tt, *J* = 12.0, 8.1 Hz, 0.09H), isomer 3: δ 3.86 (tt, *J* = 12.0, 7.6 Hz, 0.85H), total 1H), 2.11 – 2.03 (m, 2H), 1.84 – 1.79 (m, 1H), 1.71 – 1.63 (m, 1H), 1.45 – 1.37 (m, 1H), 1.23 – 1.13 (m, 2H), 1.10 – 1.01 (m, 1H), 0.97 – 0.95 (m, 6H).

^{13}C NMR: (125 MHz, $CDCl_3$, ppm): δ 118.5, 107.5, 58.7, 43.4, 38.53, 38.47, 34.63, 34.61, 20.1, 19.7

HRMS: (APCI, *m/z*) calcd for $C_{12}H_{20}N$ $[M+H]^+$ 178.1590, found: 178.1589

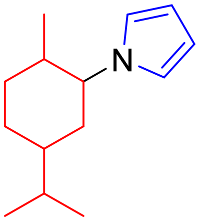

1-(5-*iso*-Propyl-2-methylcyclohexyl)-1*H*-pyrrole (3t):

Following the general procedure, pentane was used to work up. After column chromatography on neutral alumina (eluent: pentane), **3t** was isolated as colorless oil (16.8 mg, 41% yield). The isomers cannot be separated, the spectroscopic data are as follows:

^{1}H NMR: (500 MHz, $CDCl_3$, ppm): δ 6.72 – 6.67 (m, 2H), 6.15 – 6.13 (m, 2H), 4.10 – 3.32 (isomer 1: δ 4.07 (dt, *J* = 12.7, 8.3 Hz, 0.71H), isomer 2: δ 3.52 – 3.47 (m, 0.29H), isomer 3: δ 3.35 (td, *J* = 11.4, 3.7 Hz, 0.21H), total 1H), 2.26 – 2.01 (m, 1H), 1.91 – 1.86 (m, 1H), 1.72 – 1.64 (m, 2H), 1.52 – 1.40 (m, 2H), 1.29 – 1.17 (m, 3H), 0.94 – 0.86 (m, 6H), 0.71 – 0.68 (m, 3H).

^{13}C NMR: (125 MHz, $CDCl_3$, ppm): δ 119.1, 107.3, 61.2, 44.4, 35.3, 32.9, 31.6, 28.8, 23.1, 20.0, 19.9, 11.9

HRMS: (APCI, *m/z*) calcd for $C_{14}H_{24}N$ $[M+H]^+$ 206.1903, found: 206.1905

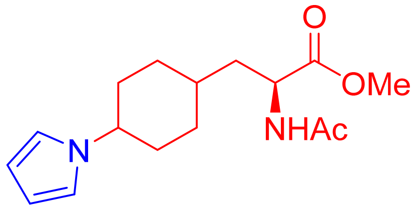

Methyl (S)-3-(4-(1*H*-pyrrol-1-yl)cyclohexyl)-2-acetamidopropanoate (3u):

Following the general procedure, EtOAc was used to work up. After column chromatography on neutral alumina (eluent: DCM: MeOH = 96: 4), **3u** was isolated as colorless solid (30.2 mg, 34% yield). The isomers cannot be separated, the spectroscopic data are as follows:

^{1}H NMR: (500 MHz, $CDCl_3$, ppm): δ 6.76 – 6.70 (m, 2H), 6.16 – 6.13 (m, 2H), 5.88 (d, *J* = 8.2 Hz, 1H), 4.73 – 4.66 (m, 1H), 3.95 – 3.77 (cis isomer: δ 3.95 – 3.90 (m, 0.24 H), trans isomer: δ 3.79 (tt, *J* = 12.0, 3.8 Hz, 0.77H), total 1H), 3.76 (s, 3H), 2.14 – 2.11 (m, 2H), 2.05 (s, 3H), 1.94 – 1.85 (m, 2H), 1.68 – 1.65 (m, 2H), 1.61 – 1.57 (m, 2H), 1.45 – 1.38 (m, 1H), 1.21 – 1.06 (m, 2H).

^{13}C NMR: (125 MHz, $CDCl_3$, ppm): δ 173.6, 170.0, 118.6, 107.6, 58.6, 52.6, 50.3, 40.1, 34.4, 34.0, 33.6, 32.5, 31.7, 23.4

HRMS: (APCI, *m/z*) calcd for $C_{16}H_{25}O_3N_2$ $[M+H]^+$ 293.1860, found: 293.1855

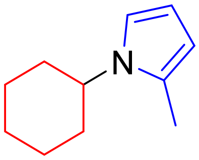

1-Cyclohexyl-2-methyl-1*H*-pyrrole (6a):

Following the general procedure, pentane was used to work up. After column chromatography on neutral alumina (eluent: pentane), **6a** was isolated as colorless oil (28.6 mg, 88% yield), the spectroscopic data are as follows:

^{1}H NMR: (500 MHz, $CDCl_3$, ppm): δ 6.68 (t, *J* = 2.3 Hz, 1H), 6.08 (t, *J* = 3.1 Hz, 1H), 5.87 – 5.86 (m, 1H), 3.78 (tt, *J* = 11.8, 7.4 Hz, 1H), 2.24 (s, 3H), 2.02 – 1.99 (m, 2H), 1.92 – 1.88 (m, 2H), 1.77 – 1.74 (m, 1H), 1.83 – 1.55 (m, 2H), 1.46 – 1.37 (m, 2H), 1.29 – 1.20 (m, 1H).

^{13}C NMR: (125 MHz, $CDCl_3$, ppm): δ 127.9, 115.9, 106.6, 106.2, 55.2, 34.5, 26.1, 25.7, 12.3

HRMS: (APCI, *m/z*) calcd for $C_{11}H_{18}N$ $[M+H]^+$ 164.1434, found: 164.1431

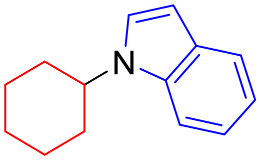

1-Cyclohexyl-1*H*-indole (6b):[4]

Following the general procedure, EtOAc was used to work up. After preparative TLC isolation (eluent: hexanes), **6b** was isolated as colorless oil (35.8 mg, 90% yield), the spectroscopic data are as follows:

^{1}H NMR: (500 MHz, $CDCl_3$, ppm): δ 6.64 – 7.62 (m, 1H), 7.39 (d, *J* = 8.2 Hz, 1H), 7.23 (d, *J* = 3.2 Hz, 1H), 7.21 – 7.18 (m, 1H), 7.11 – 7.08 (m, 1H), 6.51 (d, *J* = 3.2 Hz, 1H), 4.23 (tt, *J* = 11.9, 7.4 Hz, 1H), 2.16 – 2.13 (m, 2H), 1.97 – 1.93 (m, 2H), 1.82 – 1.79 (m, 1H), 1.76 – 1.68 (m, 2H), 1.55 – 1.47 (m, 2H), 1.35 – 1.26 (m, 1H).

^{13}C NMR: (125 MHz, $CDCl_3$, ppm): δ 135.6, 128.6, 124.2, 121.2, 121.1, 119.3, 109.6, 101.1, 55.2, 33.7, 26.1, 25.8

HRMS: (APCI, *m/z*) calcd for $C_{14}H_{18}N$ $[M+H]^+$ 200.1434, found: 200.1440

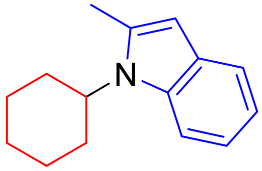

1-Cyclohexyl-2-methyl-1*H*-indole (6c):[5]

Following the general procedure, EtOAc was used to work up. After preparative TLC isolation (eluent: hexanes), **6c** was isolated as yellow oil (28.3 mg, 66% yield), the spectroscopic data are as follows:

^{1}H NMR: (500 MHz, $CDCl_3$, ppm): δ 7.53 (d, *J* = 8.8 Hz, 2H), 7.13 – 7.10 (m, 1H), 7.07 – 7.04 (m, 1H), 6.24 (s, 1H), 4.21 – 4.16 (m, 1H), 2.47 (s, 3H), 2.33 – 2.26 (m, 2H), 2.00 – 1.97 (m, 2H), 1.94 – 1.91 (m, 2H), 1.84 – 1.81 (m, 1H), 1.53 – 1.44 (m, 2H), 1.40 – 1.31 (m, 1H).

^{13}C NMR: (125 MHz, $CDCl_3$, ppm): δ 136.4, 135.8, 128.7, 120.0, 119.9, 118.9, 111.4, 100.6, 55.9, 31.7, 26.7, 25.8, 14.3

HRMS: (APCI, *m/z*) calcd for $C_{15}H_{20}N$ $[M+H]^+$ 214.1590, found: 214.1585

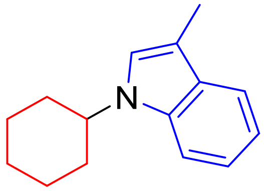

1-Cyclohexyl-3-methyl-1*H*-indole (6d):[6]

Following the general procedure, EtOAc was used to work up. After preparative TLC isolation (eluent: hexanes), **6d** was isolated as colorless oil (31.9 mg, 75% yield), the spectroscopic data are as follows:

^{1}H NMR: (500 MHz, $CDCl_3$, ppm): δ 7.60 (d, *J* = 7.9 Hz, 1H), 7.36 (d, *J* = 8.3 Hz, 1H), 7.23 – 7.20 (m, 1H), 7.13 – 7.10 (m, 1H), 7.02 (s, 1H), 4.19 (tt, *J* = 11.9, 7.5 Hz, 1H), 2.36 (d, *J* = 1.0 Hz, 3H), 2.15 – 2.12 (m, 2H), 1.97 – 1.93 (m, 2H), 1.83 – 1.80 (m, 1H), 1.75 – 1.67 (m, 2H), 1.56 – 1.47 (m, 2H), 1.35 – 1.26 (m, 1H).

^{13}C NMR: (125 MHz, $CDCl_3$, ppm): δ 135.9, 128.7, 121.9, 121.2, 119.1, 118.6, 110.2, 109.3, 54.9, 33.7, 26.2, 25.8, 9.8

HRMS: (APCI, *m/z*) calcd for $C_{15}H_{20}N$ $[M+H]^+$ 214.1590, found: 214.1588

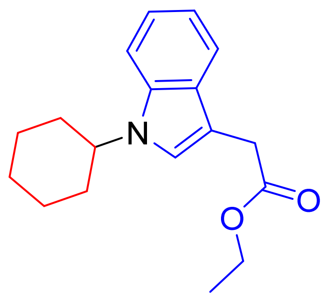

Ethyl 2-(1-cyclohexyl-1*H*-indol-3-yl)acetate (6e):

Following the general procedure, EtOAc was used to work up. After preparative TLC isolation (eluent: hexanes: EtOAc = 20:1), **6e** was isolated as colorless oil (25.8 mg, 45% yield), the spectroscopic data are as follows:

^{1}H NMR: (500 MHz, $CDCl_3$, ppm): δ 7.63 (d, J = 8.0 Hz, 1H), 7.36 (d, J = 8.3 Hz, 1H), 7.22 – 7.21 (m, 2H), 7.13 – 7.10 (m, 1H), 4.23 – 4.15 (m, 3H), 3.77 (s, 2H), 2.16 – 2.13 (m, 2H), 1.96 – 1.93 (m, 2H), 1.81 – 1.79 (m, 1H), 1.75 – 1.67 (m, 2H), 1.55 – 1.45 (m, 2H), 1.34 – 1.26 (m, 4H).

^{13}C NMR: (125 MHz, $CDCl_3$, ppm): δ 172.3, 135.9, 127.8, 123.2, 121.4, 119.24, 119.17, 109.6, 107.0, 60.8, 55.2, 33.7, 31.7, 26.1, 25.8, 14.4

HRMS: (ESI, *m/z*) calcd for $C_{18}H_{23}NNaO_2$ $[M+Na]^+$ 308.1621, found: 308.1617

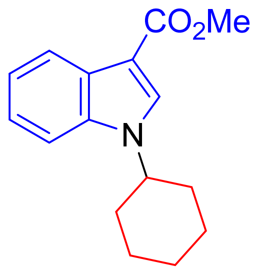

Methyl 1-cyclohexyl-1*H*-indole-3-carboxylate (6f):[7]

Following the general procedure, EtOAc was used to work up. After preparative TLC isolation (eluent: hexanes: EtOAc = 3:1), **6f** was isolated as yellow oil (4.2 mg, 8% yield), the spectroscopic data are as follows:

^{1}H NMR: (500 MHz, $CDCl_3$, ppm): δ 8.19 – 8.17 (m, 1H), 7.95 (s, 1H), 7.42 – 7.40 (m, 1H), 7.29 – 7.23 (m, 2H), 4.24 (tt, *J* = 11.9, 7.3 Hz, 1H), 3.91 (s, 3H), 2.19 – 2.17 (m, 2H), 1.98 – 1.96 (m, 2H), 1.83 – 1.80 (m, 1H), 1.76 – 1.88 (m, 2H), 1.53 – 1.47 (m, 2H), 1.35 – 1.27 (m, 1H).

^{13}C NMR: (125 MHz, $CDCl_3$, ppm): δ 165.8, 136.3, 131.1, 126.8, 122.6, 122.0, 121.9, 110.1, 107.1, 55.8, 51.1, 33.6, 25.9, 25.6

HRMS: (ESI, *m/z*) calcd for $C_{16}H_{19}NNaO_2$ $[M+Na]^+$ 280.1308, found: 280.1315

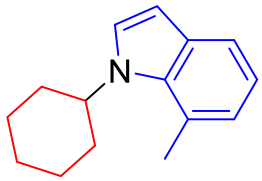

1-Cyclohexyl-7-methyl-1*H*-indole (6g):

Following the general procedure, EtOAc was used to work up. After preparative TLC isolation (eluent: hexanes), **6g** was isolated as colorless oil (14.0 mg, 33% yield), the spectroscopic data are as follows:

^{1}H NMR: (500 MHz, $CDCl_3$, ppm): δ 7.48 (d, *J* = 7.8 Hz, 1H), 7.25 (d, *J* = 3.1 Hz, 1H), 7.00 (t, *J* = 7.7 Hz, 1H), 6.92 (d, *J* = 7.1 Hz, 1H), 6.51 (d, *J* = 3.3 Hz, 1H), 4.70 (tt, *J* = 11.8, 7.0 Hz, 1H), 2.75 (s, 3H), 2.18 – 2.15 (m, 2H), 1.96 – 1.93 (m, 2H), 1.82 – 1.78 (m, 1H), 1.75 – 1.67 (m, 2H), 1.54 – 1.45 (m, 2H), 1.33 – 1.25 (m, 1H).

^{13}C NMR: (125 MHz, $CDCl_3$, ppm): δ 134.7, 129.4, 124.9, 124.5, 120.7, 119.4, 119.2, 101.8, 56.5, 35.2, 26.2, 25.8, 20.9

HRMS: (APCI, *m/z*) calcd for $C_{15}H_{20}N$ $[M+H]^+$ 214.1590, found: 214.1592

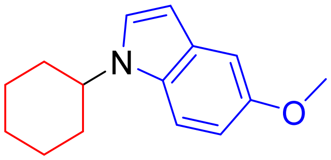

1-Cyclohexyl-5-methoxy-1*H*-indole (6h):

Following the general procedure, EtOAc was used to work up. After preparative TLC isolation (eluent: hexanes), **6h** was isolated as colorless oil (34.1 mg, 74% yield), the spectroscopic data are as follows:

^{1}H NMR: (500 MHz, $CDCl_3$, ppm): δ 7.30 (d, *J* = 8.9 Hz, 1H), 7.21 (d, *J* = 3.2 Hz, 1H), 7.12 (d, *J* = 2.5 Hz, 1H), 6.89 (dd, *J* = 8.9, 2.5 Hz, 1H), 6.45 (d, *J* = 3.1 Hz, 1H), 4.18 (tt, *J* = 11.8, 7.0 Hz, 1H), 3.87 (s, 3H), 2.17 – 2.14 (m, 2H), 1.97 – 1.94 (m, 2H), 1.83 – 1.80 (m, 1H), 1.75 – 1.67 (m, 2H), 1.56 – 1.46 (m, 2H), 1.35 – 1.26 (m, 1H).

^{13}C NMR: (125 MHz, $CDCl_3$, ppm): δ 154.0, 131.0, 128.8, 124.7, 111.6, 110.3, 102.6, 100.6, 56.0, 55.4, 33.7, 26.1, 25.8

HRMS: (APCI, *m/z*) calcd for $C_{15}H_{20}ON$ $[M+H]^+$ 230.1539, found: 230.1539

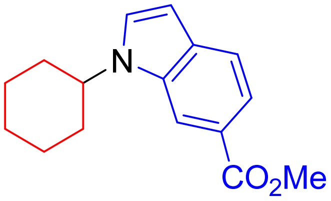

Methyl 1-cyclohexyl-1*H*-indole-6-carboxylate (6i):

Following the general procedure, EtOAc was used to work up. After preparative TLC isolation (eluent: hexanes: EtOAc = 20:1), **6i** was isolated as colorless oil (19.9 mg, 39% yield), the spectroscopic data are as follows:

^{1}H NMR: (500 MHz, $CDCl_3$, ppm): δ 8.15 (s, 1H), 7.78 (dd, *J* = 8.3, 1.4 Hz, 1H), 7.63 (d, *J* = 8.3 Hz, 1H), 7.38 (d, *J* = 3.2 Hz, 1H), 6.55 (d, *J* = 2.9 Hz, 1H), 4.33 (tt, *J* = 11.9, 7.4 Hz, 1H), 3.96 (s, 3H), 2.16 – 2.13 (m, 2H), 1.97 – 1.94 (m, 2H), 1.83 – 1.81 (m, 1H), 1.76 – 1.68 (m, 2H), 1.59 – 1.50 (m, 2H), 1.35 – 1.26 (m, 1H).

^{13}C NMR: (125 MHz, $CDCl_3$, ppm): δ 168.5, 135.0, 132.2, 127.6, 122.9, 120.5, 120.4, 112.0, 101.6, 55.3, 52.1, 33.9, 26.0, 25.7

HRMS: (ESI, *m/z*) calcd for $C_{16}H_{19}NNaO_2$ $[M+H]^+$ 280.1308, found: 280.1307

VII. References

[1] V. Gotor-Fernández, P. Fernández-Torres, V. Gotor, *Tetrahedron: Asym.* **2006**, *17*, 2558-2564.
[2] J. Pietruszka, R. C. Simon, *Chem. Eur. J.* **2010**, *16*, 14534-14544.
[3] M. Abid, S. M. Landge, B. Torok, *Org. Prep. Proc. Int.* **2006**, *38*, 495-500.
[4] J. O. Madsen, M. Meldal, S. Mortensen, B. Olsson, *Act. Chem. Scand., Ser. B* **1981**, *B 35*, 77-81.
[5] F. Peng, M. McLaughlin, Y. Liu, I. Mangion, D. M. Tschaen, Y. Xu, *J. Org. Chem.* **2016**, *81*, 10009-10015.
[6] H. Schirok, *Synthesis* **2008**, 1404-1414.
[7] F. S. Melkonyan, A. V. Karchava, M. A. Yurovskaya, *J. Org. Chem.* **2008**, *73*, 4275-4278.

VIII. NMR Spectra of Products

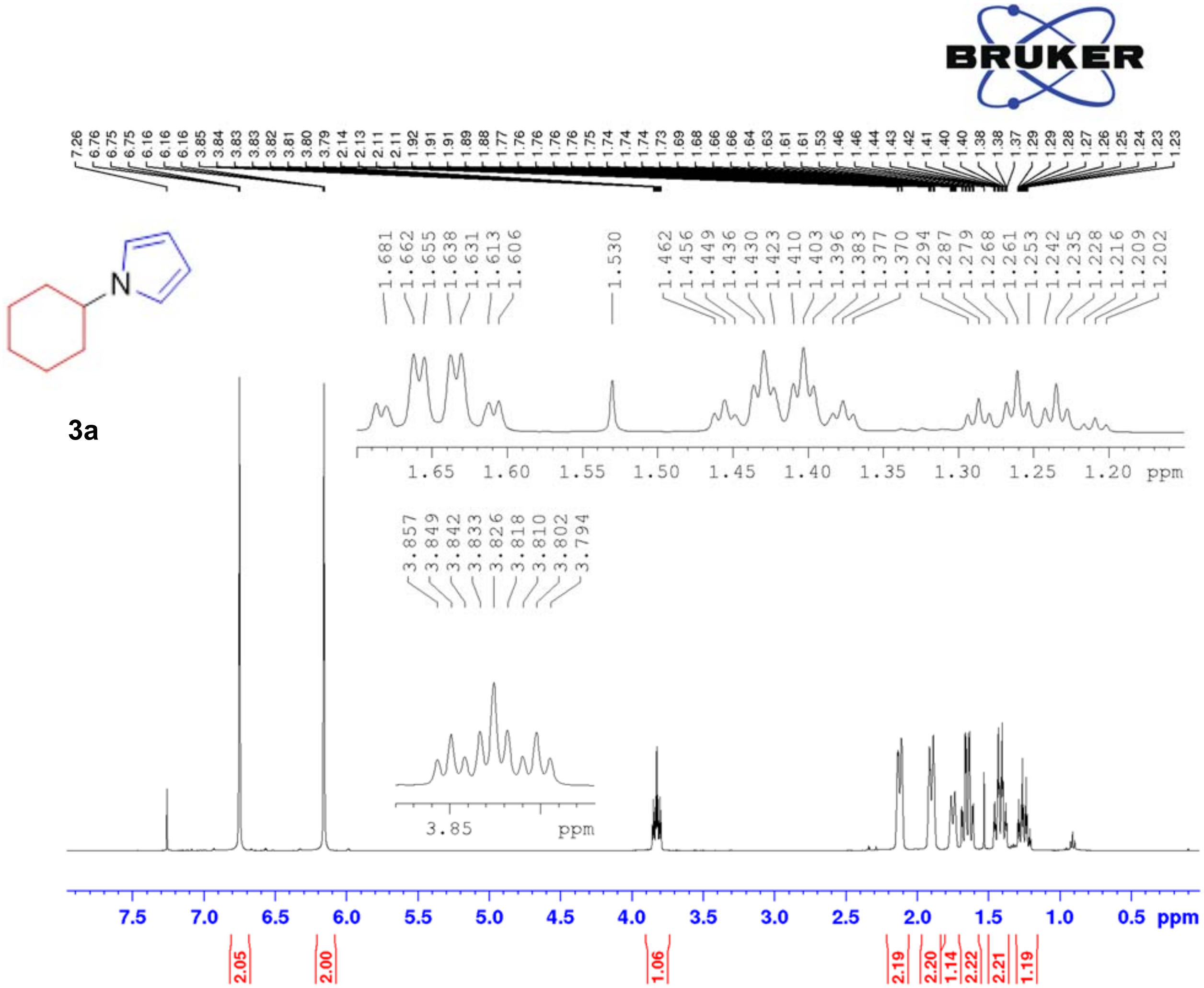

3a

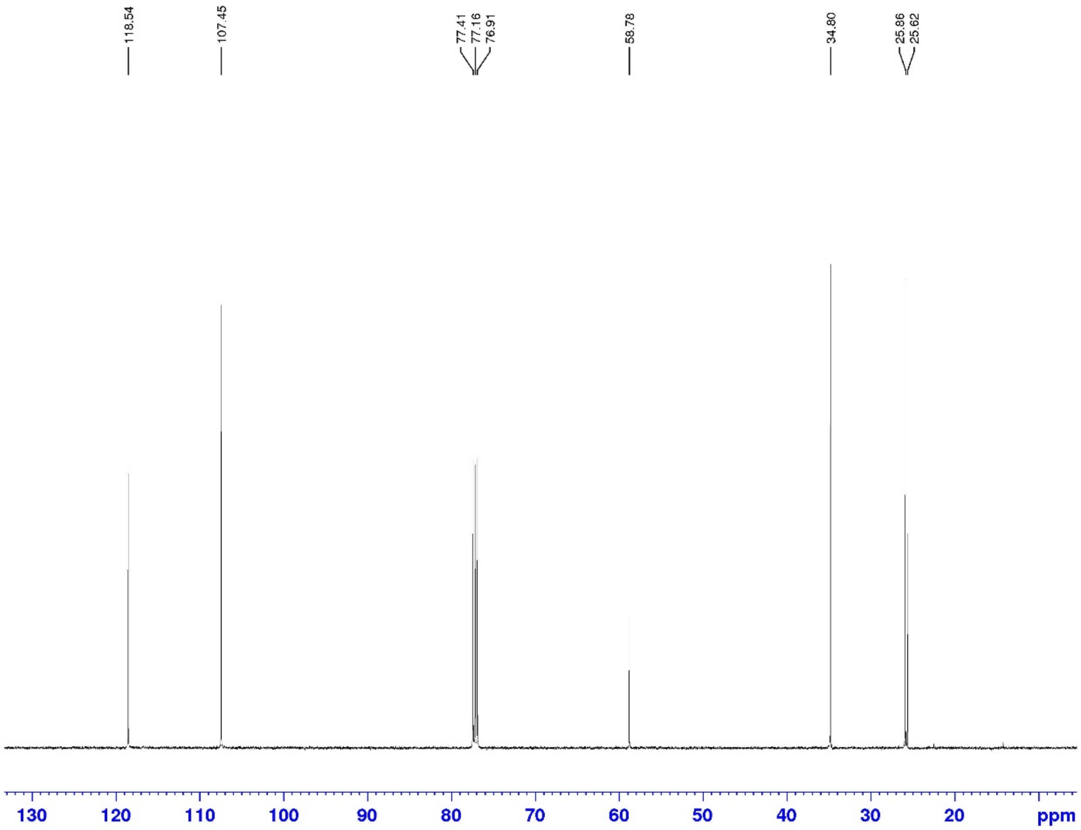
118.54
107.45
77.41
77.16
76.91
58.78
34.80
25.86
25.62
130
120
110
100
90
80
70
60
50
40
30
20
ppm

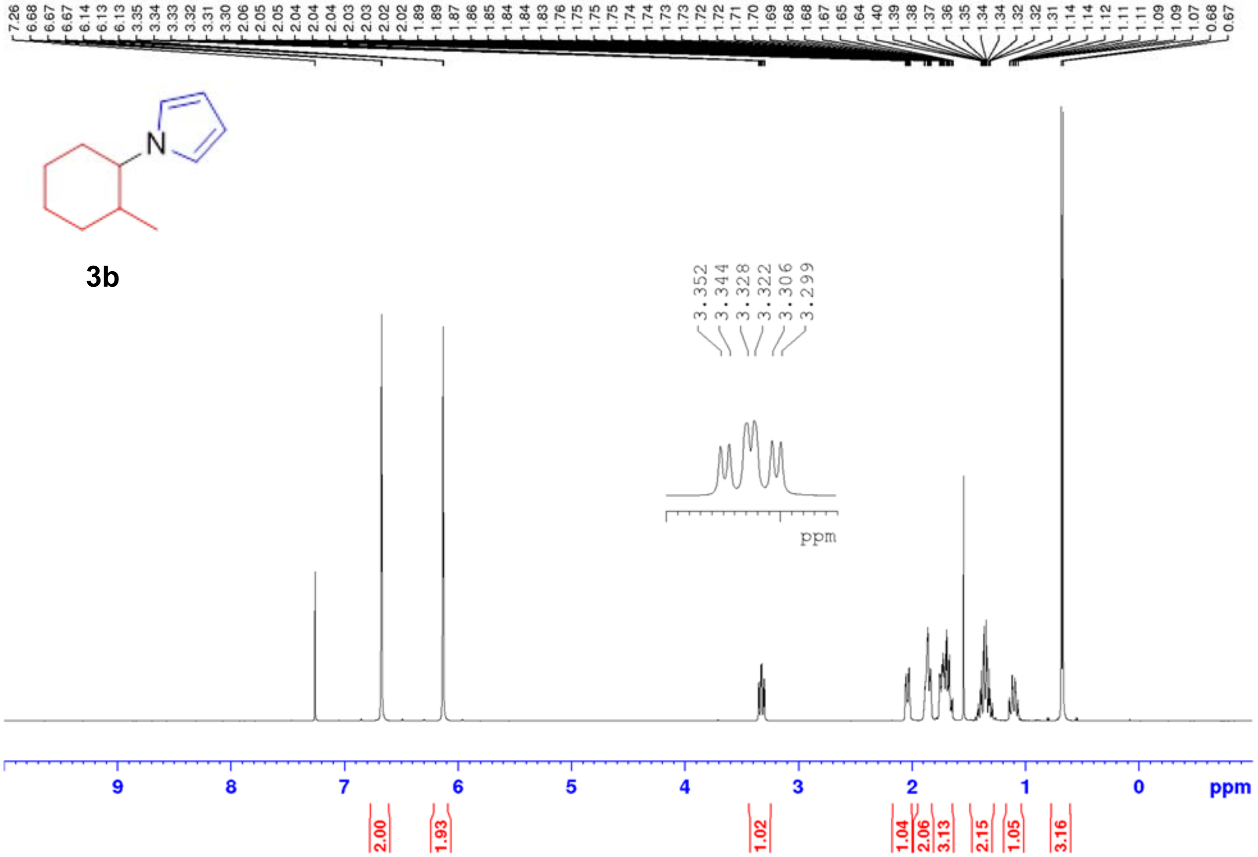
7.26 6.68 6.67 6.67 6.14 6.13 6.13 3.35 3.34 3.33 3.32 3.31 3.30 2.06 2.05 2.05 2.04 2.04 2.04 2.03 2.03 2.02 2.02 1.89 1.89 1.87 1.86 1.85 1.84 1.84 1.83 1.76 1.75 1.75 1.75 1.74 1.74 1.73 1.73 1.72 1.72 1.71 1.70 1.69 1.68 1.68 1.67 1.65 1.64 1.40 1.39 1.38 1.37 1.36 1.35 1.34 1.34 1.32 1.32 1.31 1.14 1.14 1.12 1.11 1.11 1.09 1.09 1.07 0.68 0.67
N
3.352
3.344
3.328
3.322
3.306
3.299
ppm
9 8 7 6 5 4 3 2 1 0 ppm
2.00 1.93 1.02 1.04 2.06 3.13 2.15 1.05 3.16

3b

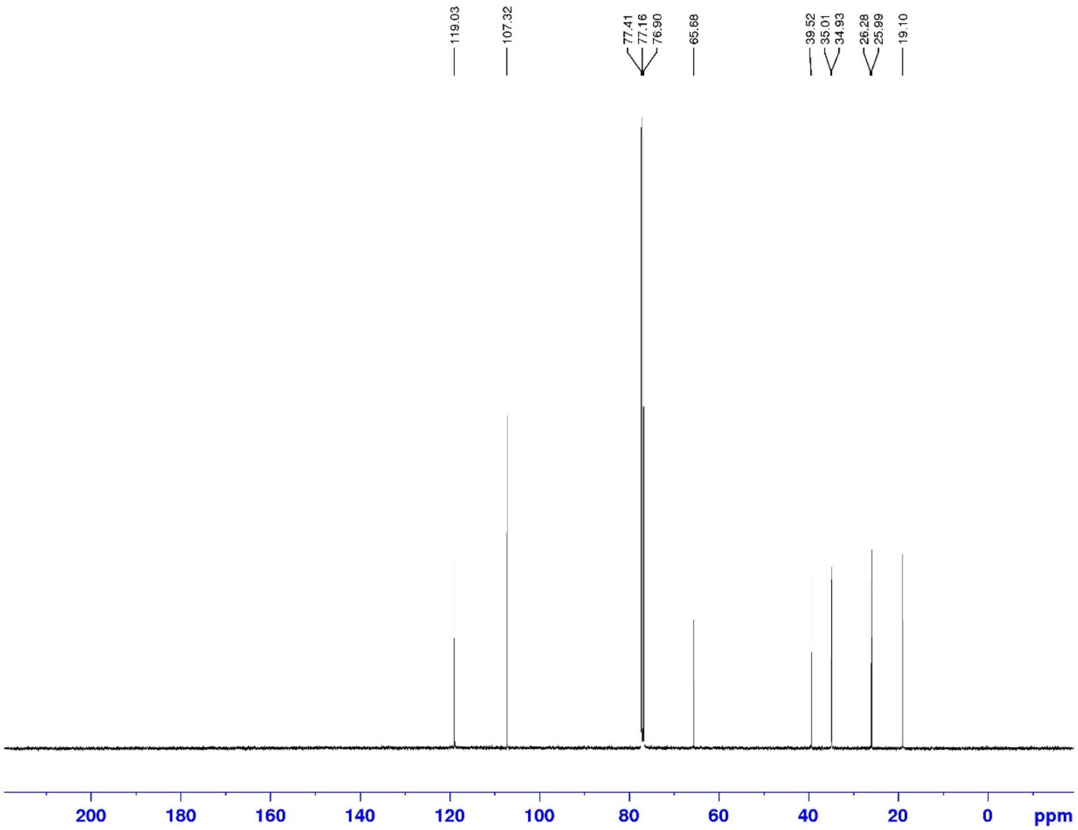
119.03
107.32
77.41
77.16
76.90
65.68
39.52
35.01
34.93
26.28
25.99
19.10
200
180
160
140
120
100
80
60
40
20
0
ppm

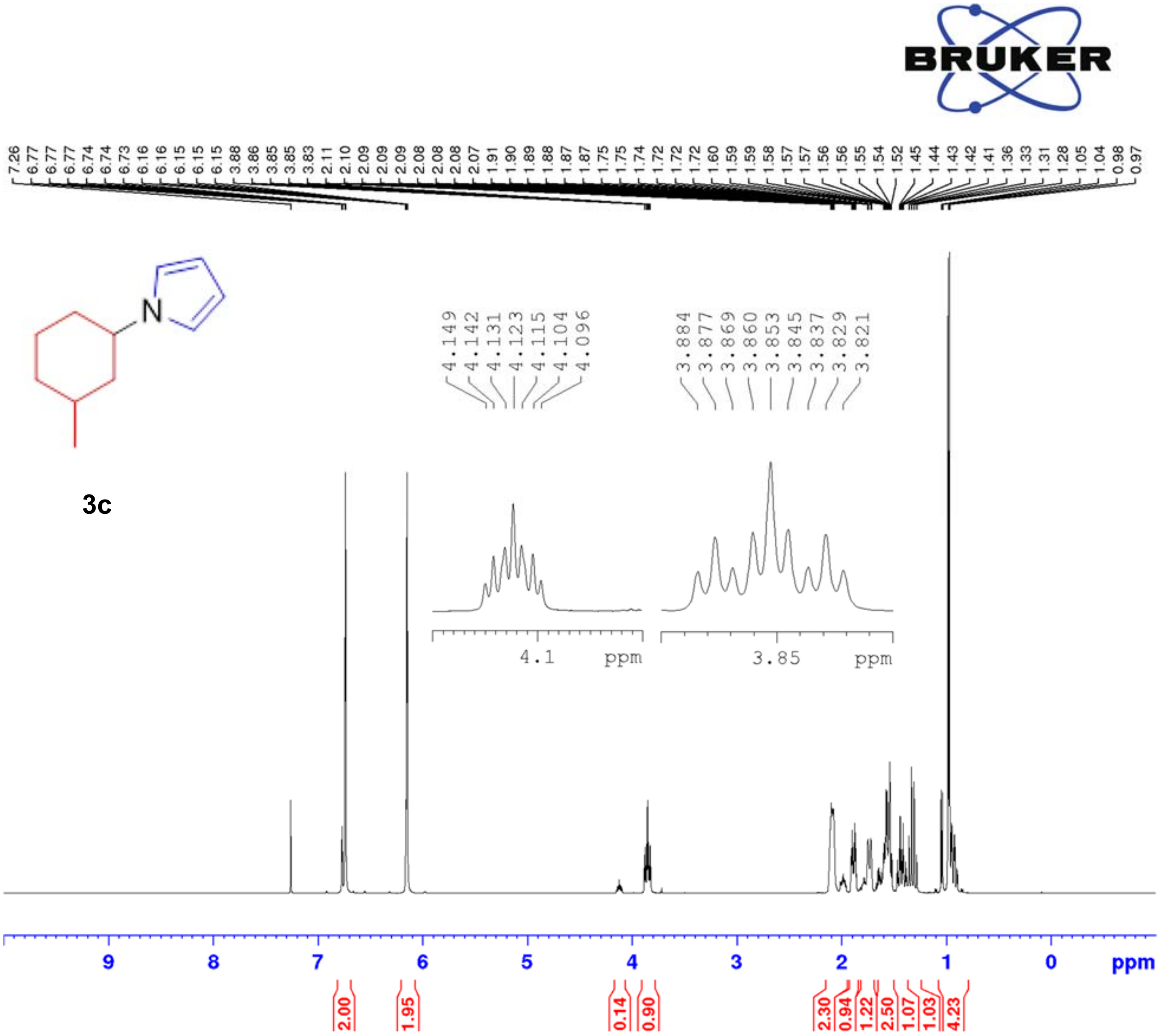
3c
ppm

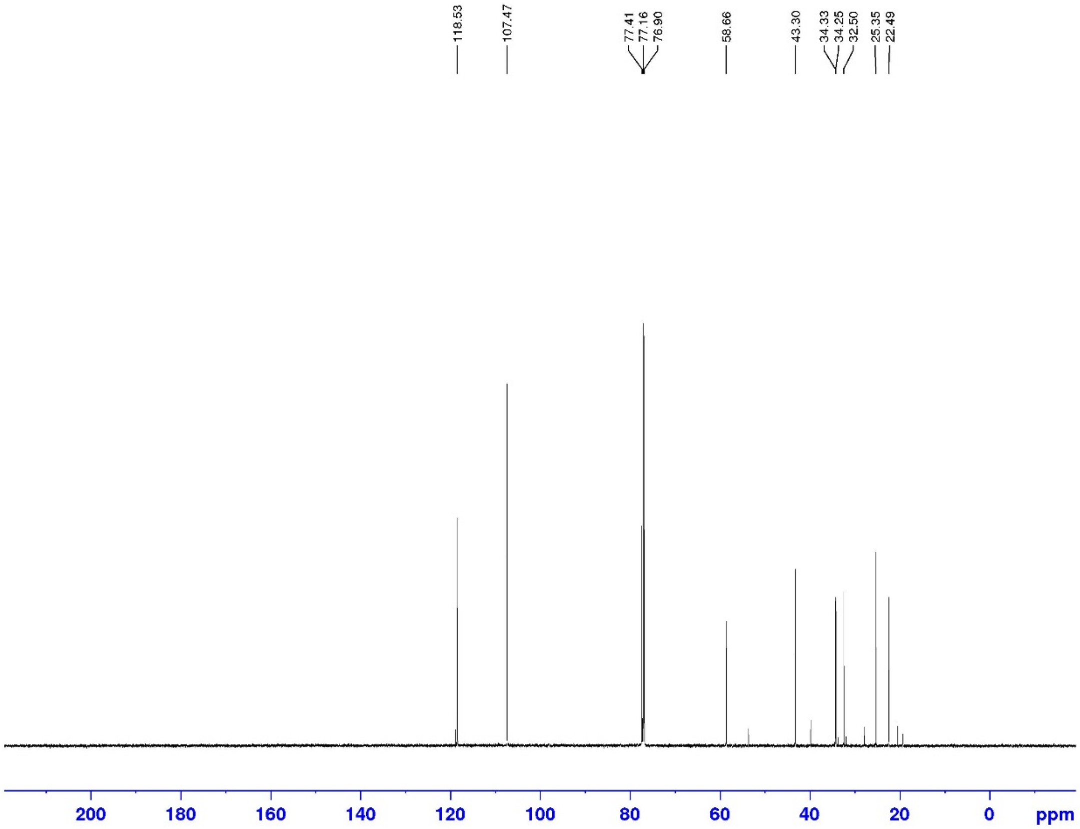
118.53
107.47
77.41
77.16
76.90
58.66
43.30
34.33
34.25
32.50
25.35
22.49
200
180
160
140
120
100
80
60
40
20
0
ppm

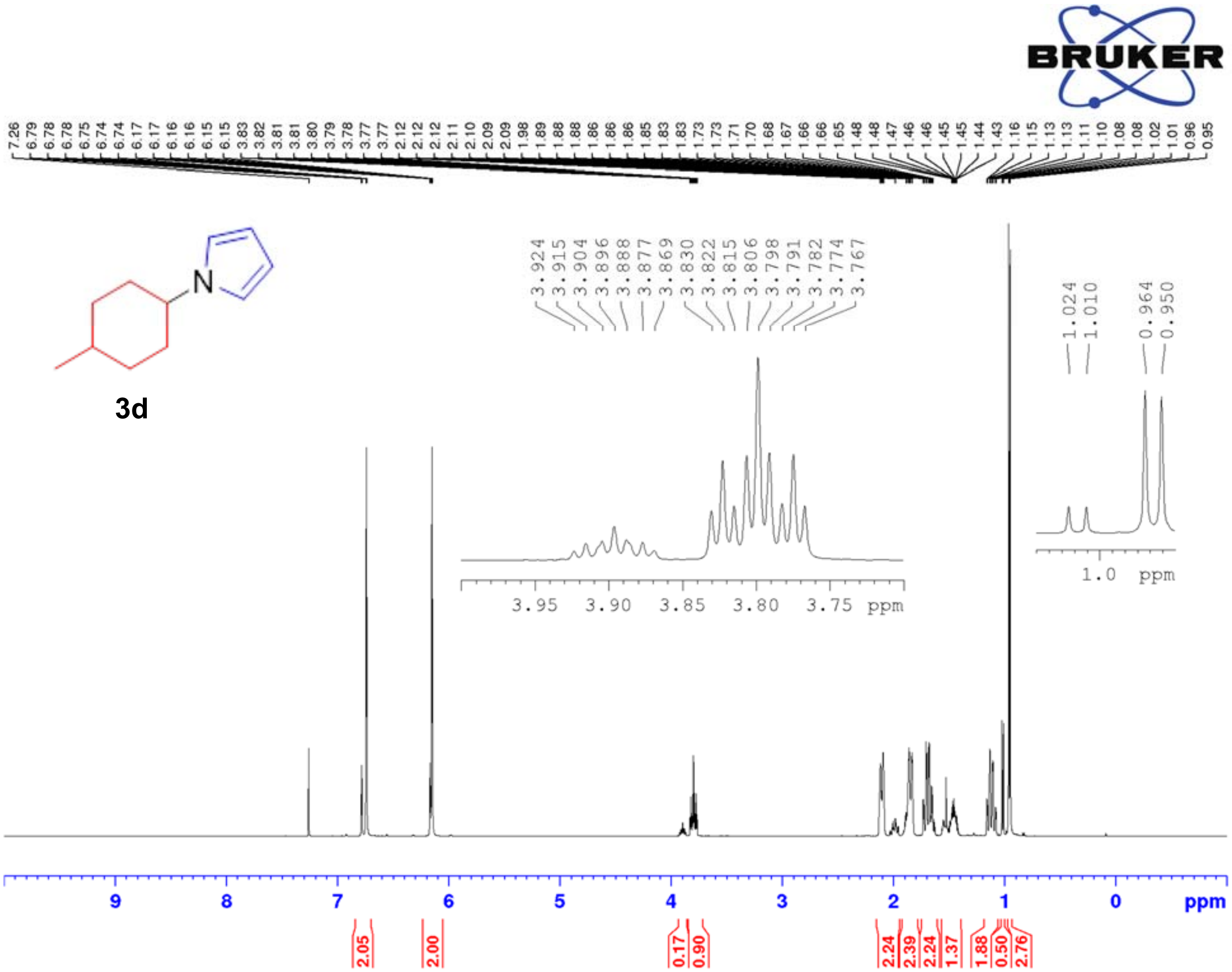
3d
ppm

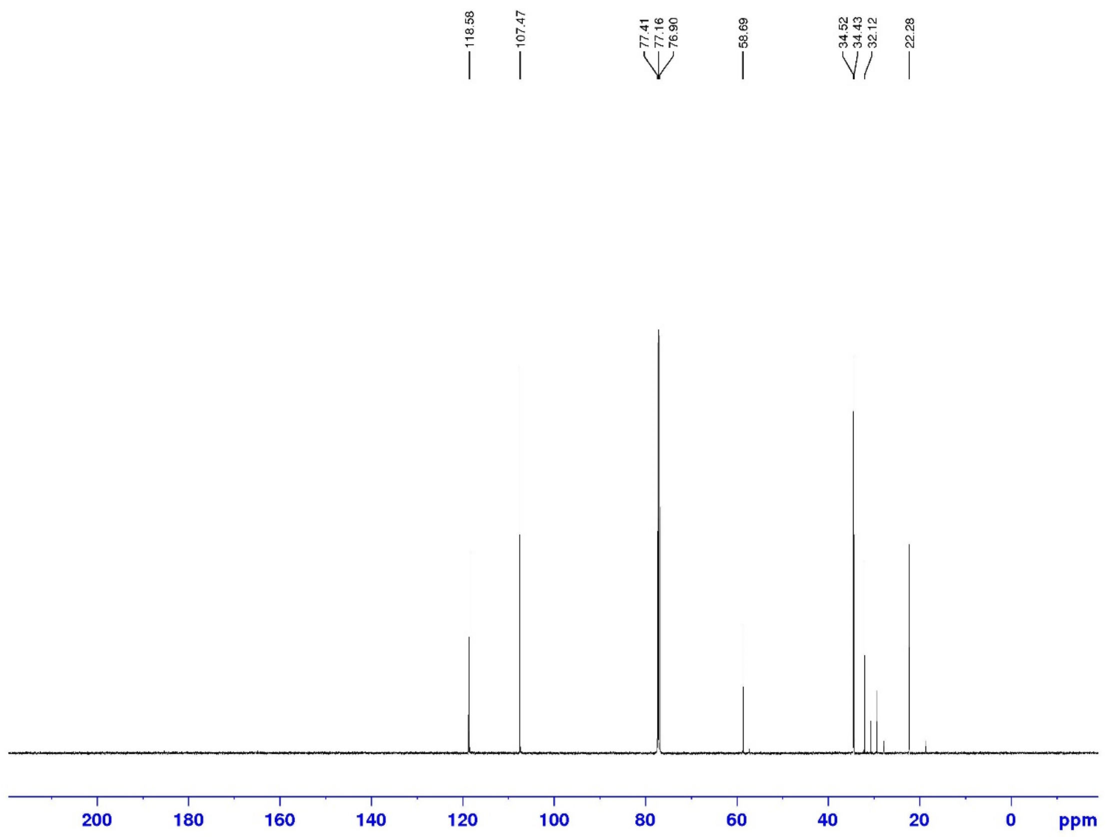
118.58
107.47
77.41
77.16
76.90
58.69
34.52
34.43
32.12
22.28
200
180
160
140
120
100
80
60
40
20
0
ppm

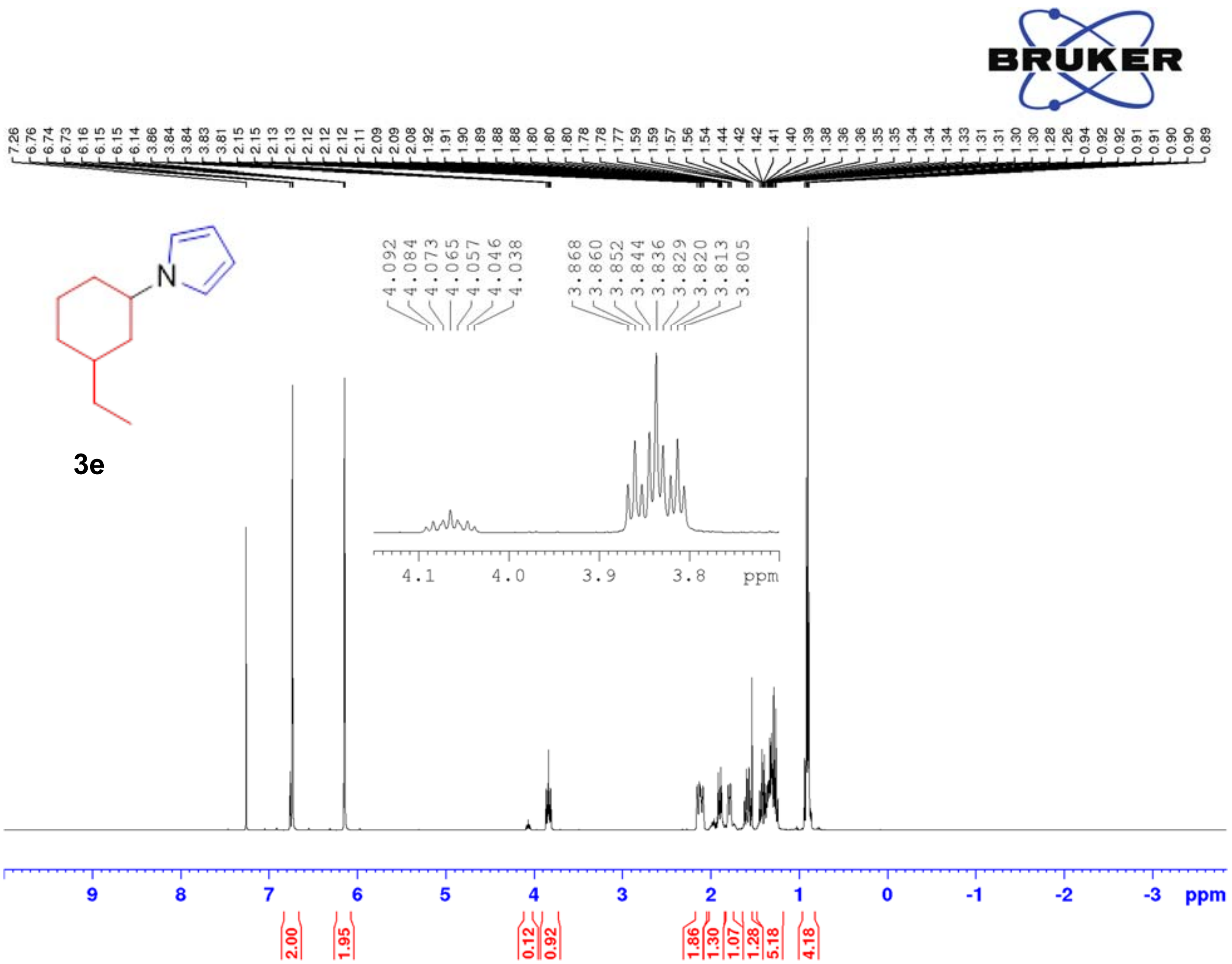
BRUKER
3e
4.092
4.084
4.073
4.065
4.057
4.046
4.038
3.868
3.860
3.852
3.844
3.836
3.829
3.820
3.813
3.805
4.1
4.0
3.9
3.8
ppm
9
8
7
6
5
4
3
2
1
0
-1
-2
-3
ppm
2.00
1.95
0.12
0.92
1.86
1.30
1.07
1.28
5.18
4.18

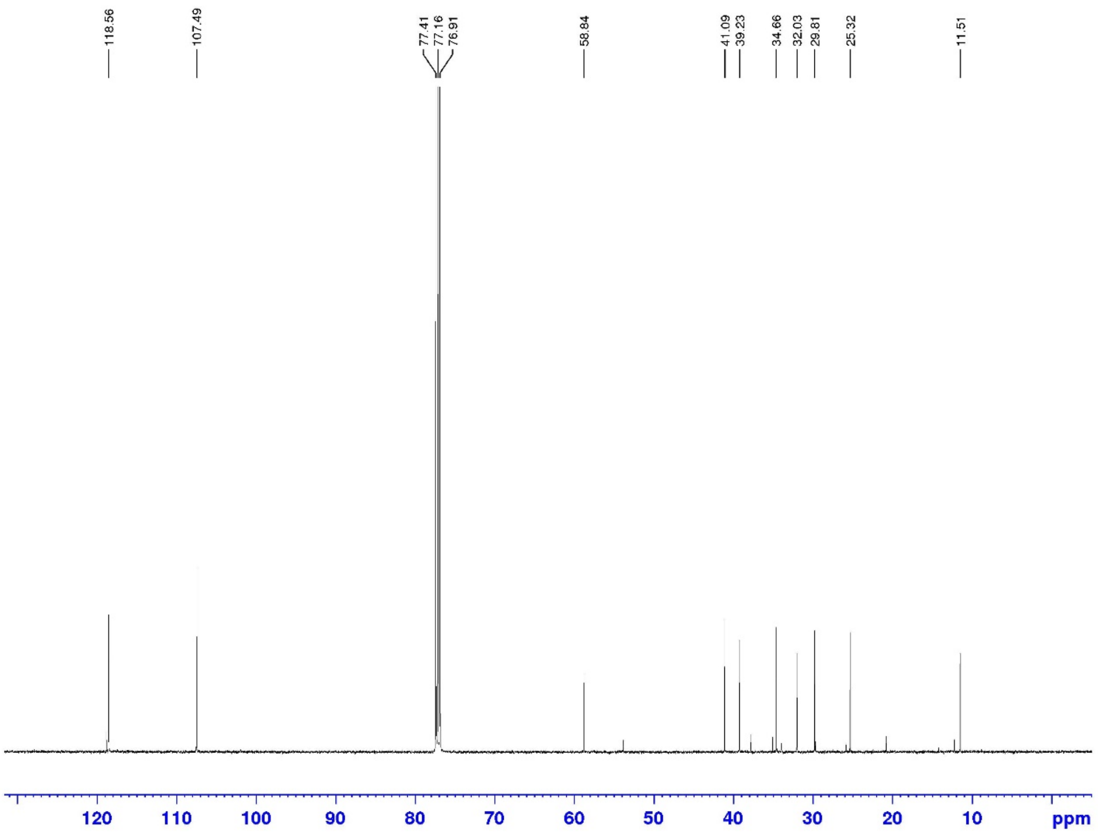

118.56
107.49
77.41
77.16
76.91
58.84
41.09
39.23
34.66
32.03
29.81
25.32
11.51
120
110
100
90
80
70
60
50
40
30
20
10
ppm

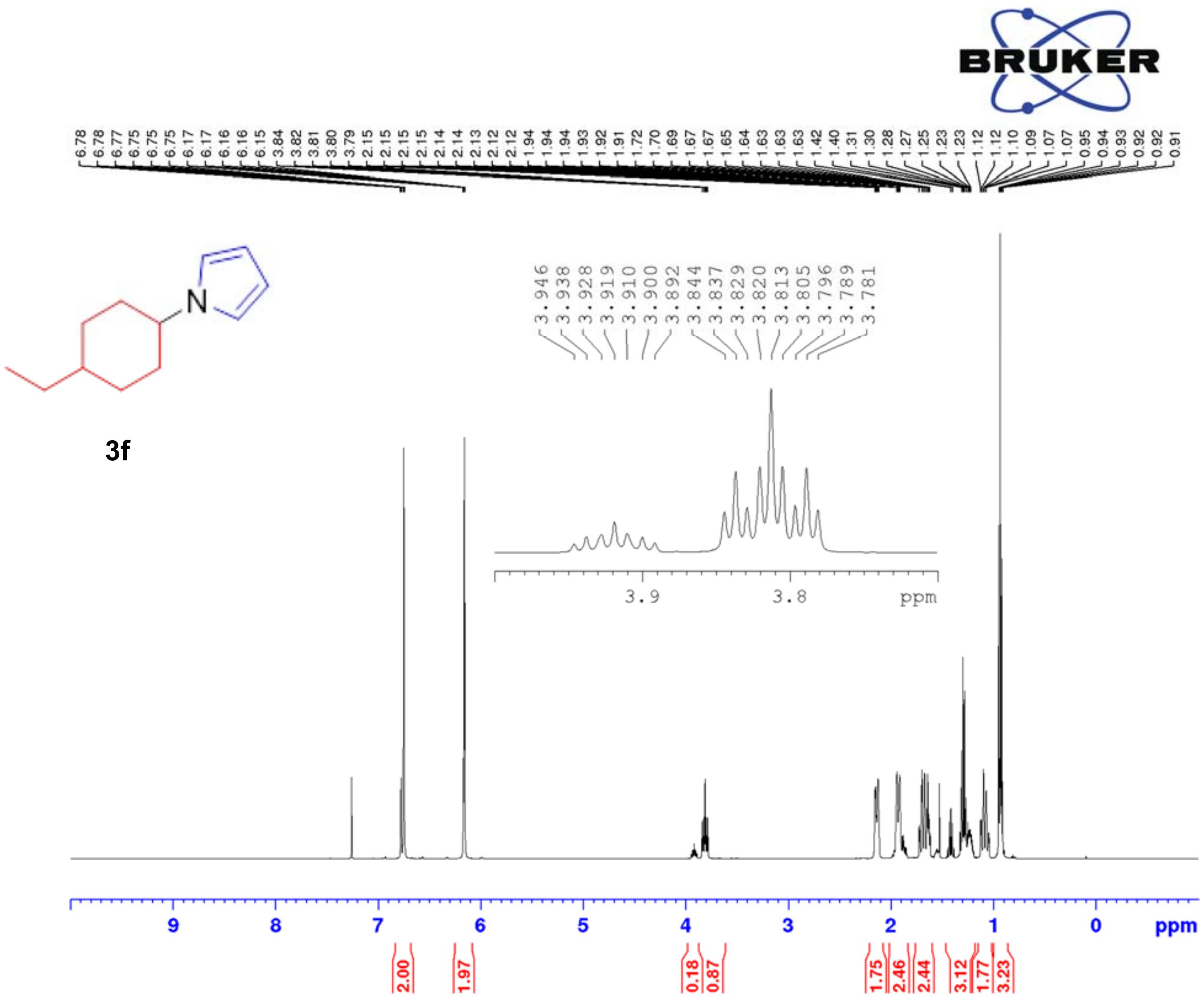
3f
6.78 6.78 6.77 6.75 6.75 6.75 6.17 6.17 6.17 6.16 6.16 6.15 3.84 3.82 3.81 3.80 3.79 2.15 2.15 2.15 2.15 2.14 2.14 2.13 2.12 2.12 1.94 1.94 1.94 1.93 1.92 1.91 1.72 1.70 1.69 1.67 1.67 1.65 1.64 1.63 1.63 1.63 1.42 1.40 1.31 1.30 1.28 1.27 1.25 1.23 1.23 1.12 1.12 1.10 1.09 1.07 1.07 0.95 0.94 0.93 0.92 0.92 0.91
3.946 3.938 3.928 3.919 3.910 3.900 3.892 3.844 3.837 3.829 3.820 3.813 3.805 3.796 3.789 3.781
3.9
3.8
ppm
9 8 7 6 5 4 3 2 1 0 ppm
2.00
1.97
0.18
0.87
1.75
2.46
2.44
3.12
1.77
3.23

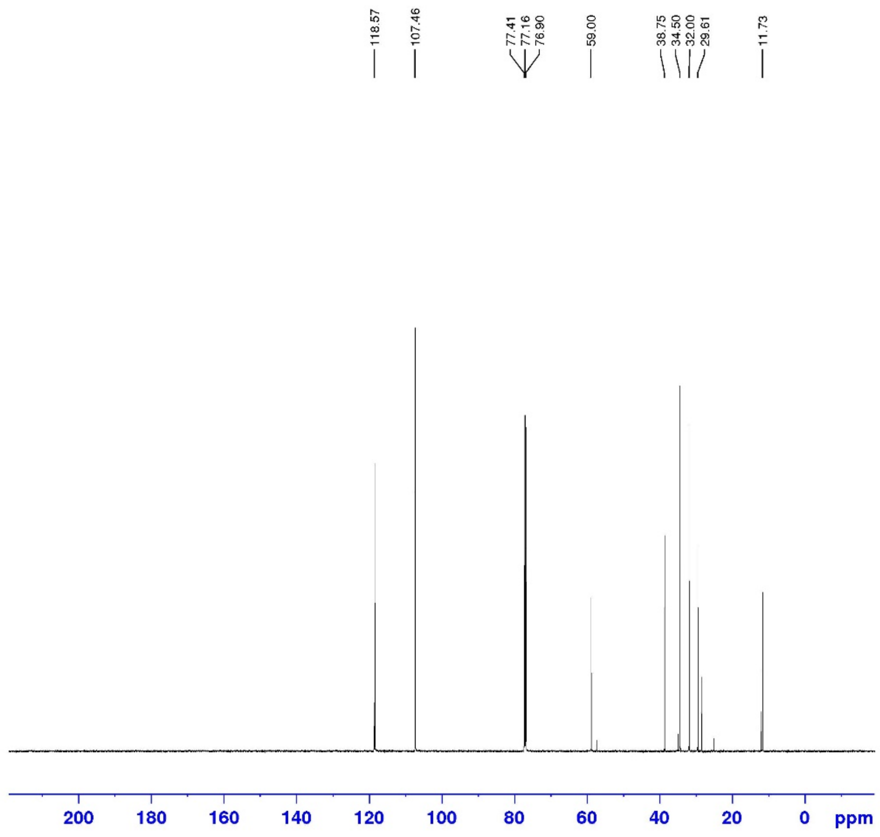
118.57
107.46
77.41
77.16
76.90
59.00
38.75
34.50
32.00
29.61
11.73
200
180
160
140
120
100
80
60
40
20
0
ppm

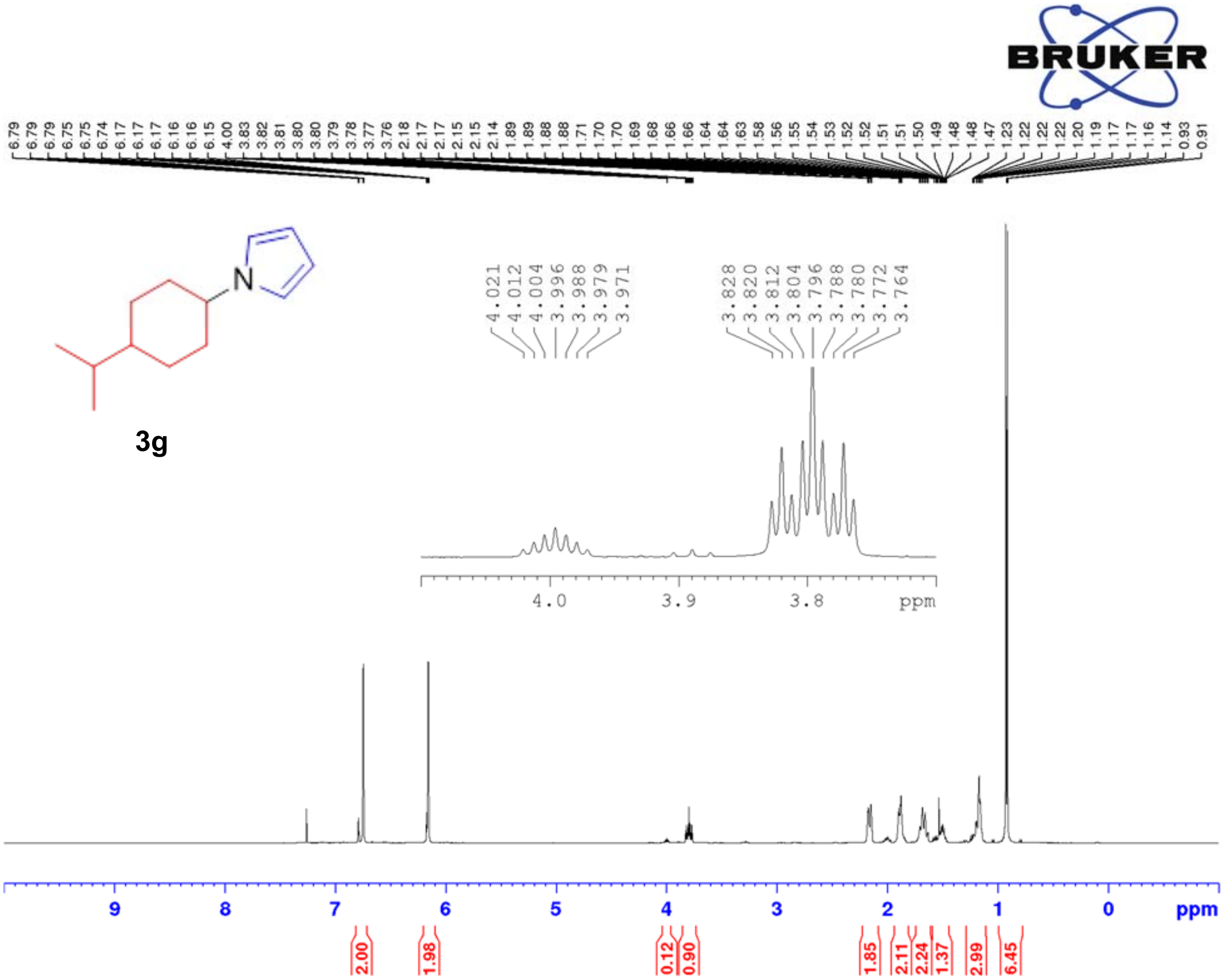
6.79 6.79 6.79 6.75 6.75 6.74 6.17 6.17 6.17 6.16 6.16 6.15 4.00 3.83 3.82 3.81 3.80 3.80 3.79 3.78 3.77 3.76 2.18 2.17 2.17 2.15 2.15 2.14 1.89 1.89 1.88 1.88 1.71 1.70 1.70 1.69 1.68 1.66 1.64 1.64 1.63 1.58 1.56 1.55 1.54 1.53 1.52 1.52 1.51 1.51 1.50 1.49 1.48 1.48 1.47 1.23 1.22 1.22 1.22 1.20 1.19 1.17 1.17 1.16 1.14 0.93 0.91
N
3g
4.021 4.012 4.004 3.996 3.988 3.979 3.971
3.828 3.820 3.812 3.804 3.796 3.788 3.780 3.772 3.764
4.0 3.9 3.8 ppm
9 8 7 6 5 4 3 2 1 0 ppm
2.00 1.98 0.12 0.90 1.85 2.11 2.24 1.37 2.99 6.45

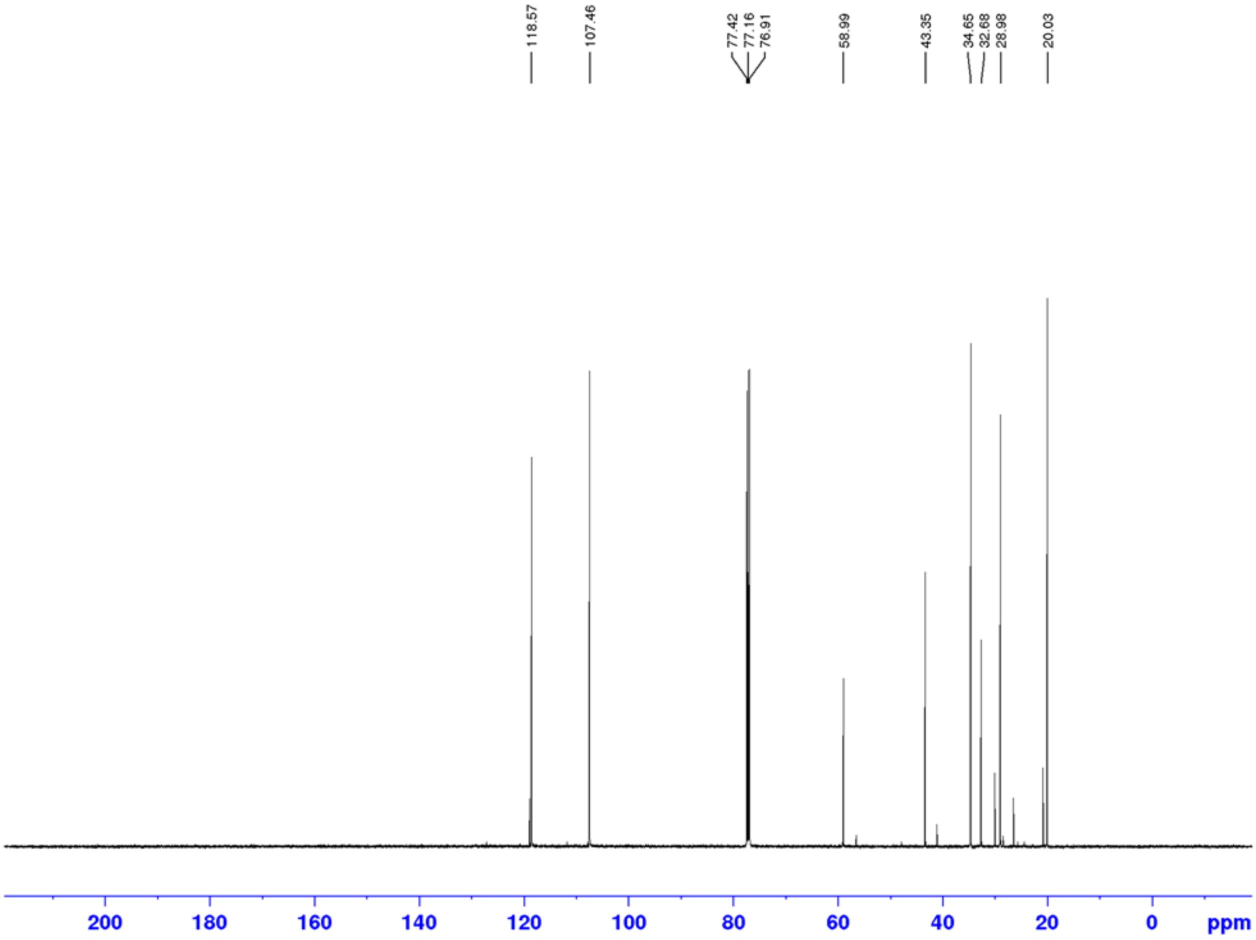
118.57
107.46
77.42
77.16
76.91
58.99
43.35
34.65
32.68
28.98
20.03
200
180
160
140
120
100
80
60
40
20
0
ppm

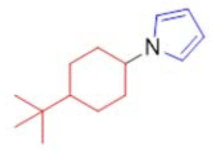

3h

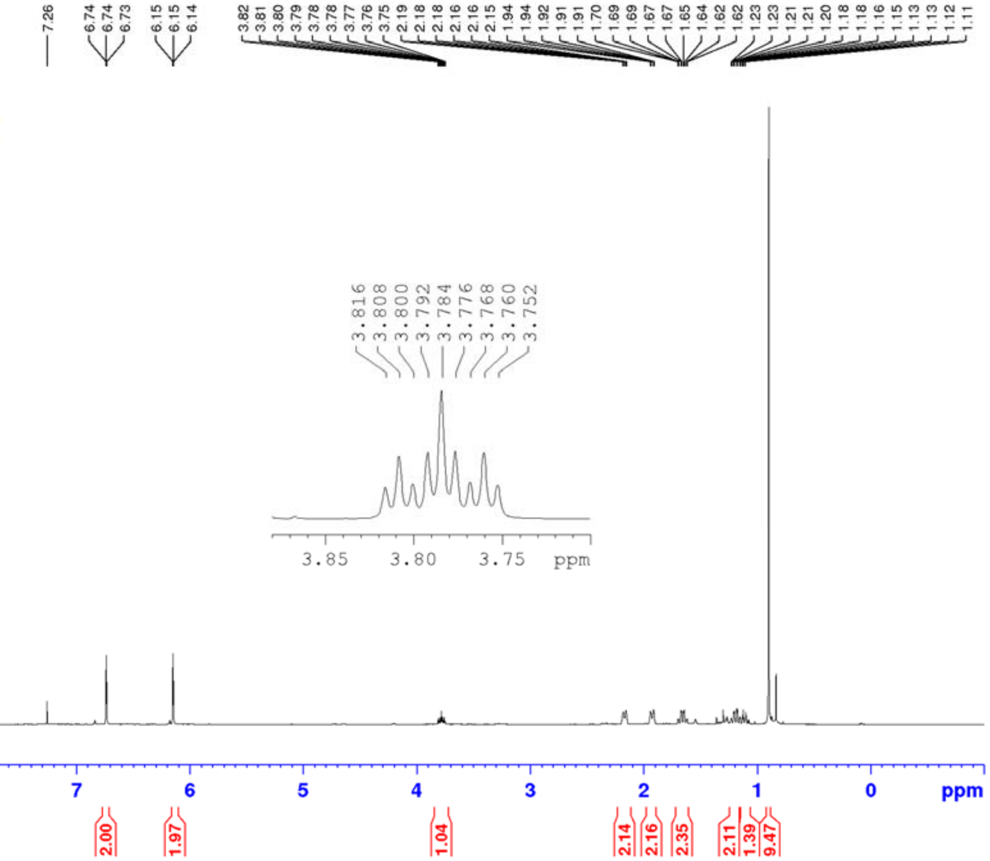

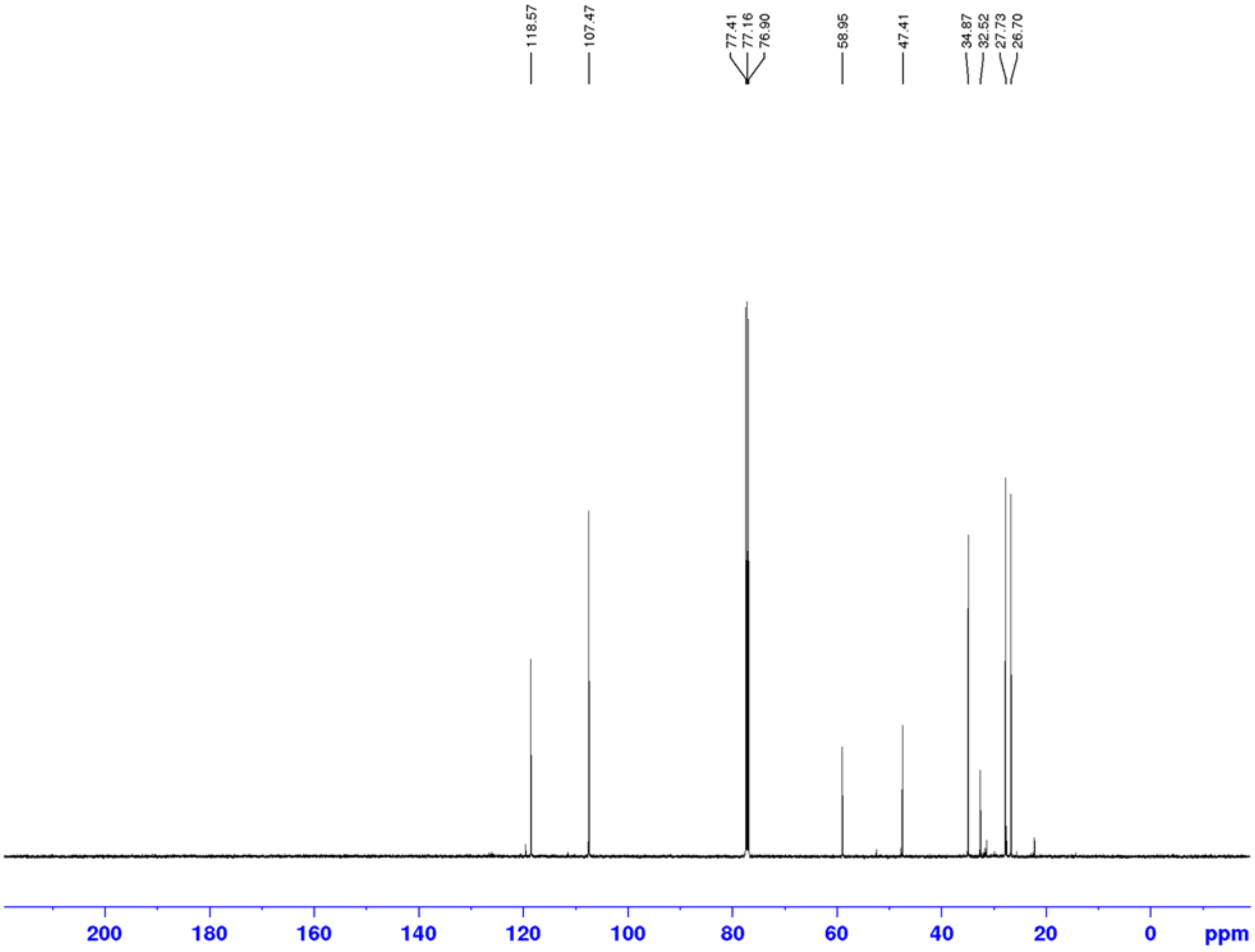
118.57
107.47
77.41
77.16
76.90
58.95
47.41
34.87
32.52
27.73
26.70
200
180
160
140
120
100
80
60
40
20
0
ppm

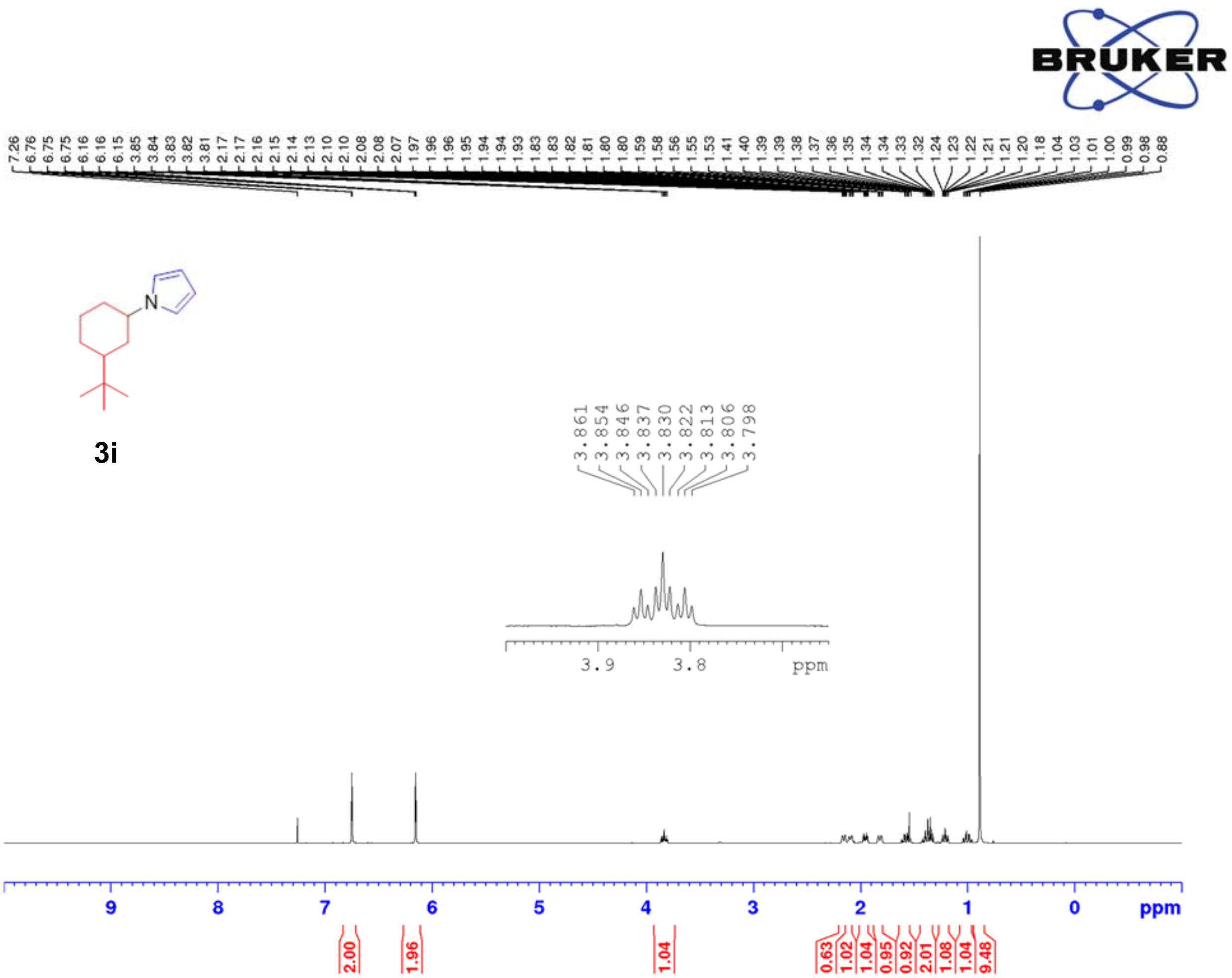
BRUKER
7.26 6.76 6.75 6.16 6.16 6.15 3.85 3.84 3.83 3.82 3.81 2.17 2.17 2.16 2.15 2.14 2.13 2.10 2.10 2.08 2.08 2.07 1.97 1.96 1.96 1.95 1.94 1.94 1.93 1.83 1.83 1.82 1.81 1.80 1.80 1.59 1.58 1.56 1.55 1.53 1.41 1.40 1.39 1.39 1.38 1.37 1.36 1.35 1.34 1.34 1.33 1.32 1.24 1.23 1.22 1.21 1.21 1.20 1.18 1.04 1.03 1.01 1.00 0.99 0.98 0.88
N
3.861 3.854 3.846 3.837 3.830 3.822 3.813 3.806 3.798
3.9 3.8 ppm
9 8 7 6 5 4 3 2 1 0 ppm
2.00 1.96 1.04 0.63 1.02 1.04 0.95 0.92 2.01 1.08 1.04 9.48

3i

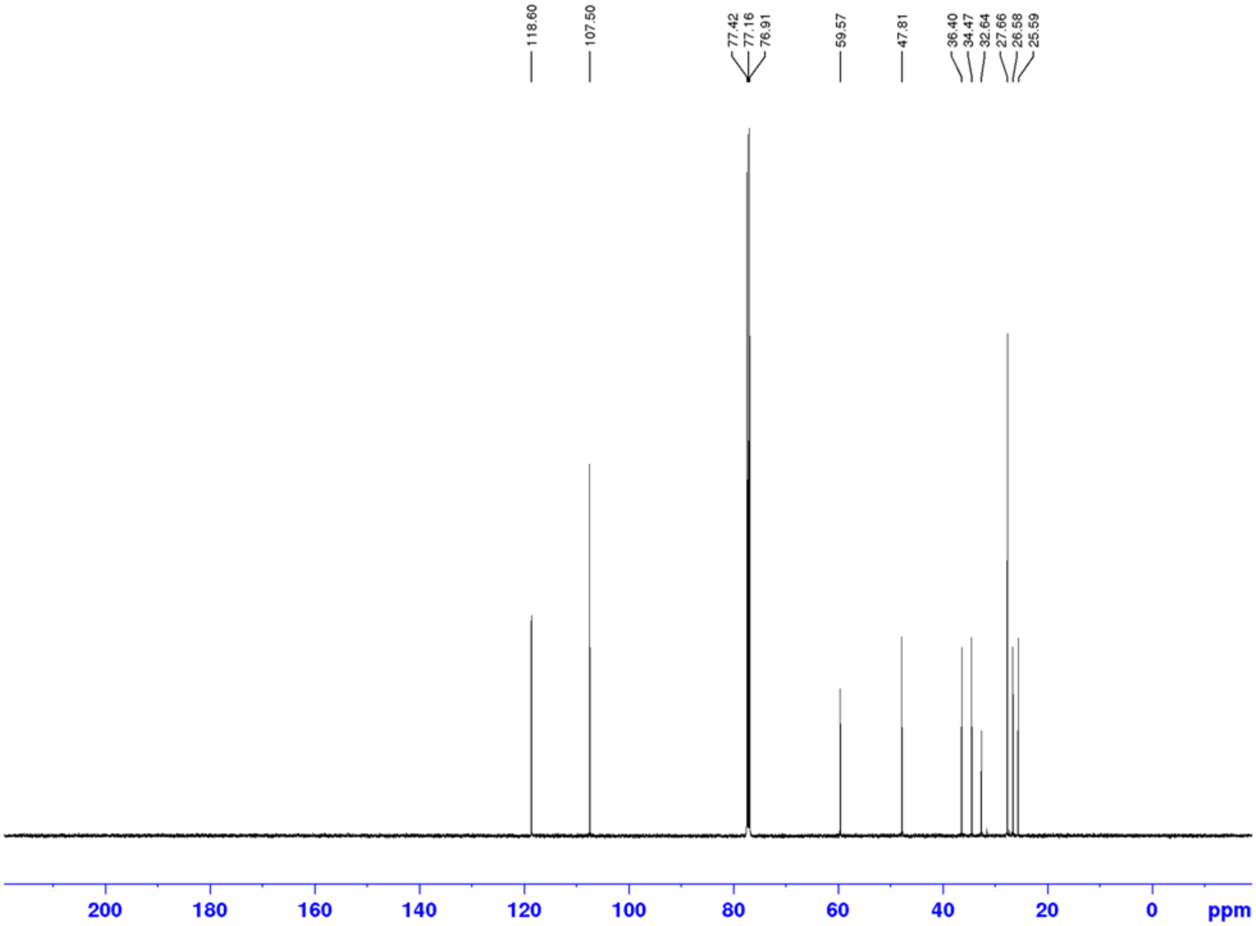
118.60
107.50
77.42
77.16
76.91
59.57
47.81
36.40
34.47
32.64
27.66
26.58
25.59
200
180
160
140
120
100
80
60
40
20
0
ppm

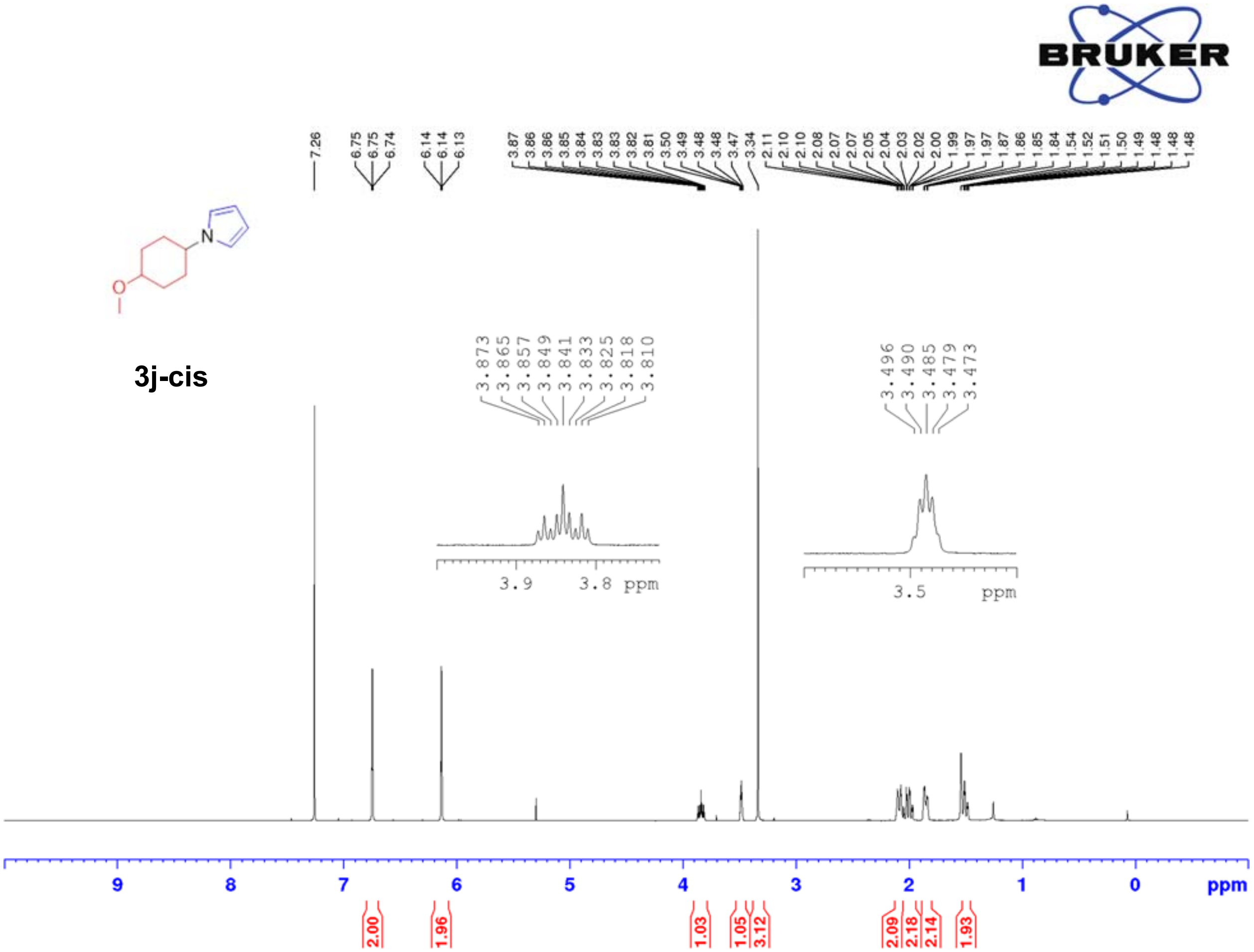
3j-cis
BRUKER
3.9 3.8 ppm
3.5 ppm
9 8 7 6 5 4 3 2 1 0 ppm
2.00 1.96 1.03 1.05 3.12 2.09 2.18 2.14 1.93

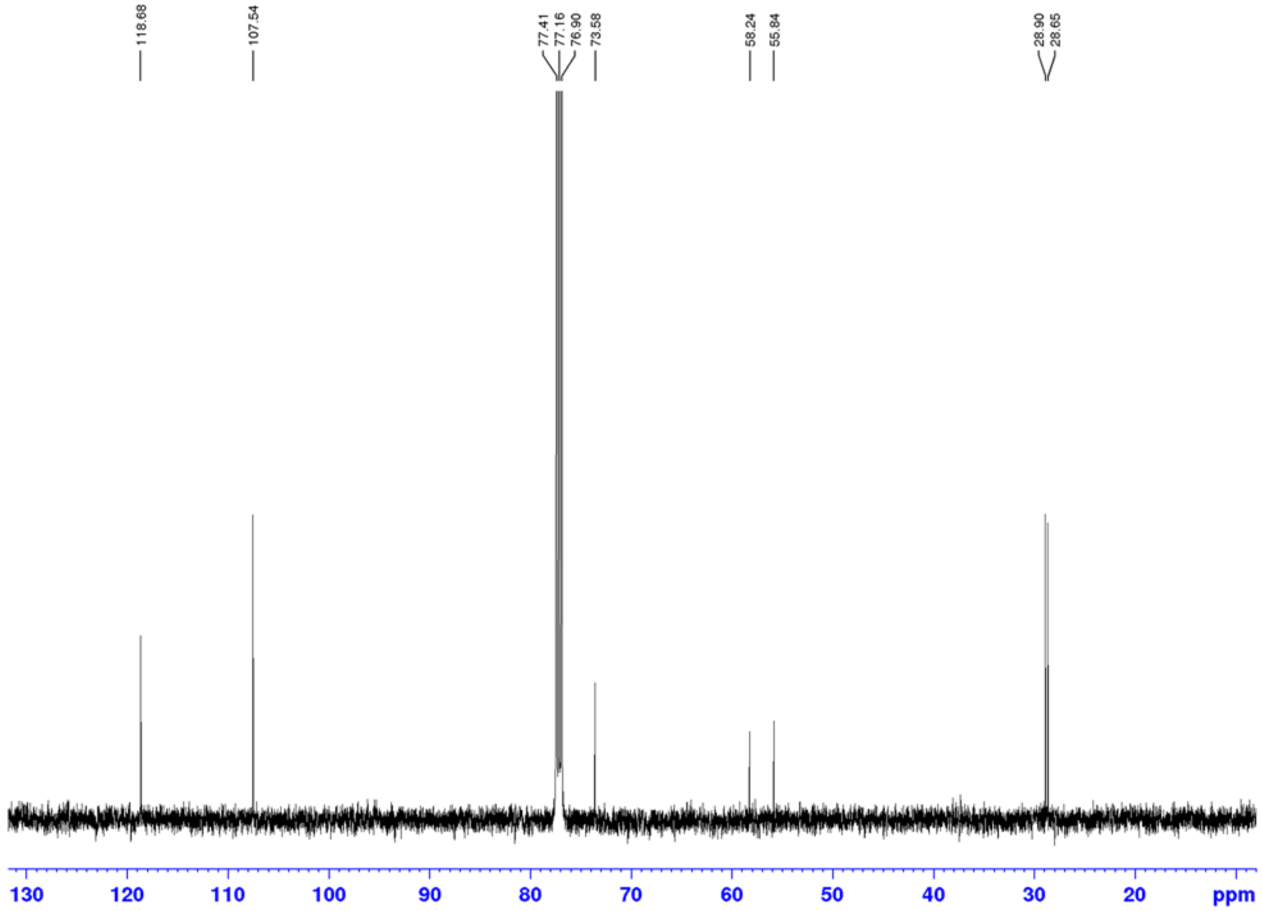
118.68
107.54
77.41
77.16
76.90
73.58
58.24
55.84
28.90
28.65
130
120
110
100
90
80
70
60
50
40
30
20
ppm

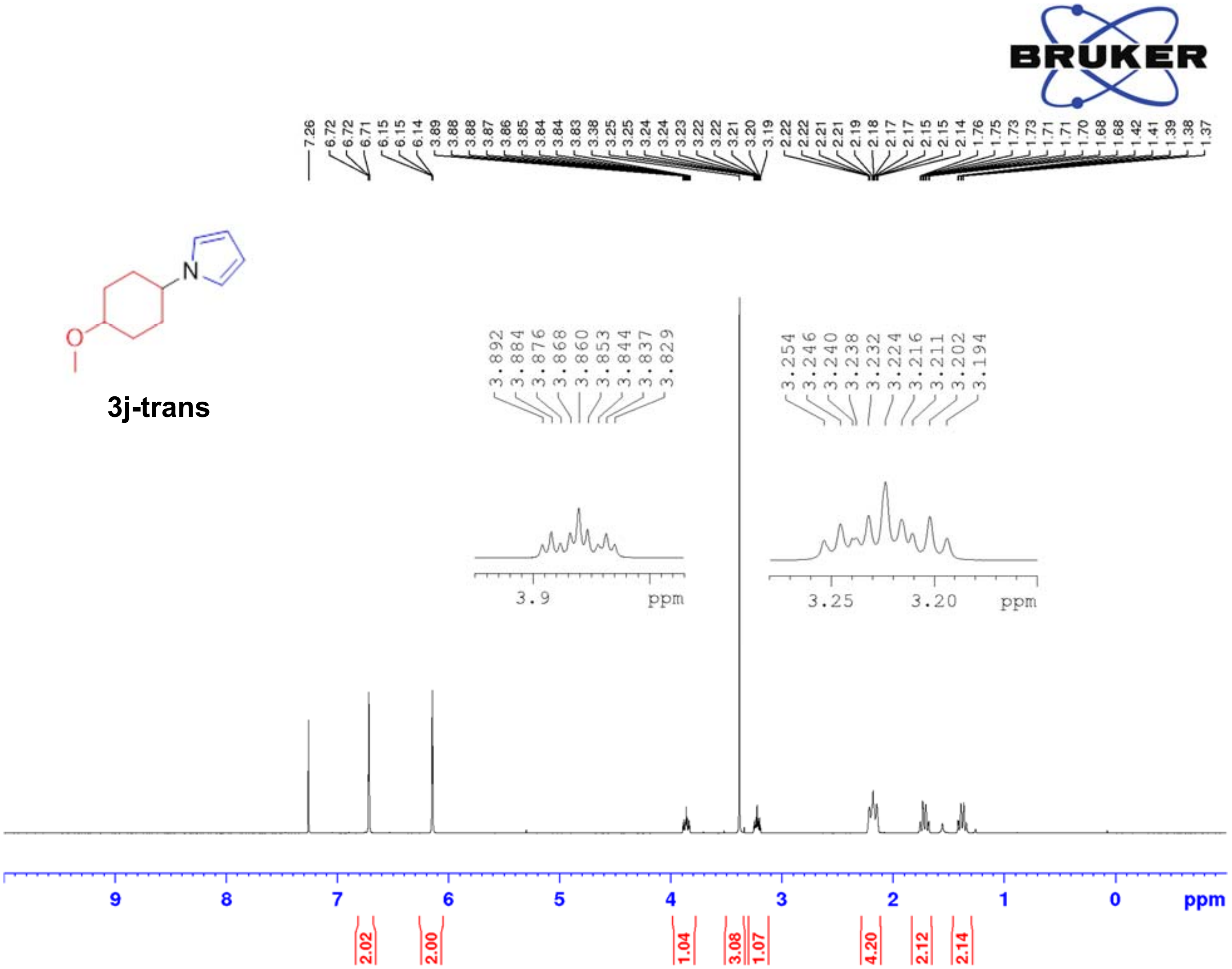

BRUKER
7.26
6.72
6.72
6.71
6.15
6.15
6.14
3.89
3.88
3.88
3.87
3.86
3.85
3.84
3.84
3.83
3.38
3.25
3.25
3.24
3.24
3.23
3.22
3.22
3.21
3.20
3.19
2.22
2.22
2.21
2.21
2.19
2.18
2.17
2.17
2.15
2.15
2.14
1.76
1.75
1.73
1.73
1.71
1.71
1.70
1.68
1.68
1.42
1.41
1.39
1.38
1.37
N
O
3.892
3.884
3.876
3.868
3.860
3.853
3.844
3.837
3.829
3.9
ppm
3.254
3.246
3.240
3.238
3.232
3.224
3.216
3.211
3.202
3.194
3.25
3.20
ppm
9
8
7
6
5
4
3
2
1
0
ppm
2.02
2.00
1.04
3.08
1.07
4.20
2.12
2.14

3j-trans

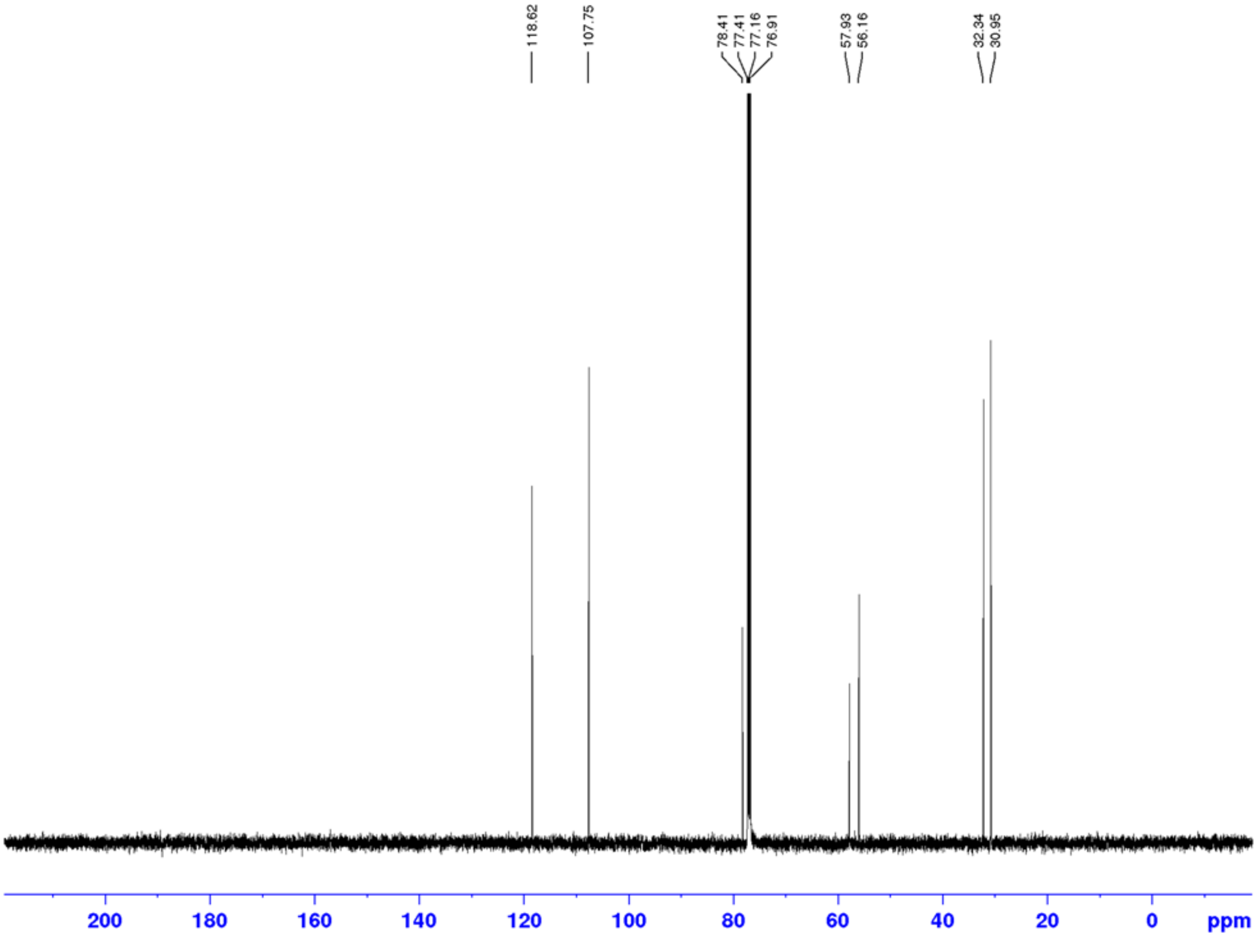
118.62
107.75
78.41
77.41
77.16
76.91
57.93
56.16
32.34
30.95
200
180
160
140
120
100
80
60
40
20
0
ppm

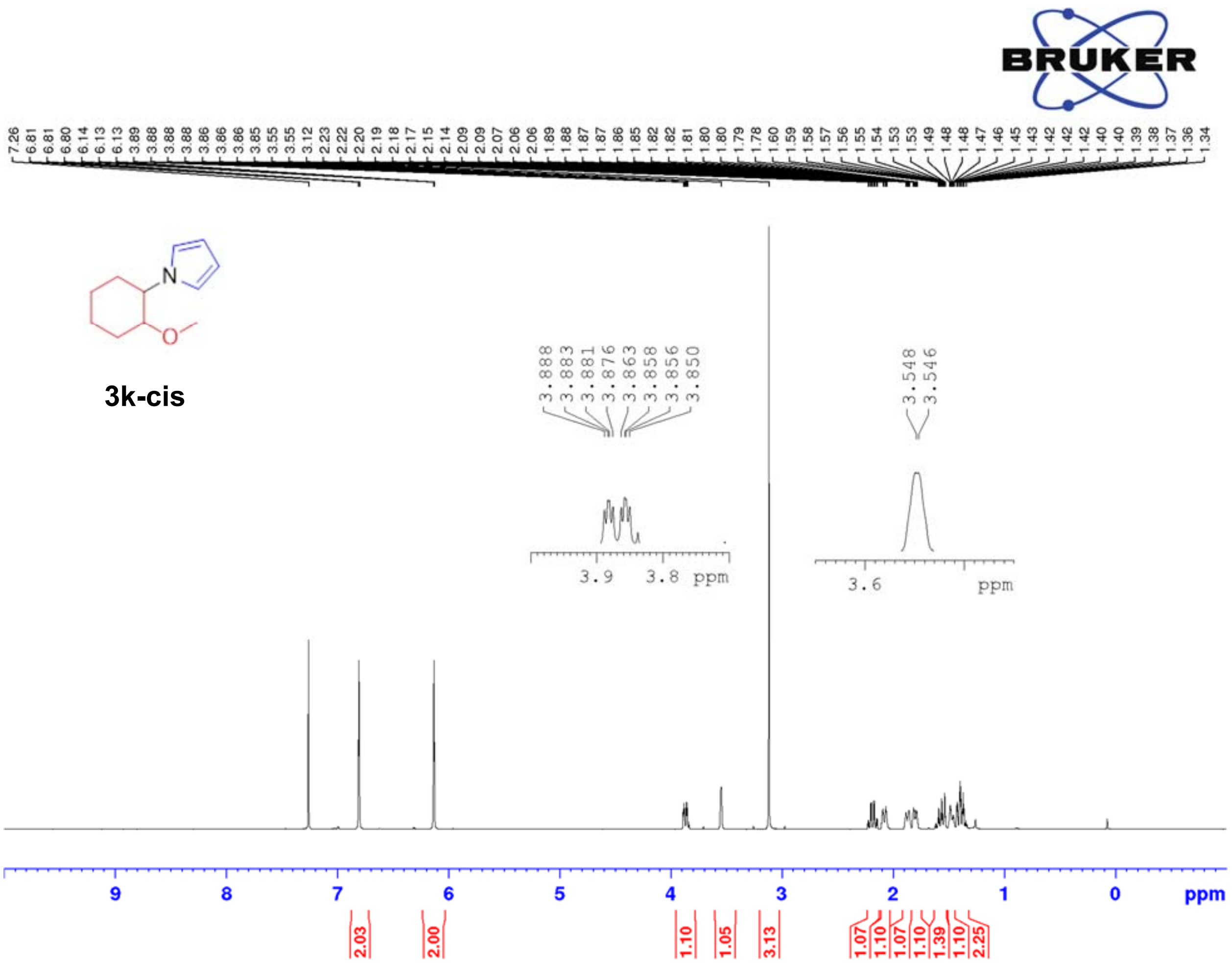
3k-cis
BRUKER
3.888
3.883
3.881
3.876
3.863
3.858
3.856
3.850
3.548
3.546
ppm
2.03
2.00
1.10
1.05
3.13
1.07
1.10
1.07
1.10
1.39
1.10
2.25

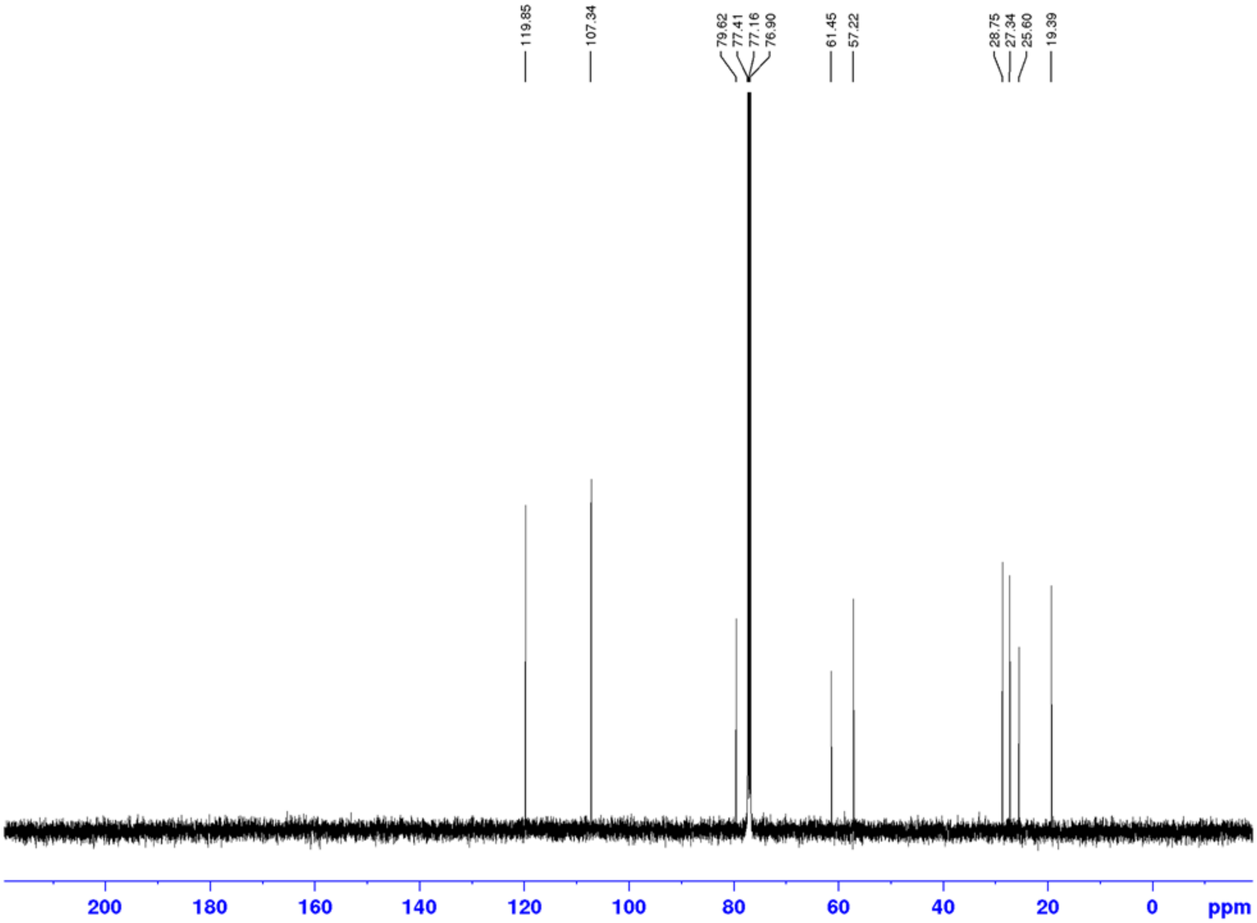

119.85
107.34
79.62
77.41
77.16
76.90
61.45
57.22
28.75
27.34
25.60
19.39
200
180
160
140
120
100
80
60
40
20
0
ppm

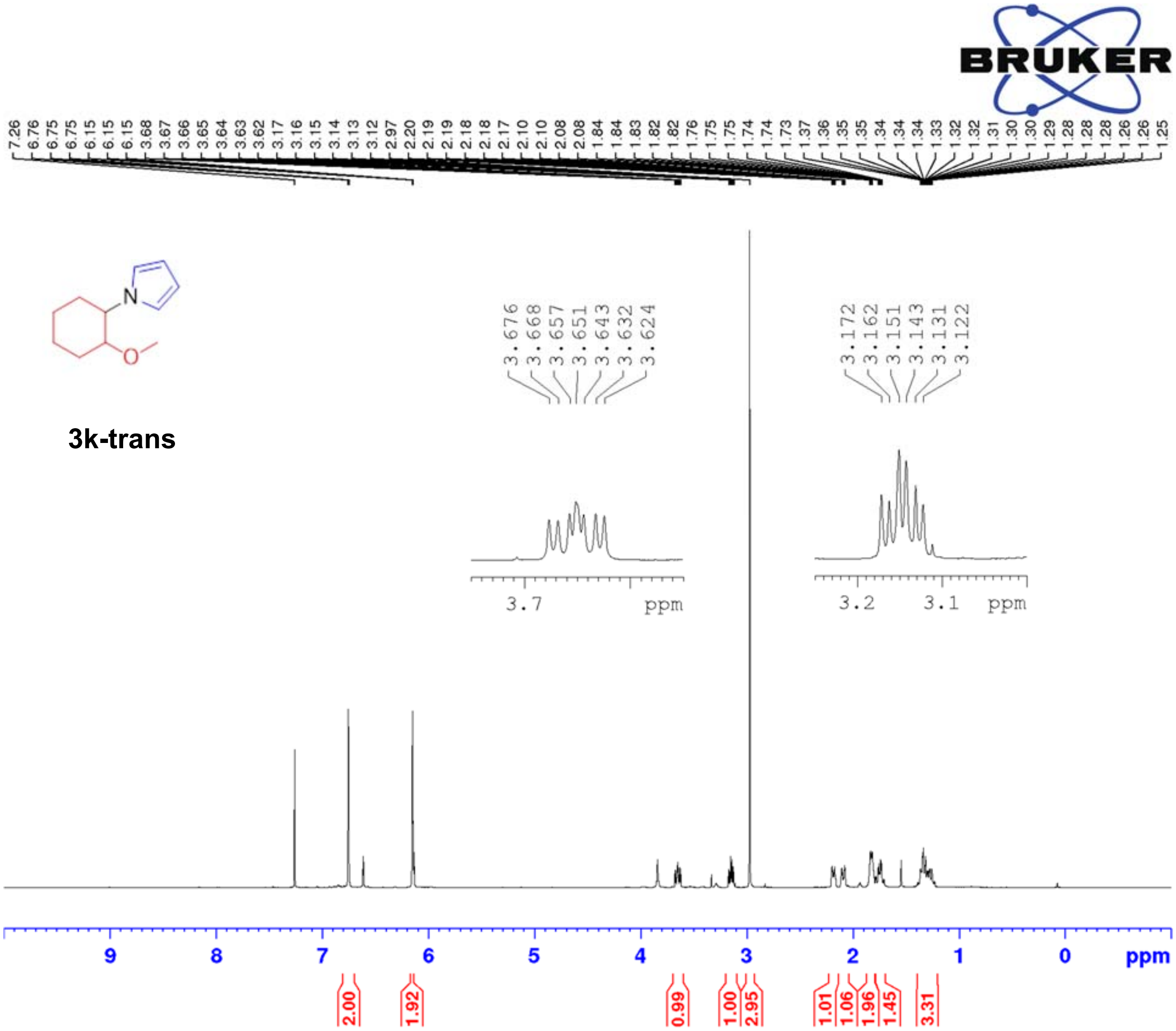

7.26 6.76 6.75 6.75 6.15 6.15 6.15 6.15 3.68 3.67 3.66 3.65 3.64 3.63 3.62 3.17 3.16 3.15 3.14 3.13 3.12 2.97 2.20 2.19 2.19 2.18 2.18 2.17 2.10 2.10 2.08 2.08 1.84 1.84 1.83 1.83 1.82 1.76 1.75 1.75 1.74 1.74 1.73 1.37 1.36 1.35 1.35 1.34 1.34 1.34 1.33 1.32 1.32 1.31 1.30 1.30 1.29 1.28 1.28 1.26 1.26 1.25
3.676 3.668 3.657 3.651 3.643 3.632 3.624
3.7 ppm
3.172 3.162 3.151 3.143 3.131 3.122
3.2 3.1 ppm
9 8 7 6 5 4 3 2 1 0 ppm
2.00 1.92 0.99 1.00 2.95 1.01 1.06 1.96 1.45 3.31

3k-trans

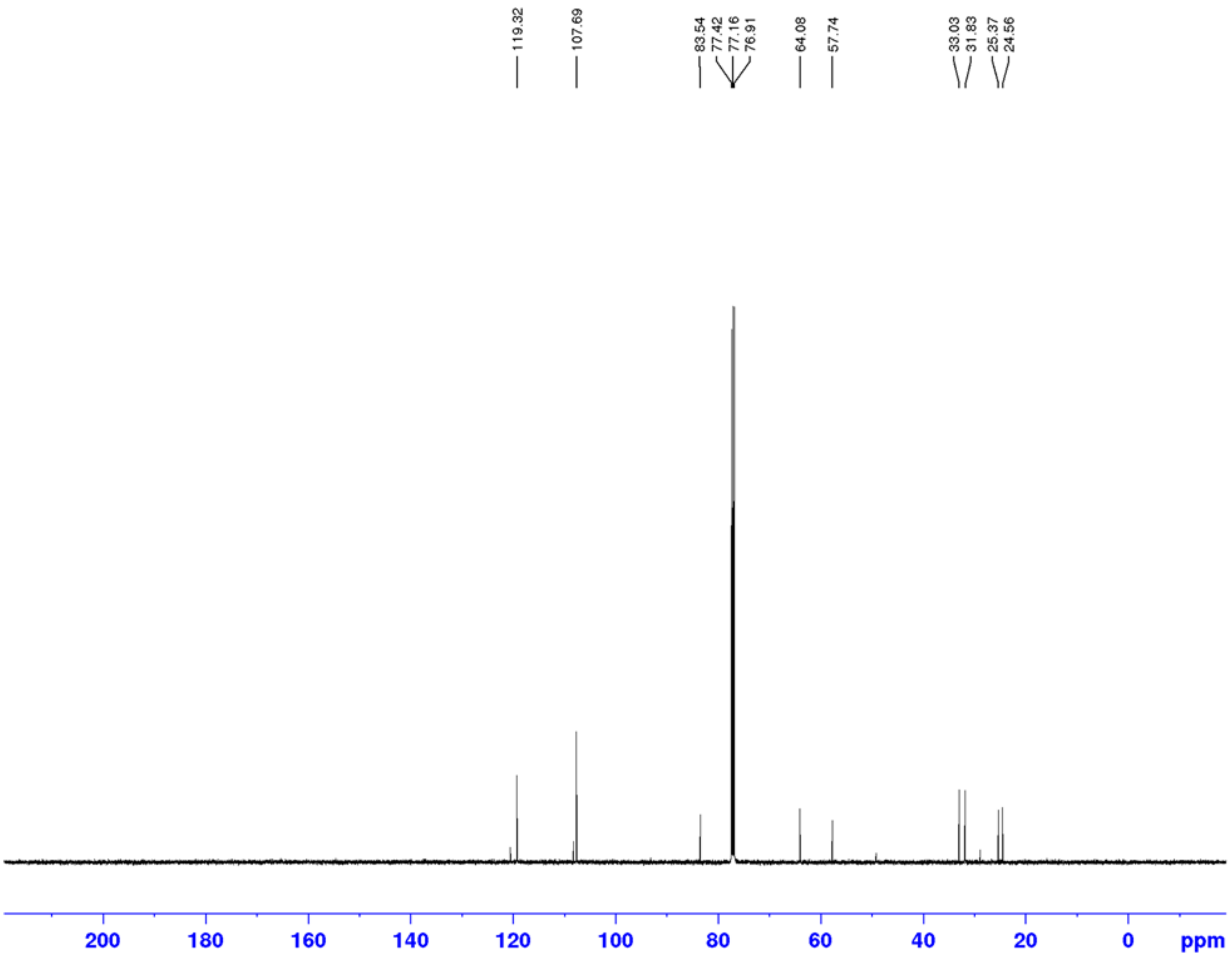
119.32
107.69
83.54
77.42
77.16
76.91
64.08
57.74
33.03
31.83
25.37
24.56
200
180
160
140
120
100
80
60
40
20
0
ppm

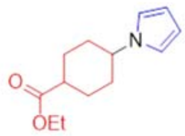

3l-cis

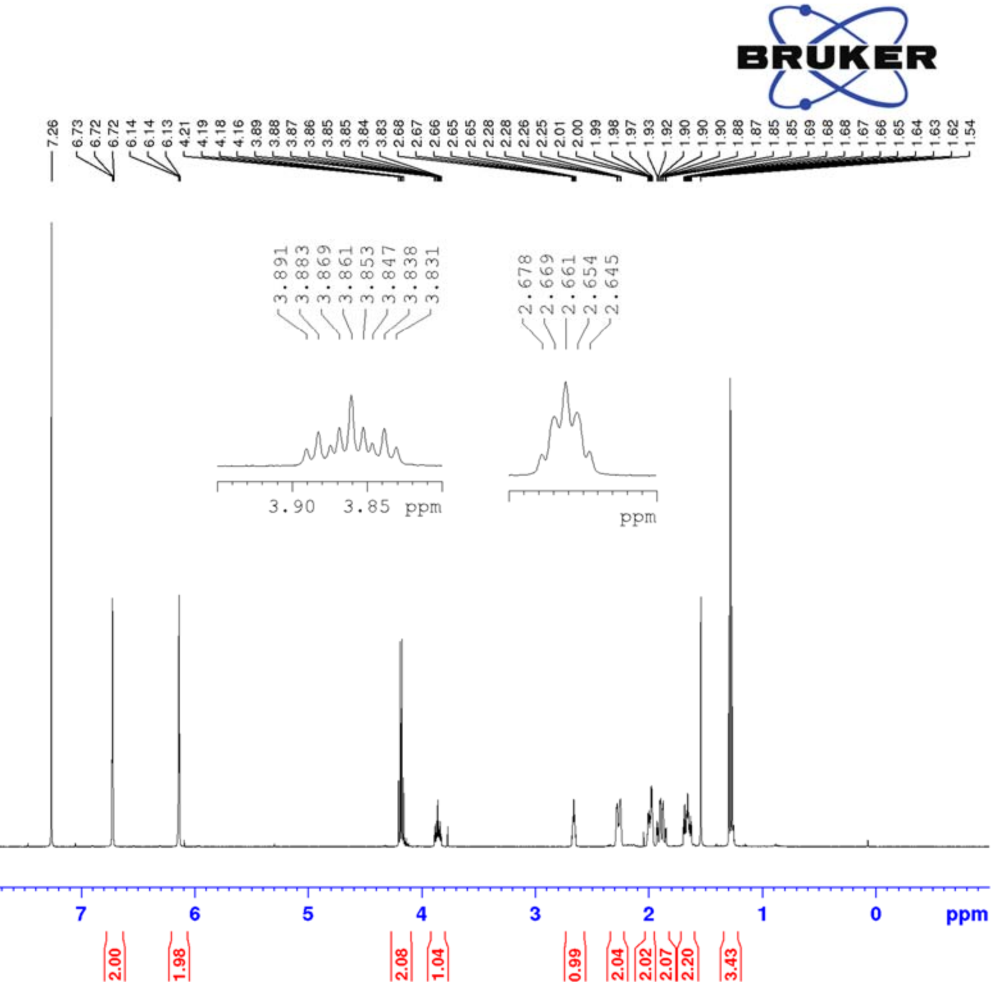

BRUKER
7.26
6.73
6.72
6.72
6.14
6.14
6.13
3.891
3.883
3.869
3.861
3.853
3.847
3.838
3.831
3.90 3.85 ppm
2.678
2.669
2.661
2.654
2.645
ppm
9 8 7 6 5 4 3 2 1 0 ppm
2.00
1.98
2.08
1.04
0.99
2.04
2.02
2.07
2.20
3.43

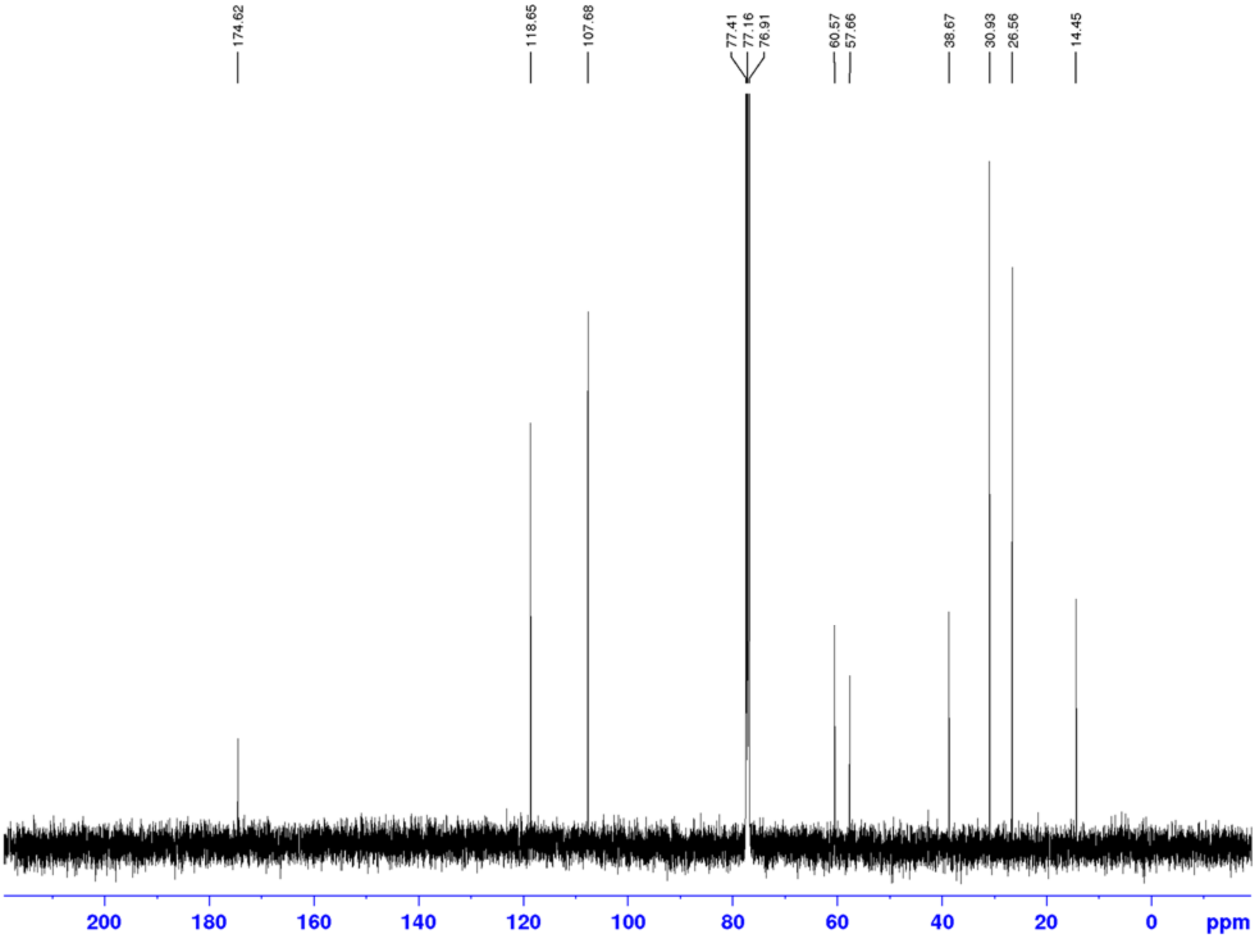
174.62
118.65
107.68
77.41
77.16
76.91
60.57
57.66
38.67
30.93
26.56
14.45
200
180
160
140
120
100
80
60
40
20
0
ppm

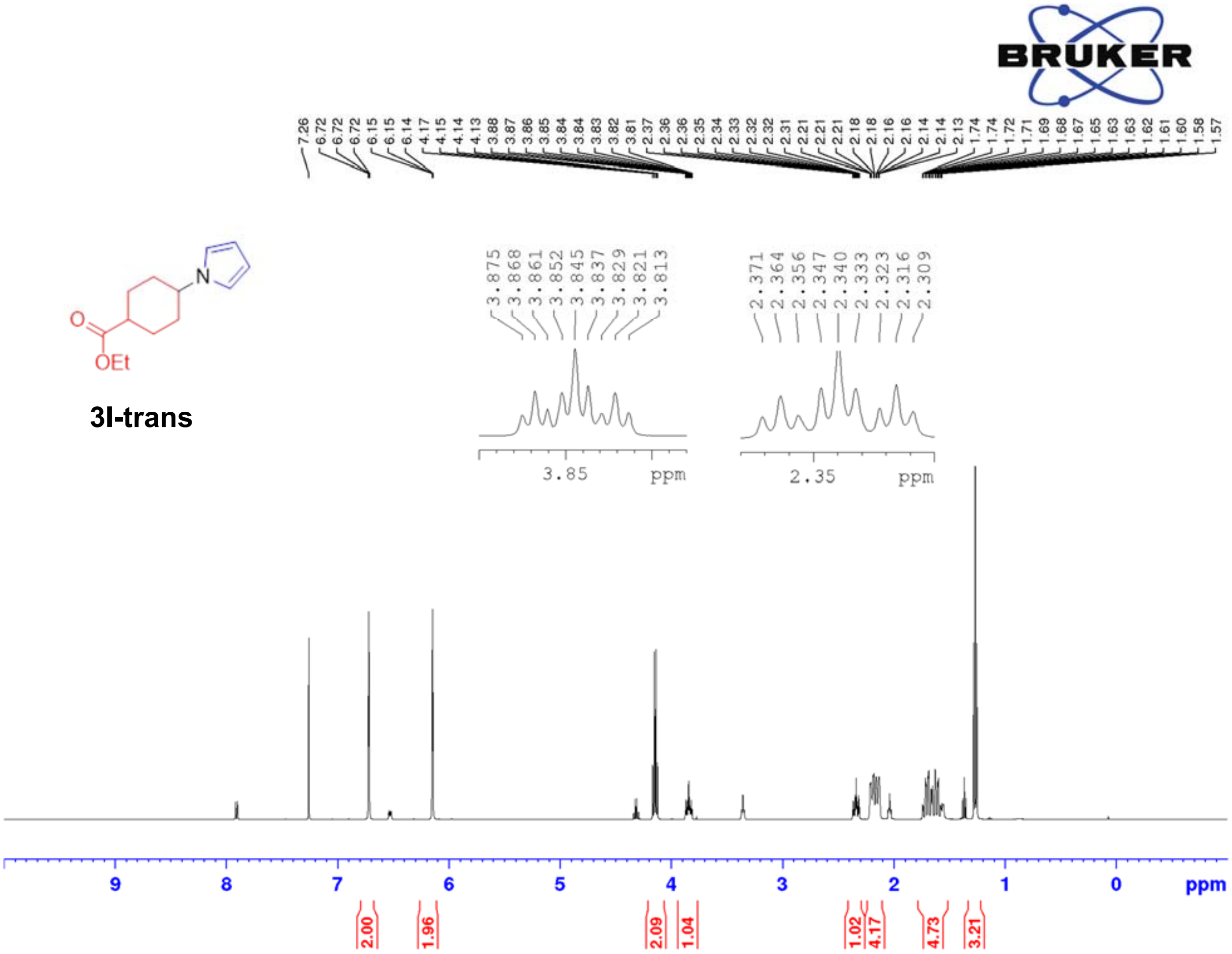
BRUKER
7.26 6.72 6.72 6.72 6.15 6.15 6.14 4.17 4.15 4.14 4.13 3.88 3.87 3.86 3.85 3.84 3.84 3.83 3.82 3.81 2.37 2.36 2.36 2.35 2.34 2.33 2.32 2.32 2.31 2.21 2.21 2.21 2.18 2.18 2.16 2.16 2.14 2.14 2.13 1.74 1.74 1.72 1.71 1.69 1.68 1.67 1.65 1.63 1.63 1.62 1.61 1.60 1.58 1.57
3.875 3.868 3.861 3.852 3.845 3.837 3.829 3.821 3.813
3.85 ppm
2.371 2.364 2.356 2.347 2.340 2.333 2.323 2.316 2.309
2.35 ppm
9 8 7 6 5 4 3 2 1 0 ppm
2.00 1.96 2.09 1.04 1.02 4.17 4.73 3.21

3l-trans

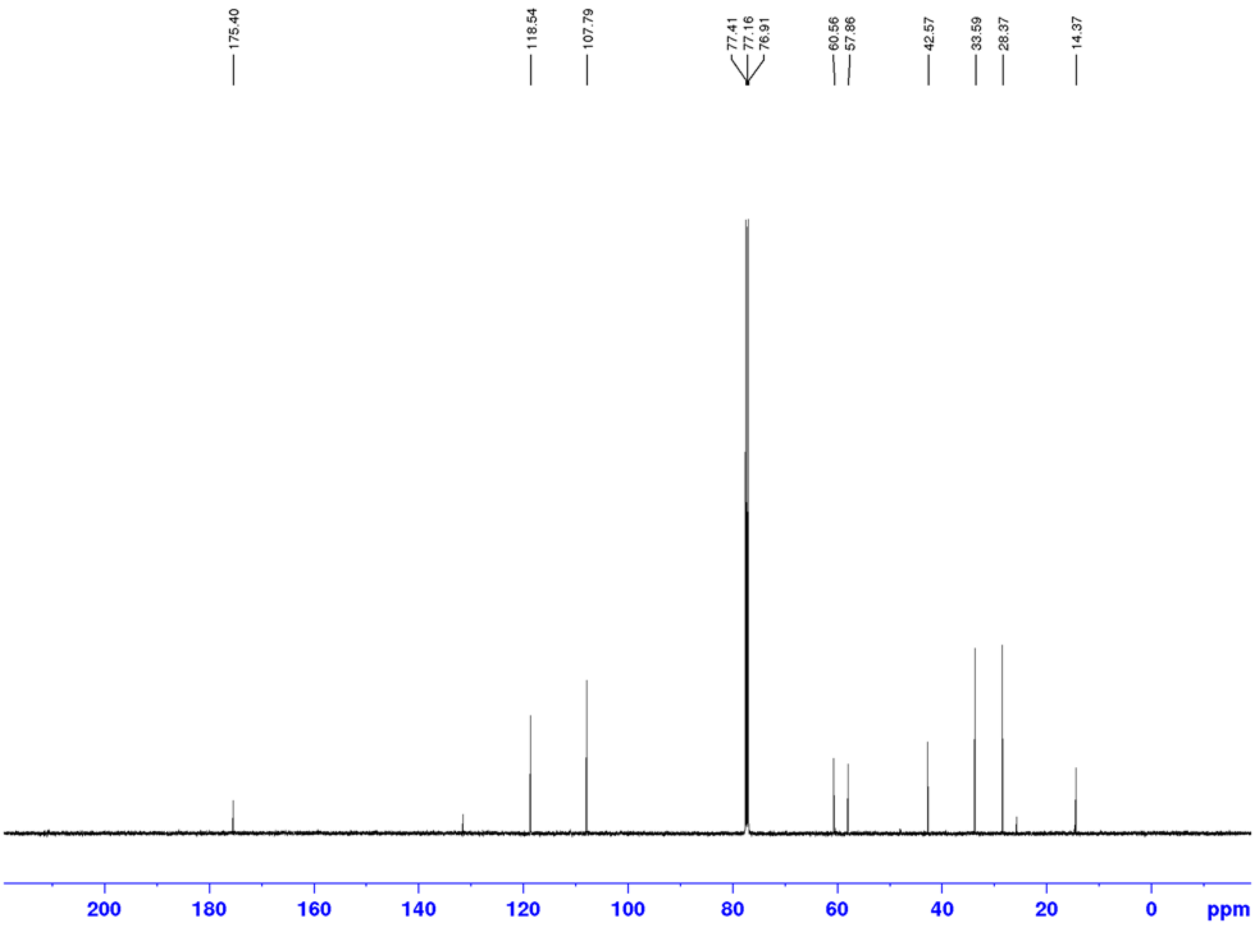
175.40
118.54
107.79
77.41
77.16
76.91
60.56
57.86
42.57
33.59
28.37
14.37
200
180
160
140
120
100
80
60
40
20
0
ppm

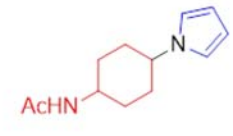

AcHN
N

3m-cis

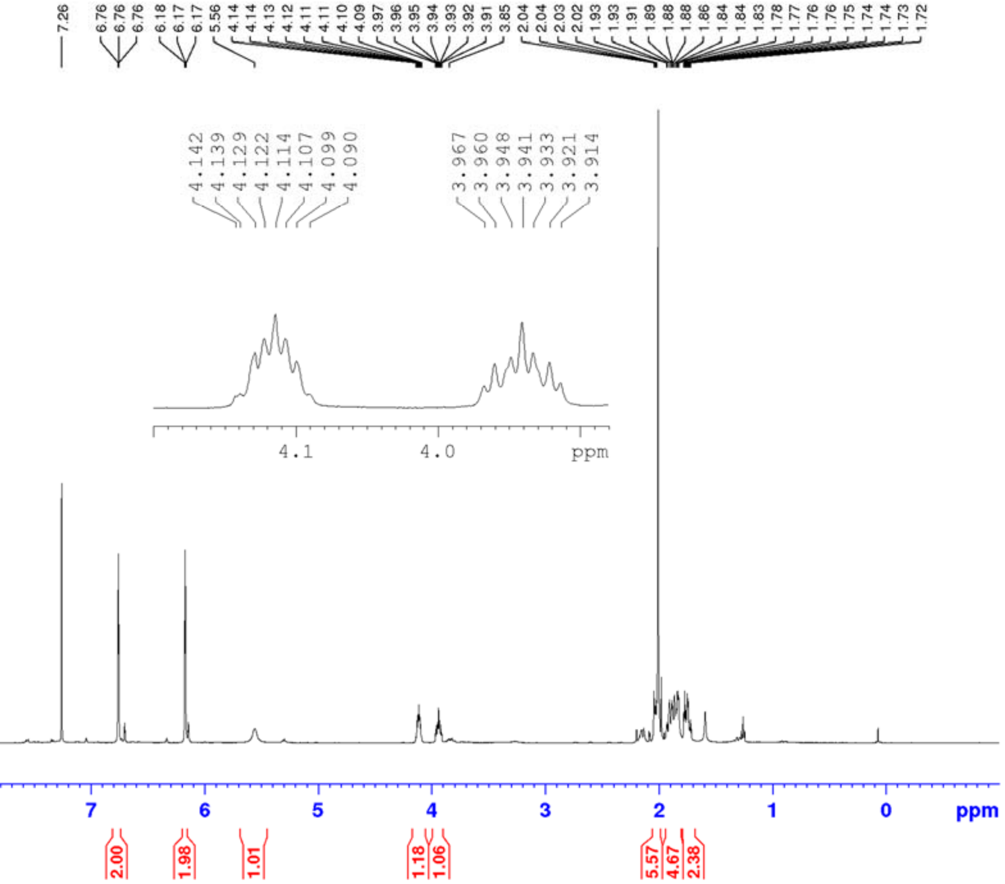

7.26
6.76
6.76
6.76
6.18
6.17
6.17
5.56
4.14
4.14
4.13
4.12
4.11
4.11
4.10
4.09
3.97
3.96
3.95
3.94
3.93
3.92
3.91
3.85
2.04
2.04
2.03
2.02
1.93
1.93
1.91
1.89
1.88
1.88
1.88
1.86
1.84
1.84
1.83
1.78
1.77
1.76
1.76
1.75
1.74
1.74
1.73
1.72
4.142
4.139
4.129
4.122
4.114
4.107
4.099
4.090
3.967
3.960
3.948
3.941
3.933
3.921
3.914
4.1
4.0
ppm
9
8
7
6
5
4
3
2
1
0
ppm
2.00
1.98
1.01
1.18
1.06
5.57
4.67
2.38

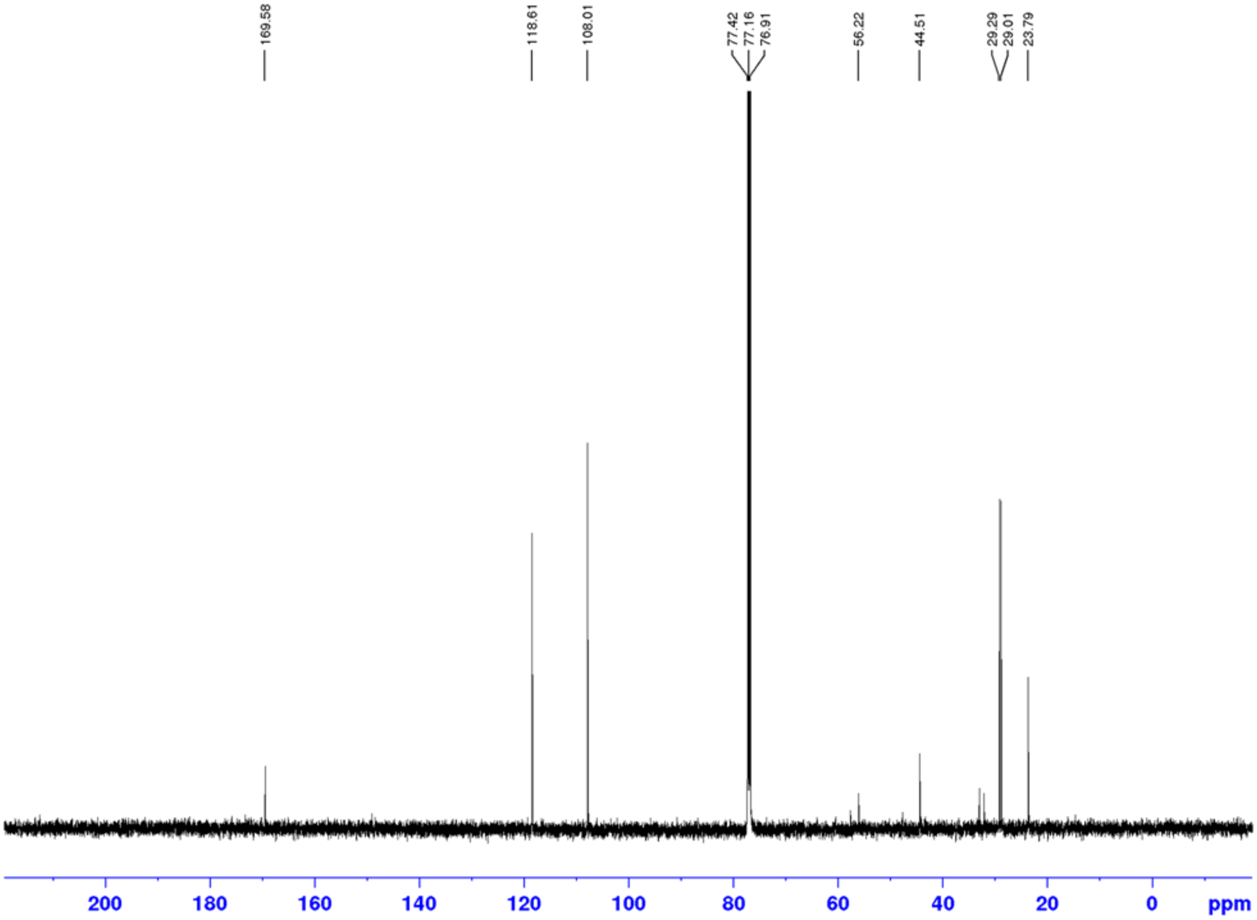

169.58
118.61
108.01
77.42
77.16
76.91
56.22
44.51
29.29
29.01
23.79
200
180
160
140
120
100
80
60
40
20
0
ppm

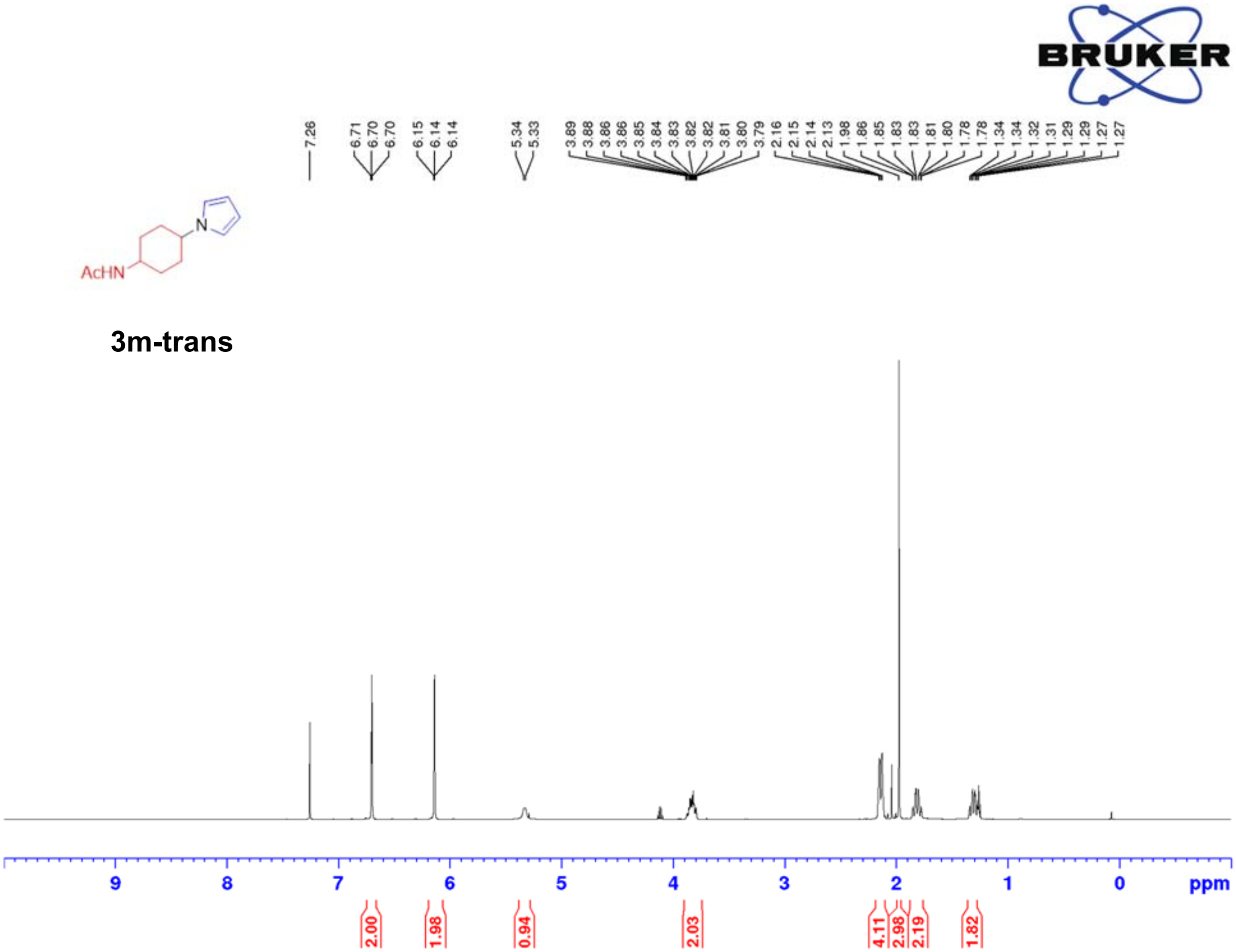

AcHN
N
7.26
6.71
6.70
6.70
6.15
6.14
6.14
5.34
5.33
3.89
3.88
3.86
3.86
3.85
3.84
3.83
3.82
3.82
3.81
3.80
3.79
2.16
2.15
2.14
2.13
1.98
1.86
1.85
1.83
1.83
1.81
1.80
1.78
1.34
1.34
1.32
1.31
1.29
1.29
1.27
1.27
9
8
7
6
5
4
3
2
1
0
ppm
2.00
1.98
0.94
2.03
4.11
2.98
2.19
1.82

3m-trans

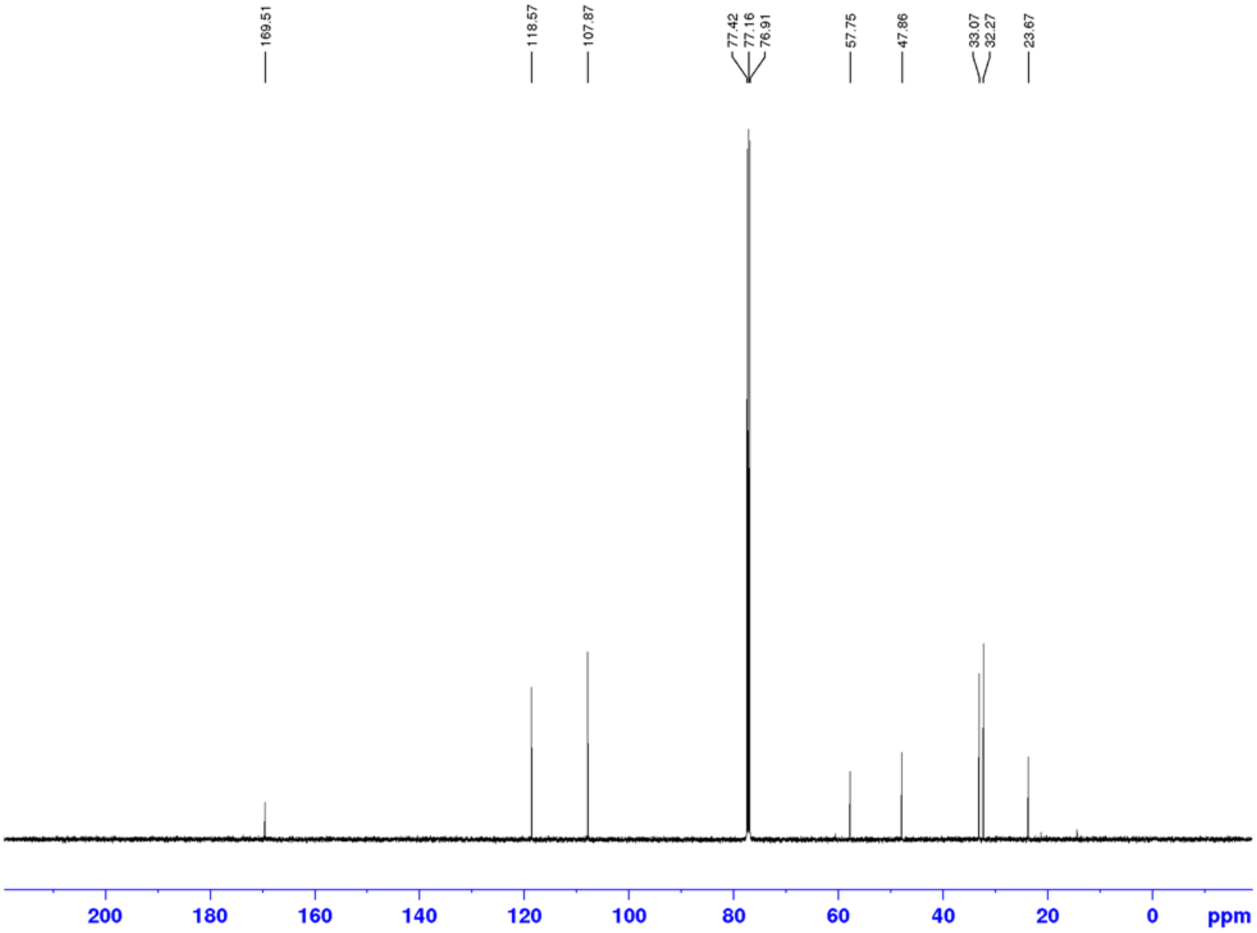
169.51
118.57
107.87
77.42
77.16
76.91
57.75
47.86
33.07
32.27
23.67
200
180
160
140
120
100
80
60
40
20
0
ppm

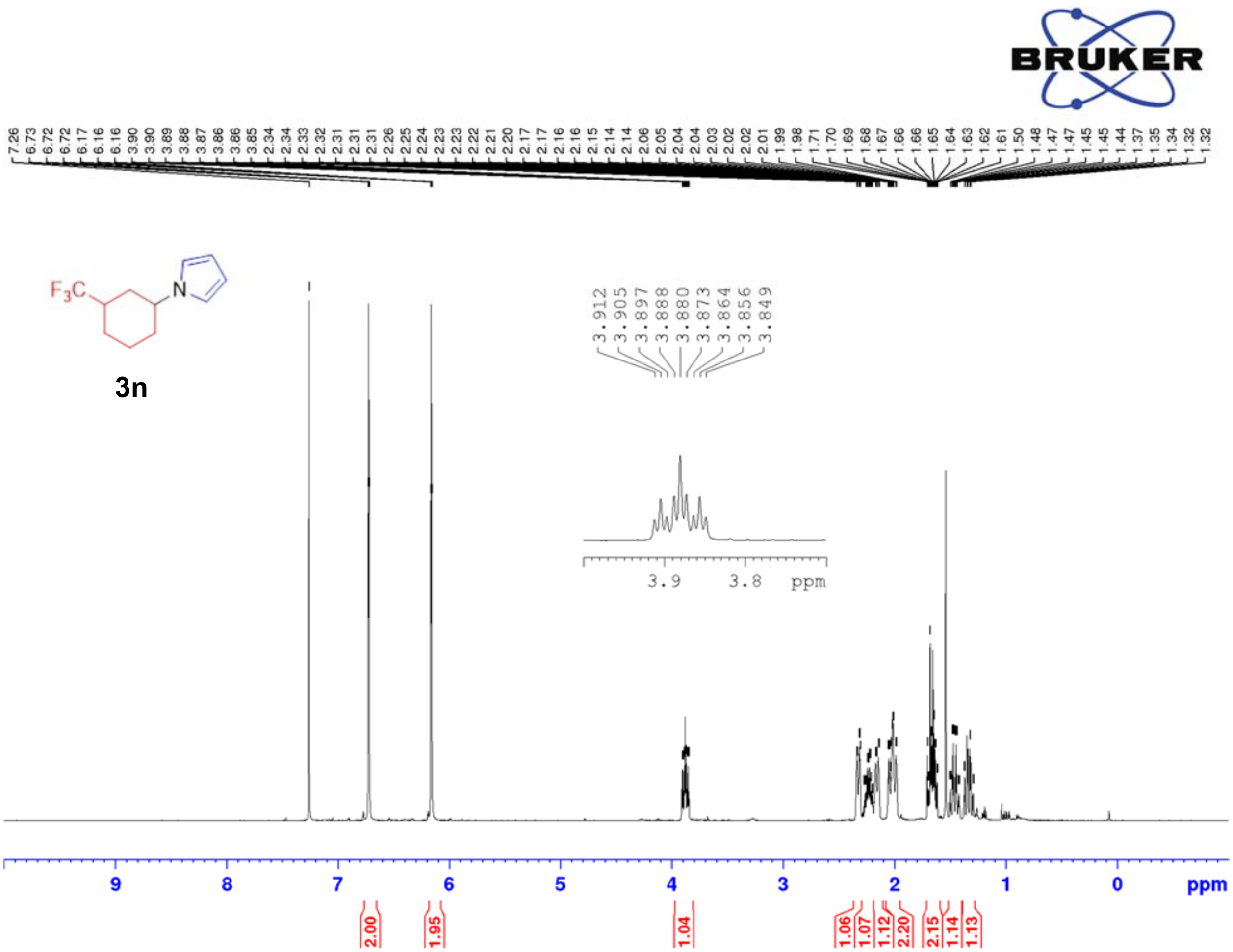
F3C
N
3n
3.912
3.905
3.897
3.888
3.880
3.873
3.864
3.856
3.849
3.9
3.8
ppm
9
8
7
6
5
4
3
2
1
0
ppm
2.00
1.95
1.04
1.06
1.07
1.12
2.20
2.15
1.14
1.13

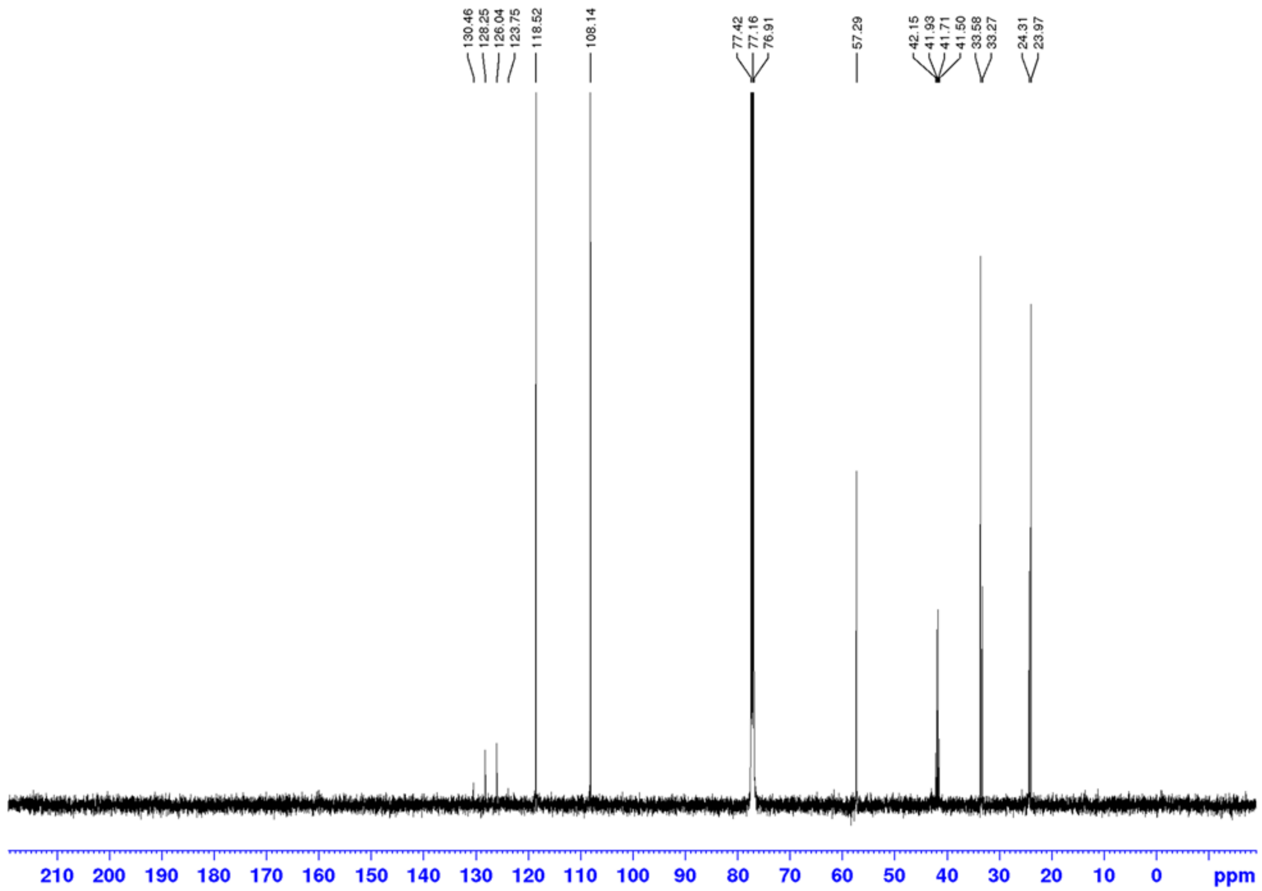
130.46
128.25
126.04
123.75
118.52
108.14
77.42
77.16
76.91
57.29
42.15
41.93
41.71
41.50
33.58
33.27
24.31
23.97
210 200 190 180 170 160 150 140 130 120 110 100 90 80 70 60 50 40 30 20 10 0 ppm

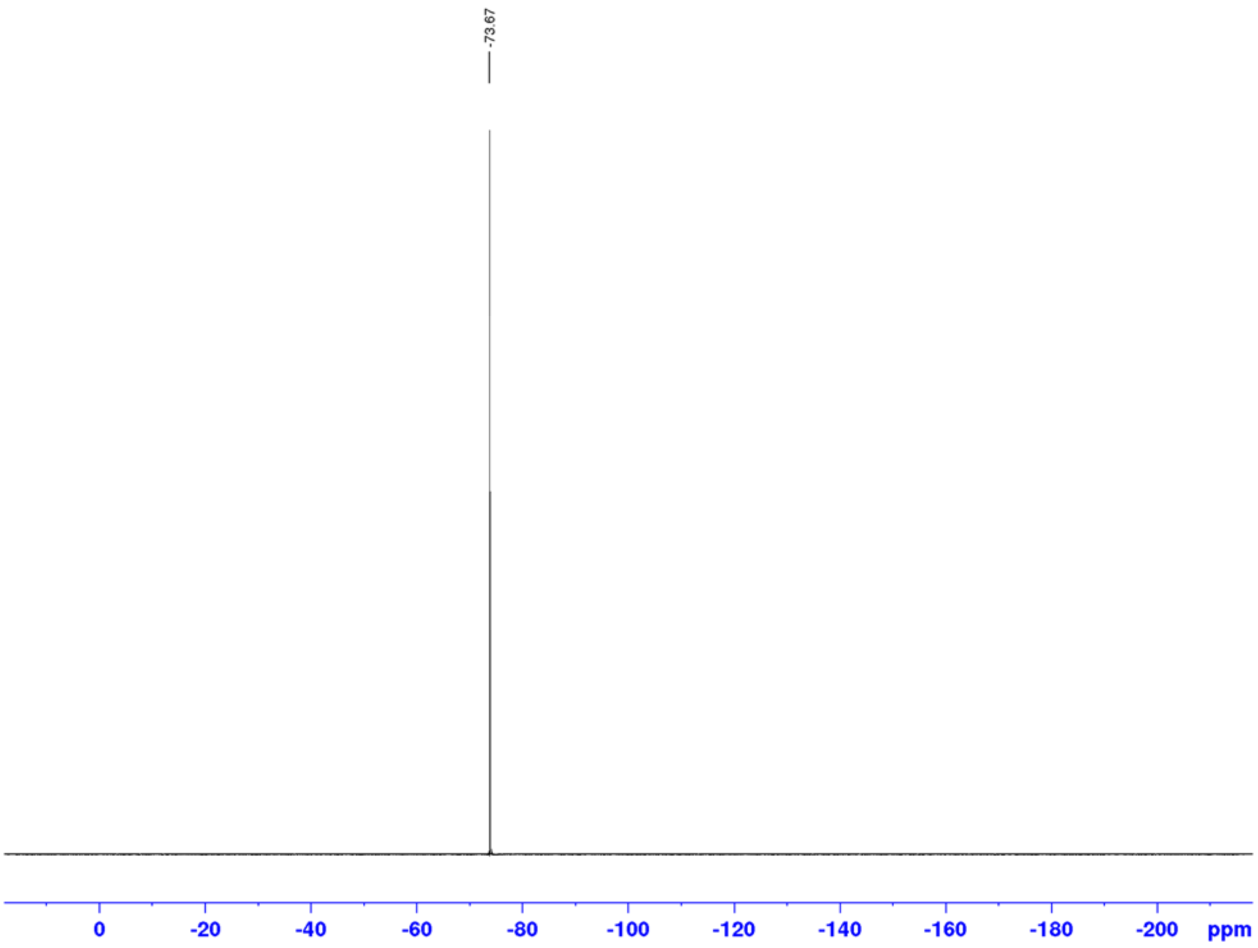

-73.67
0
-20
-40
-60
-80
-100
-120
-140
-160
-180
-200
ppm

3o

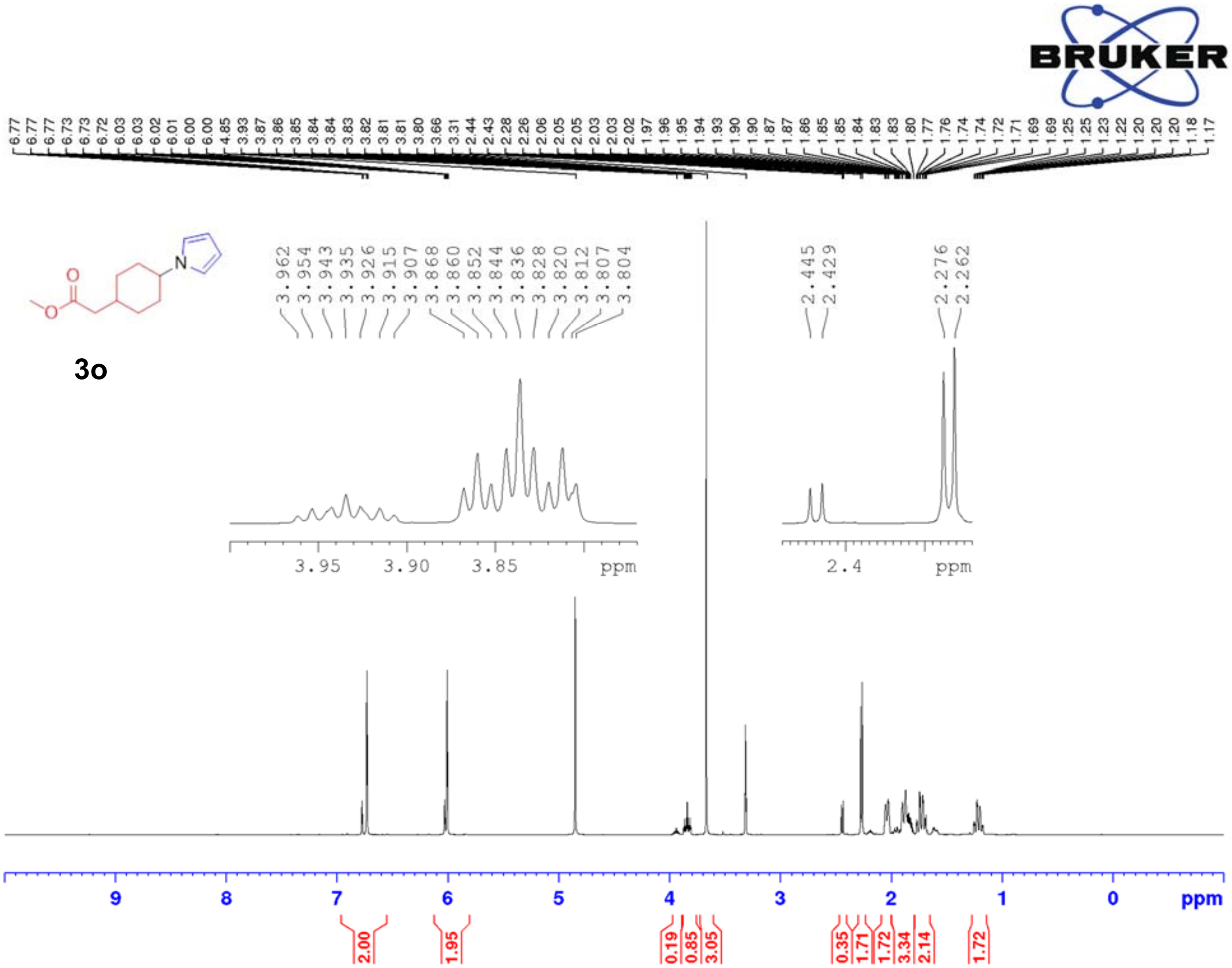

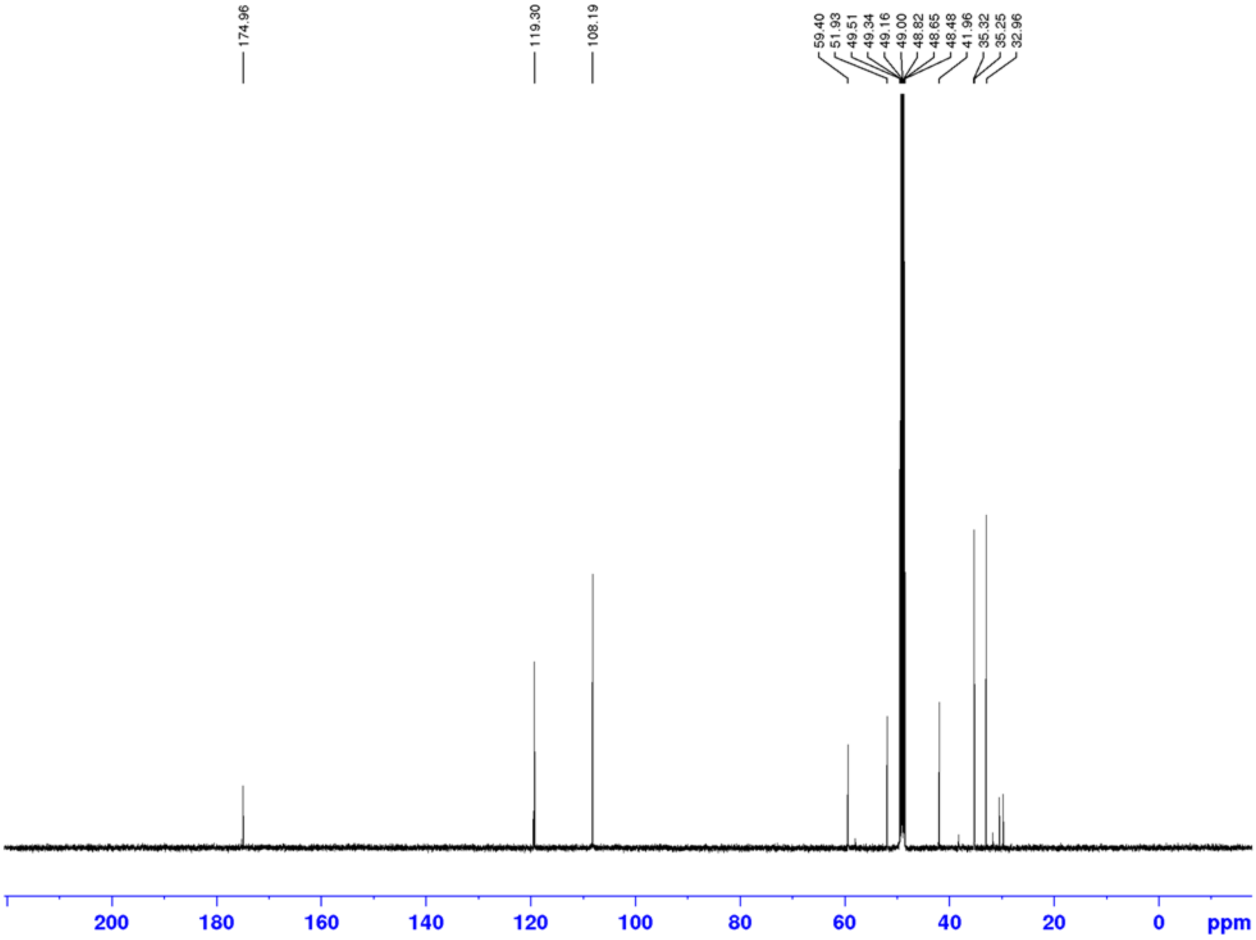

174.96
119.30
108.19
59.40
51.93
49.51
49.34
49.16
49.00
48.82
48.65
48.48
41.96
35.32
35.25
32.96
200
180
160
140
120
100
80
60
40
20
0
ppm

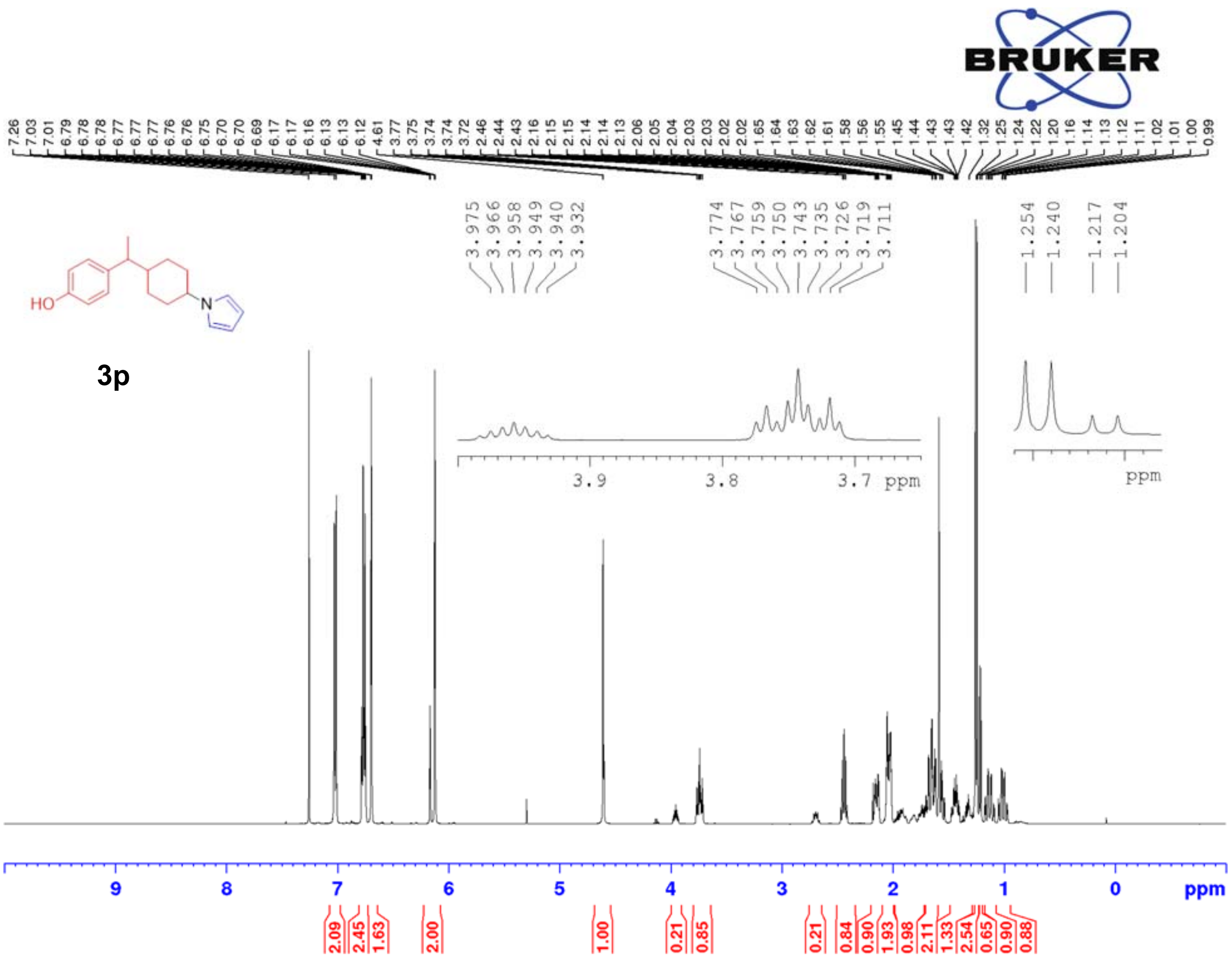

3p

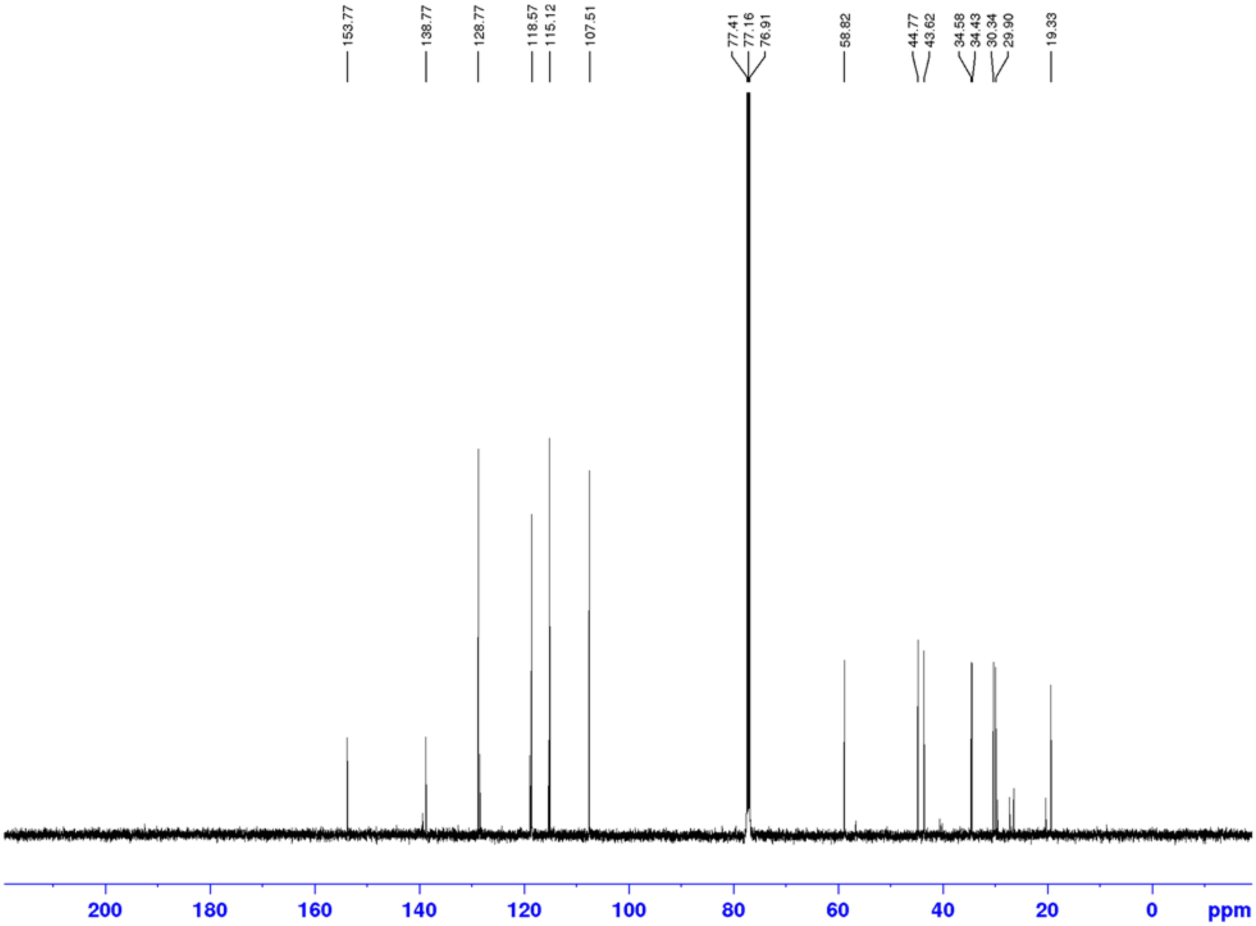
153.77
138.77
128.77
118.57
115.12
107.51
77.41
77.16
76.91
58.82
44.77
43.62
34.58
34.43
30.34
29.90
19.33
200
180
160
140
120
100
80
60
40
20
0
ppm

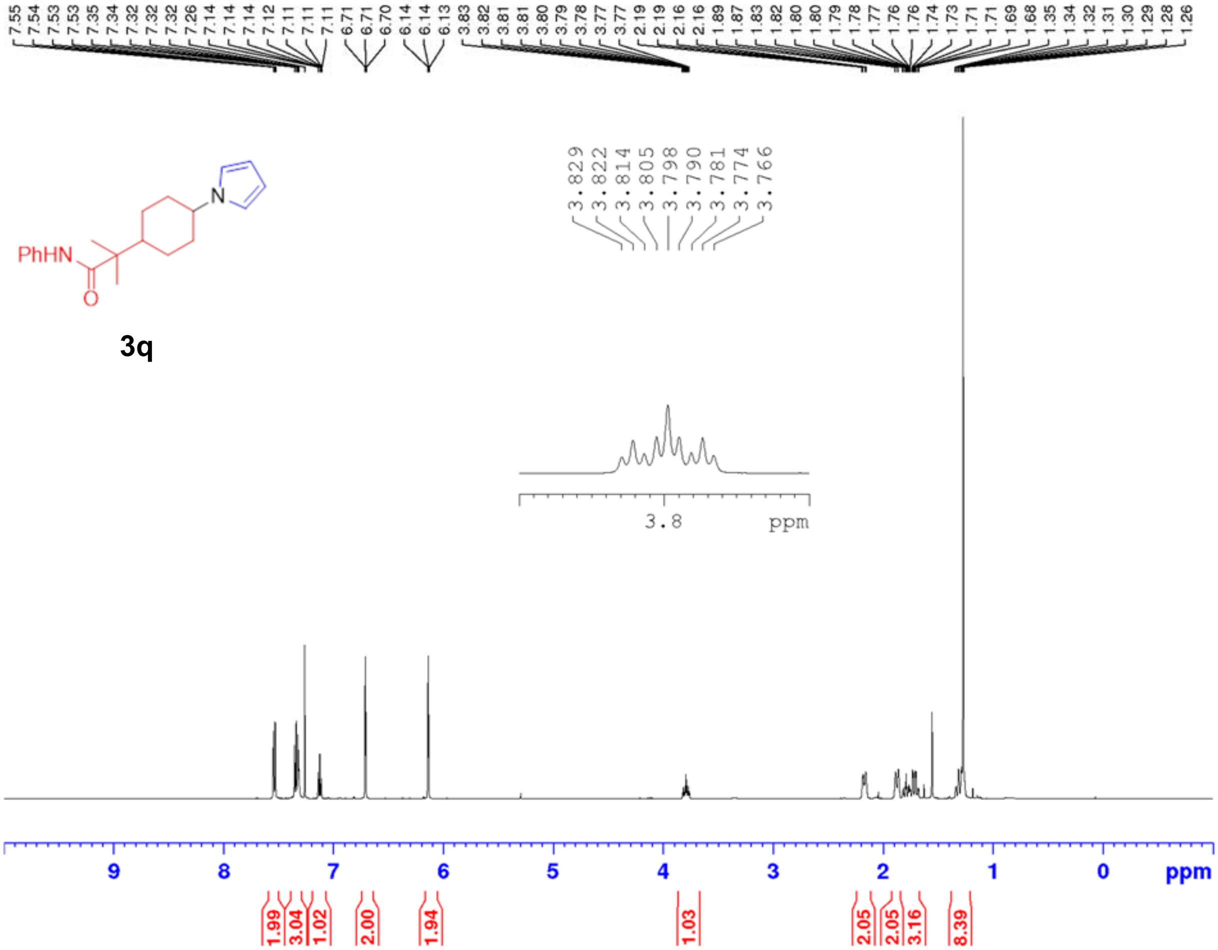
PhHN
O
N
7.55
7.54
7.53
7.53
7.35
7.34
7.32
7.32
7.32
7.26
7.14
7.14
7.14
7.12
7.11
7.11
7.11
6.71
6.71
6.70
6.14
6.14
6.13
3.83
3.82
3.81
3.81
3.80
3.79
3.78
3.77
3.77
2.19
2.19
2.16
2.16
1.89
1.87
1.83
1.82
1.80
1.80
1.79
1.78
1.77
1.76
1.76
1.74
1.73
1.71
1.71
1.69
1.68
1.35
1.34
1.32
1.31
1.30
1.29
1.28
1.26
3.829
3.822
3.814
3.805
3.798
3.790
3.781
3.774
3.766
3.8
ppm
9
8
7
6
5
4
3
2
1
0
ppm
1.99
3.04
1.02
2.00
1.94
1.03
2.05
2.05
3.16
8.39

3q

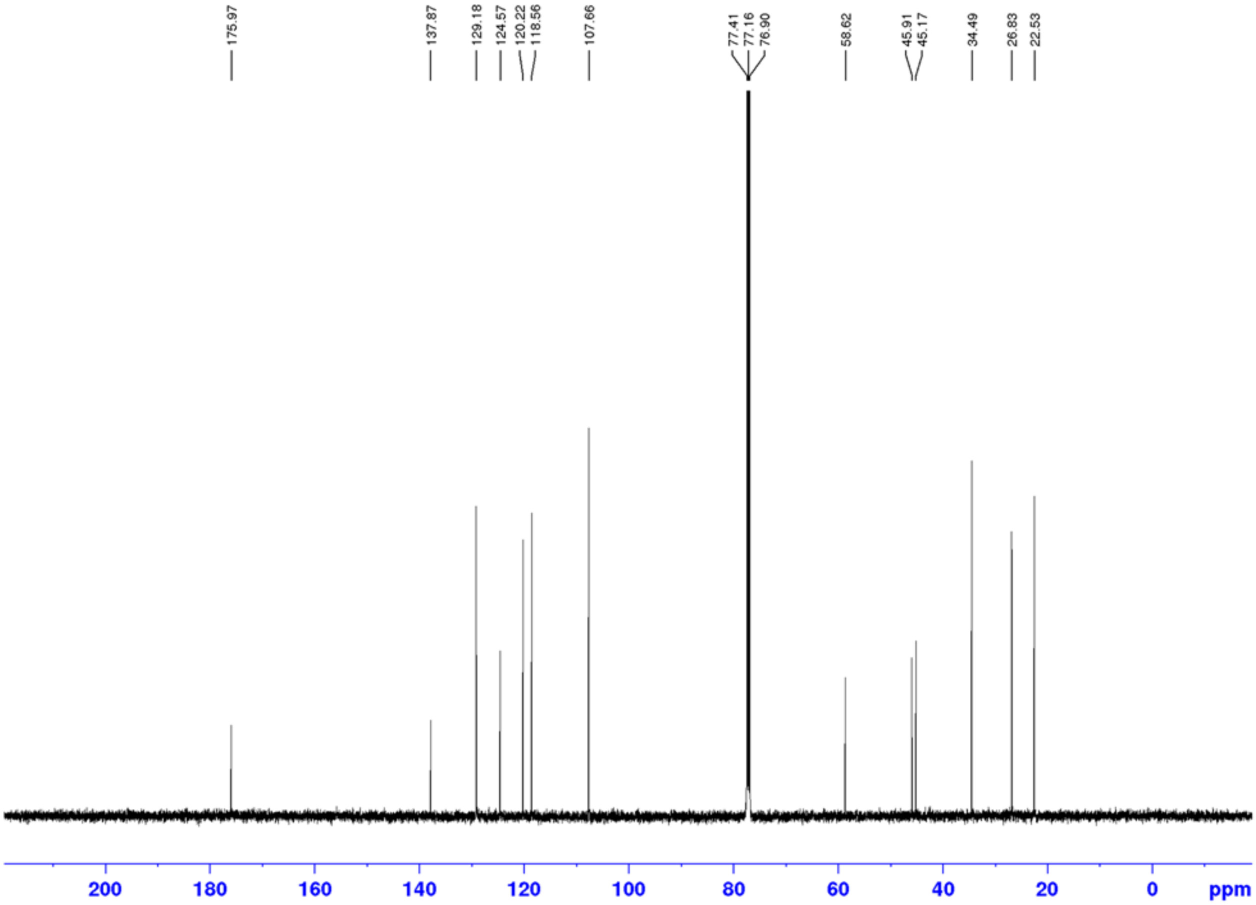
175.97
137.87
129.18
124.57
120.22
118.56
107.66
77.41
77.16
76.90
58.62
45.91
45.17
34.49
26.83
22.53
200
180
160
140
120
100
80
60
40
20
0
ppm

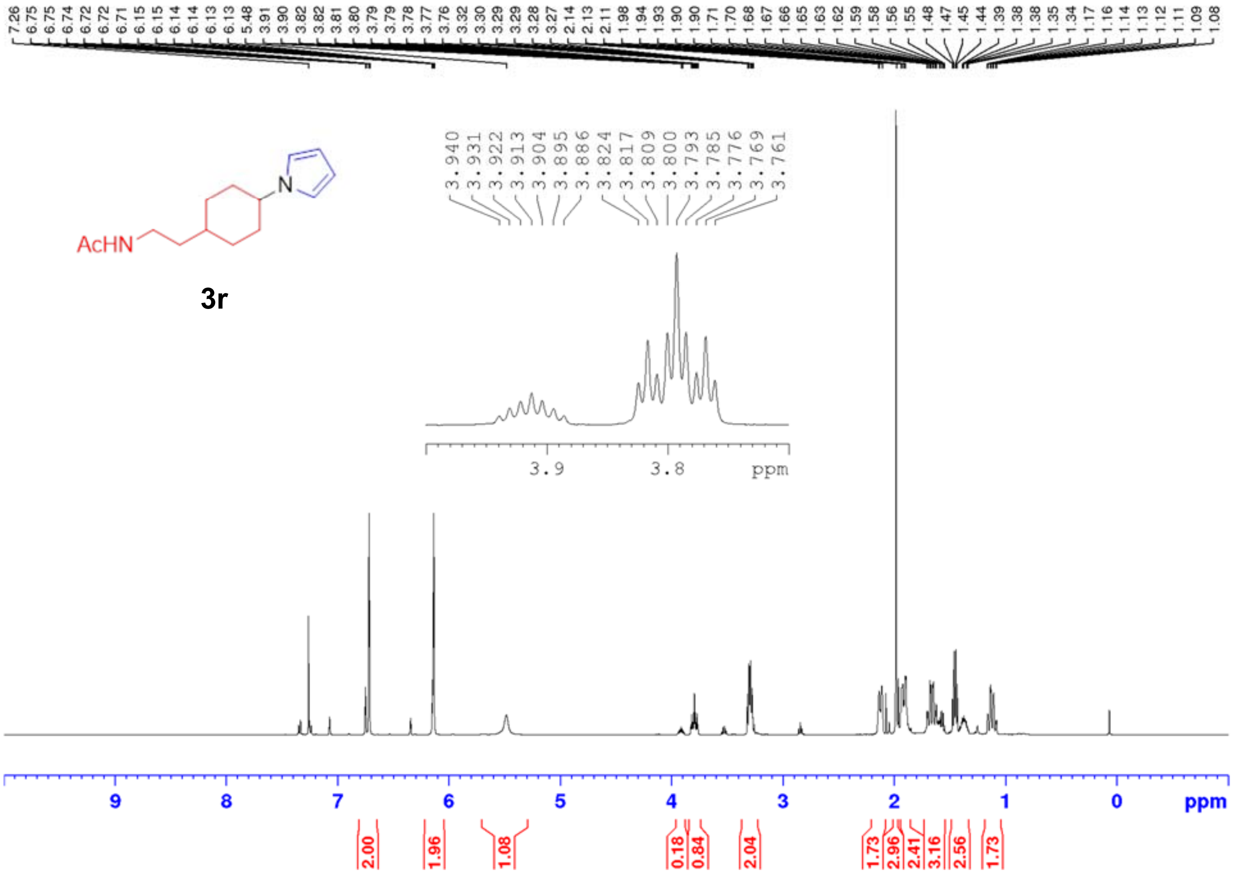

3r
7.26
6.75
6.75
6.74
6.72
6.72
6.71
6.15
6.15
6.14
6.14
6.13
6.13
5.48
3.91
3.90
3.82
3.81
3.80
3.79
3.79
3.78
3.77
3.76
3.32
3.30
3.29
3.29
3.28
3.27
2.14
2.13
2.11
1.98
1.94
1.93
1.90
1.90
1.71
1.70
1.68
1.67
1.66
1.65
1.63
1.62
1.59
1.58
1.56
1.55
1.48
1.47
1.45
1.44
1.39
1.38
1.38
1.35
1.34
1.17
1.16
1.14
1.13
1.12
1.11
1.09
1.08
3.940
3.931
3.922
3.913
3.904
3.895
3.886
3.824
3.817
3.809
3.800
3.793
3.785
3.776
3.769
3.761
3.9
3.8
ppm
9
8
7
6
5
4
3
2
1
0
ppm
2.00
1.96
1.08
0.18
0.84
2.04
1.73
2.96
2.96
2.41
3.16
2.56
1.73

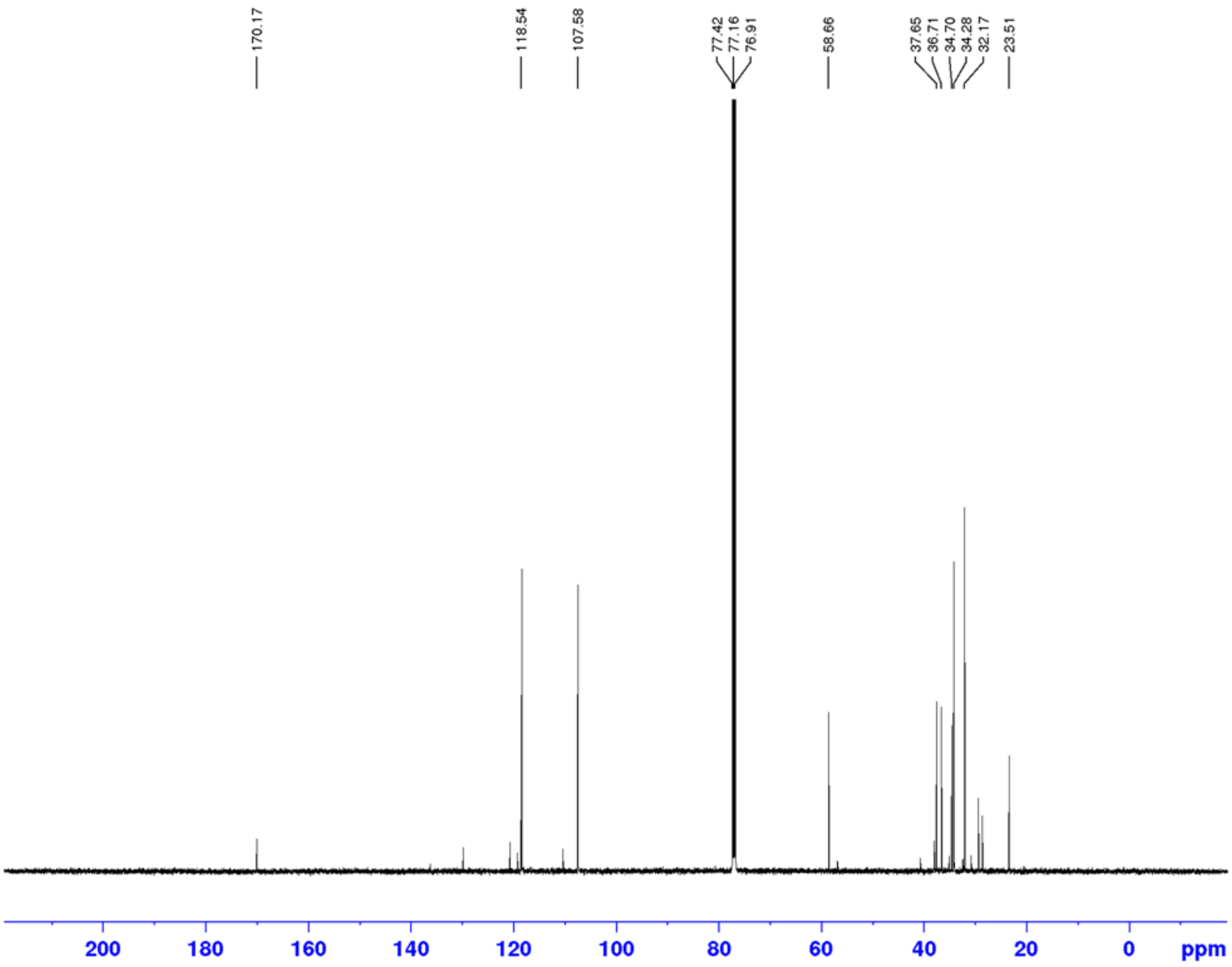
170.17
118.54
107.58
77.42
77.16
76.91
58.66
37.65
36.71
34.70
34.28
32.17
23.51
200
180
160
140
120
100
80
60
40
20
0
ppm

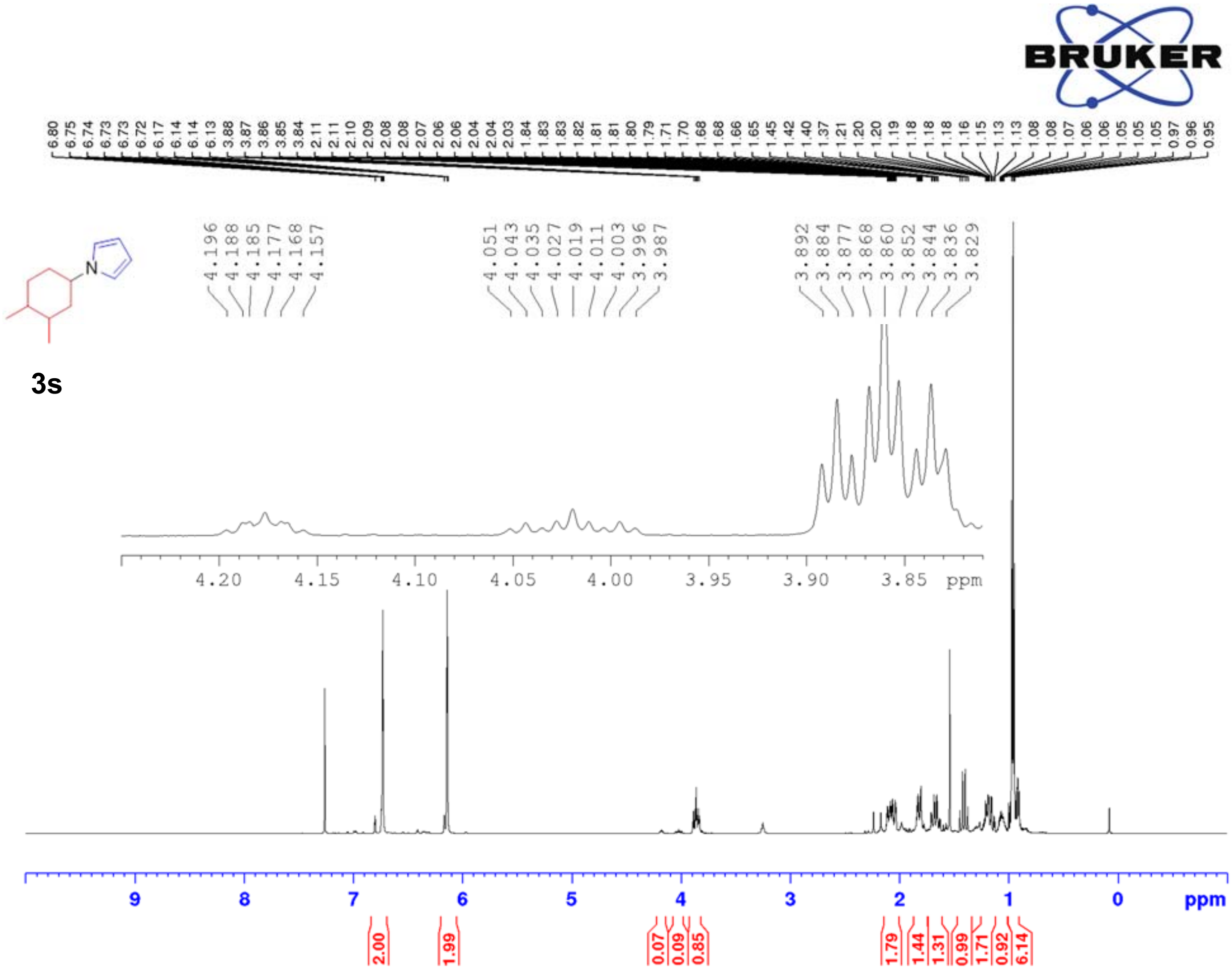

3s

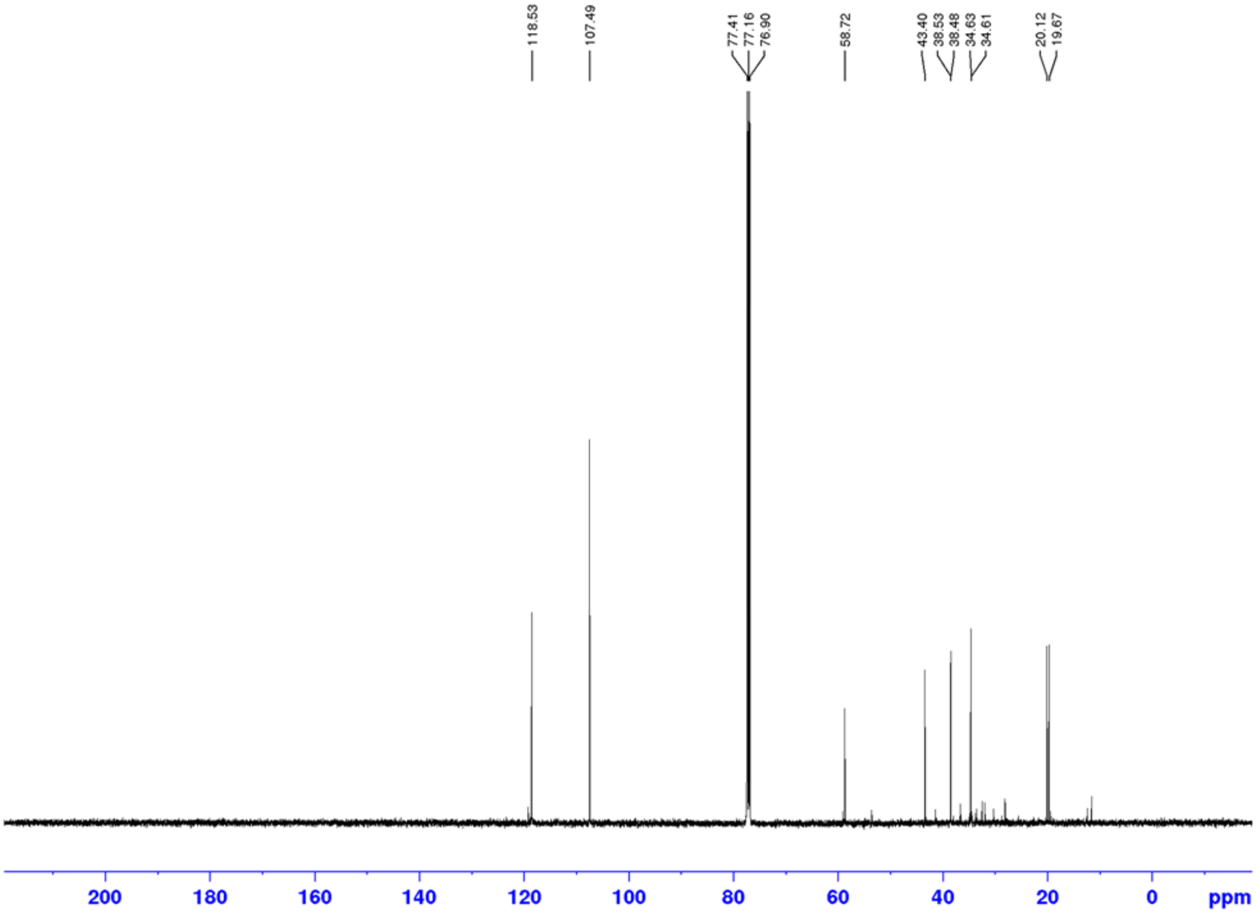
118.53
107.49
77.41
77.16
76.90
58.72
43.40
38.53
38.48
34.63
34.61
20.12
19.67
200
180
160
140
120
100
80
60
40
20
0
ppm

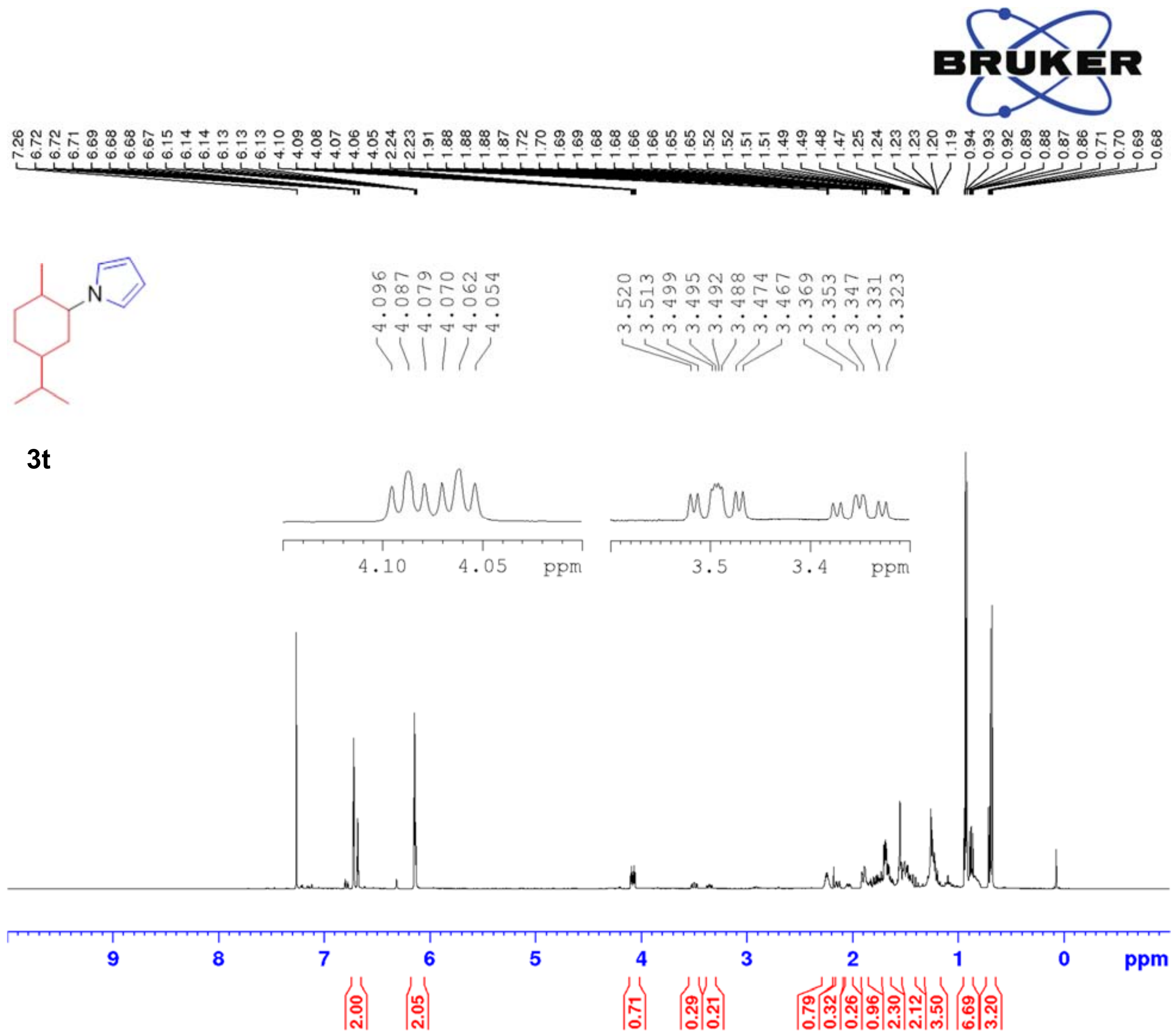
BRUKER
7.26 6.72 6.72 6.71 6.69 6.68 6.68 6.67 6.15 6.14 6.14 6.13 6.13 6.13 4.10 4.09 4.08 4.07 4.06 4.05 2.24 2.23 1.91 1.88 1.88 1.88 1.87 1.72 1.70 1.69 1.69 1.68 1.68 1.66 1.66 1.65 1.65 1.52 1.52 1.51 1.51 1.49 1.48 1.47 1.25 1.24 1.23 1.23 1.20 1.19 0.94 0.93 0.92 0.89 0.88 0.87 0.86 0.71 0.70 0.69 0.68
N
4.096 4.087 4.079 4.070 4.062 4.054
3.520 3.513 3.499 3.495 3.492 3.488 3.474 3.467 3.369 3.353 3.347 3.331 3.323
4.10 4.05 ppm
3.5 3.4 ppm
9 8 7 6 5 4 3 2 1 0 ppm
2.00 2.05 0.71 0.29 0.21 0.79 0.32 0.26 0.96 2.30 2.12 3.50 6.69 3.20

3t

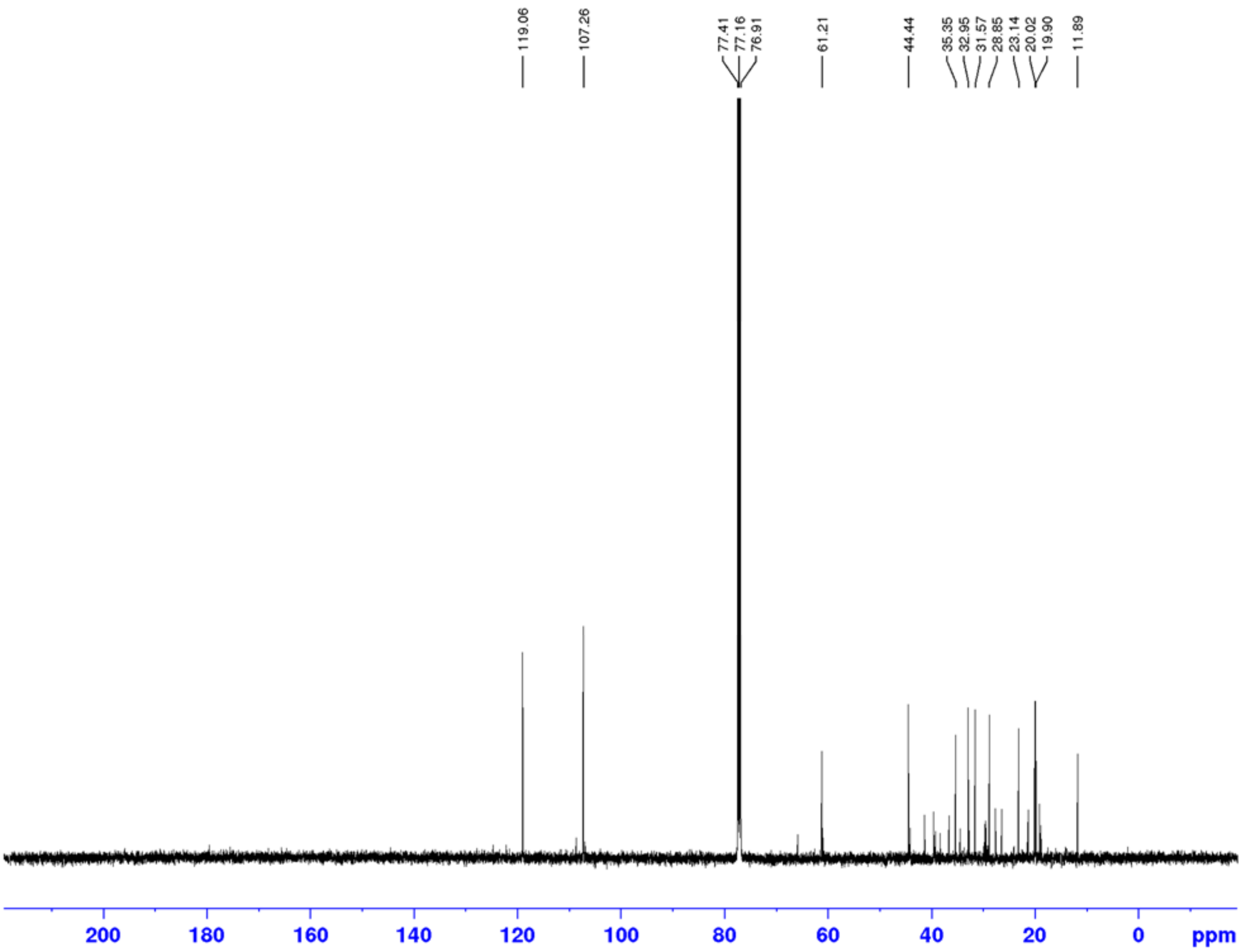
119.06
107.26
77.41
77.16
76.91
61.21
44.44
35.35
32.95
31.57
28.85
23.14
20.02
19.90
11.89
200
180
160
140
120
100
80
60
40
20
0
ppm

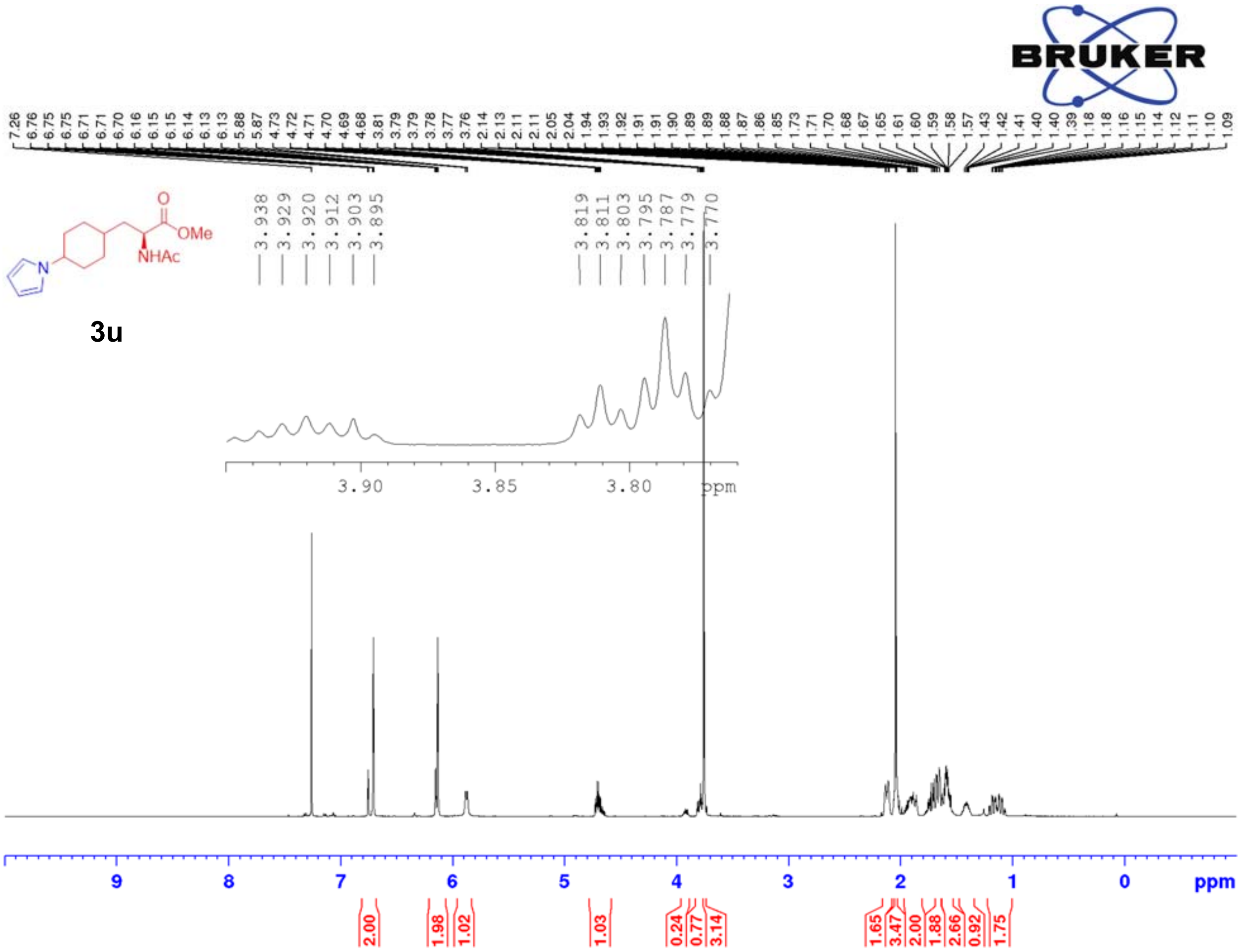
BRUKER
OMe
NHAc
N
3u
3.938
3.929
3.920
3.912
3.903
3.895
3.819
3.811
3.803
3.795
3.787
3.779
3.770
3.90
3.85
3.80
ppm
9
8
7
6
5
4
3
2
1
0
ppm
2.00
1.98
1.02
1.03
0.24
0.77
3.14
1.65
3.47
2.00
1.88
2.66
0.92
1.75

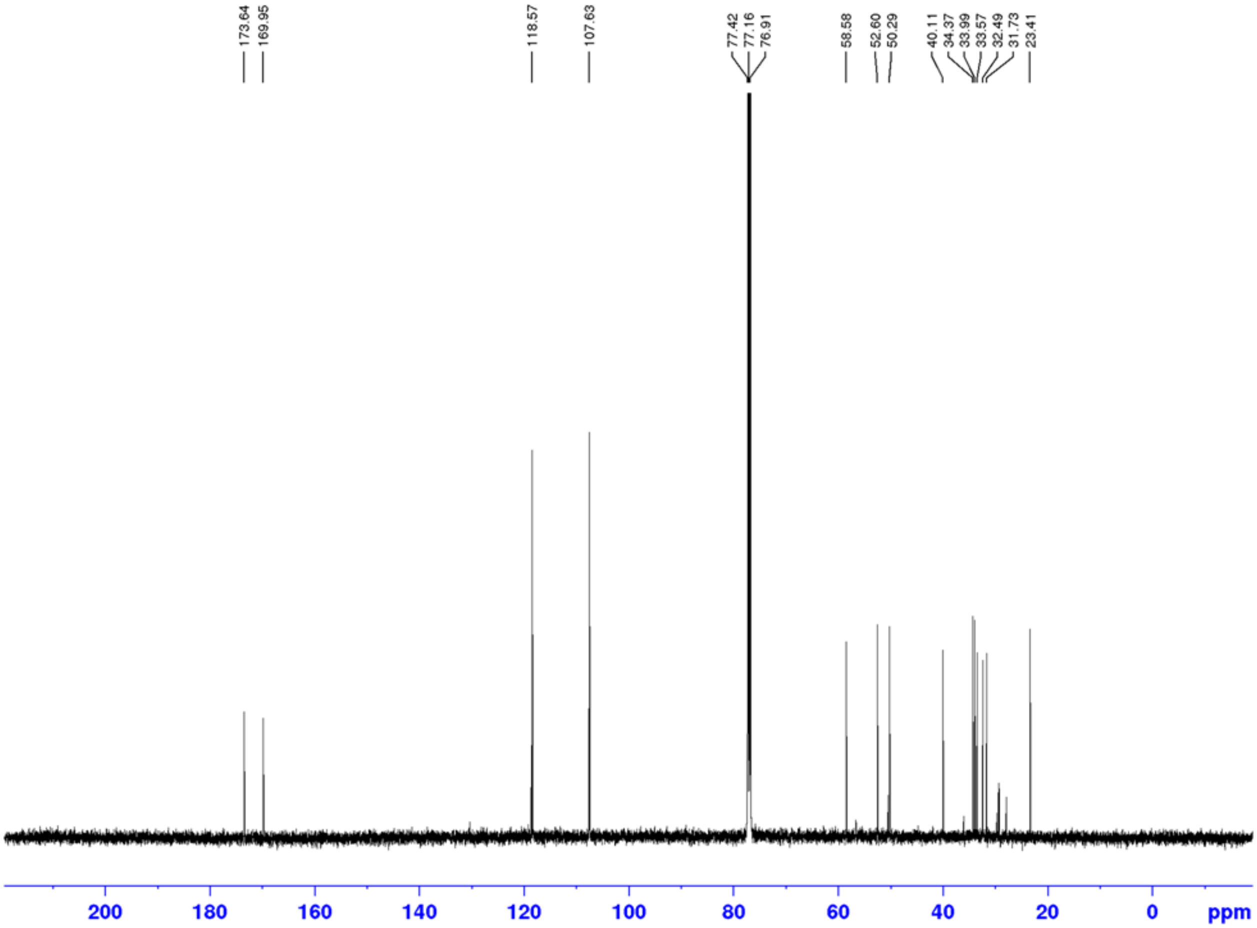
173.64
169.95
118.57
107.63
77.42
77.16
76.91
58.58
52.60
50.29
40.11
34.37
33.99
33.57
32.49
31.73
23.41
200
180
160
140
120
100
80
60
40
20
0
ppm

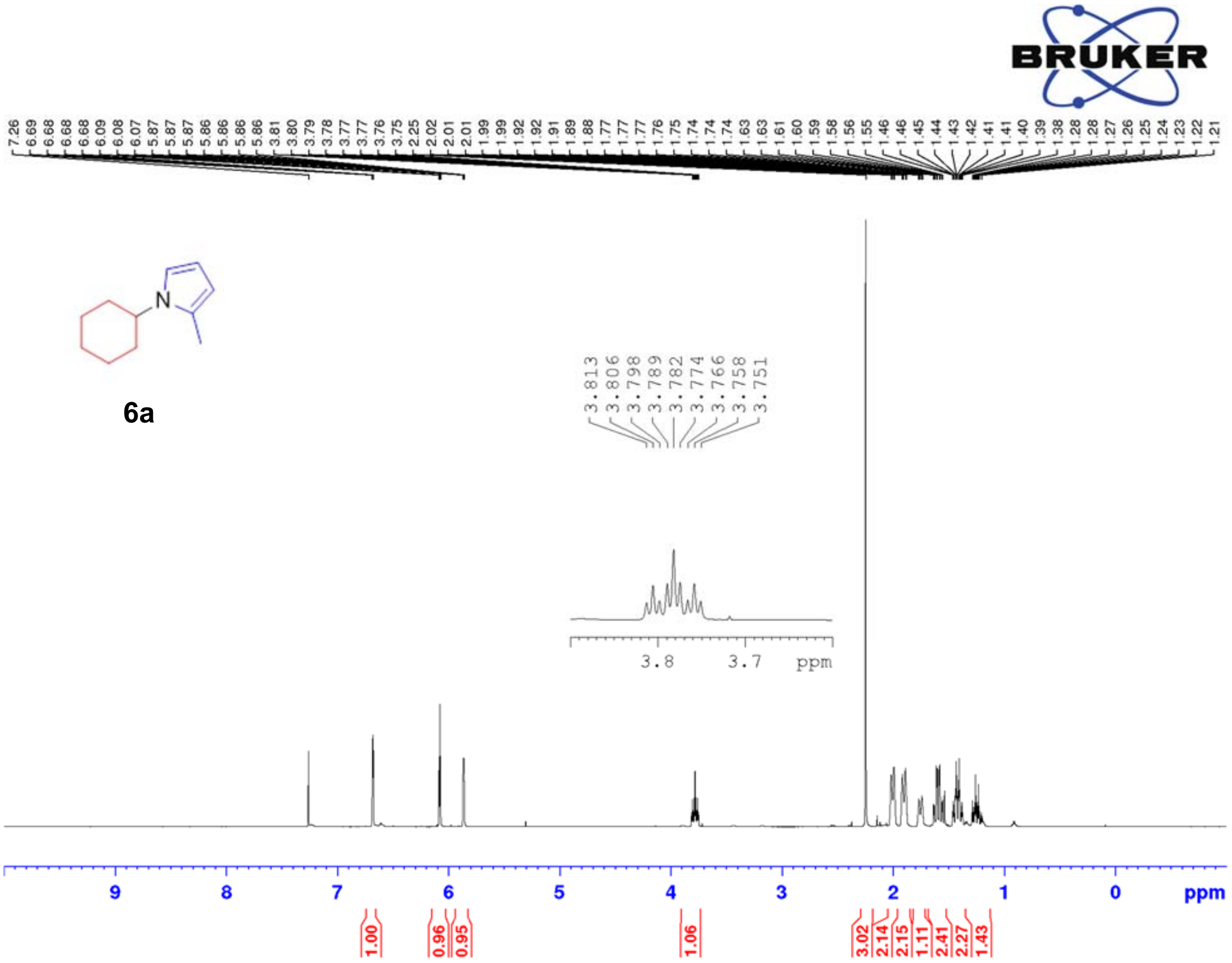
BRUKER
7.26 6.69 6.68 6.68 6.68 6.09 6.08 6.07 5.87 5.87 5.87 5.86 5.86 5.86 5.86 3.81 3.80 3.79 3.78 3.77 3.77 3.76 3.75 2.25 2.02 2.01 2.01 1.99 1.99 1.92 1.92 1.91 1.89 1.88 1.77 1.77 1.77 1.76 1.75 1.74 1.74 1.74 1.63 1.63 1.61 1.60 1.59 1.58 1.56 1.55 1.46 1.46 1.45 1.44 1.43 1.42 1.41 1.40 1.39 1.38 1.28 1.28 1.27 1.26 1.25 1.24 1.23 1.22 1.21
N
6a
3.813 3.806 3.798 3.789 3.782 3.774 3.766 3.758 3.751
3.8
3.7
ppm
9 8 7 6 5 4 3 2 1 0 ppm
1.00 0.96 0.95 1.06 3.02 2.14 2.15 1.11 2.41 2.27 1.43

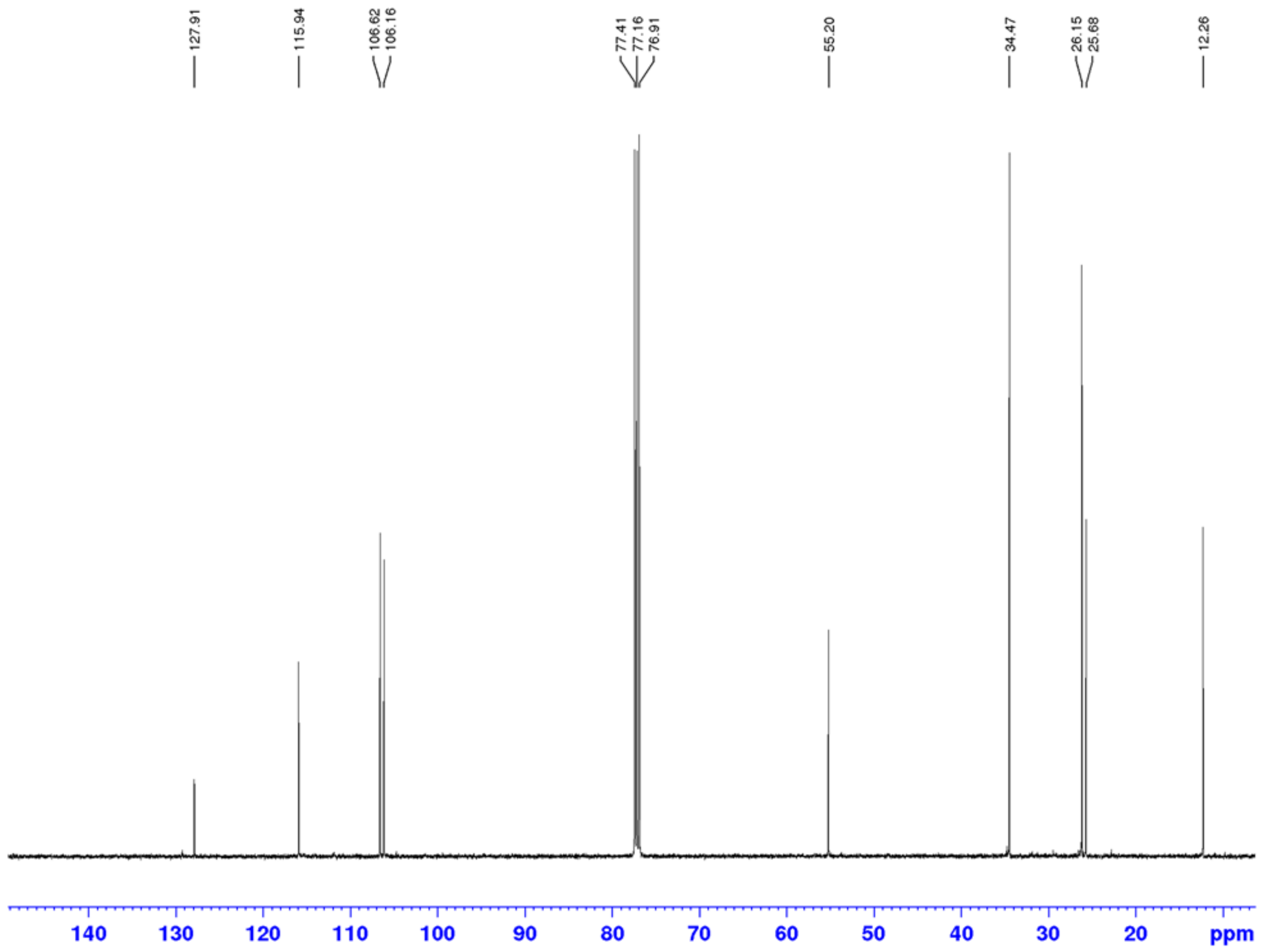
127.91
115.94
106.62
106.16
77.41
77.16
76.91
55.20
34.47
26.15
25.68
12.26
140 130 120 110 100 90 80 70 60 50 40 30 20 ppm

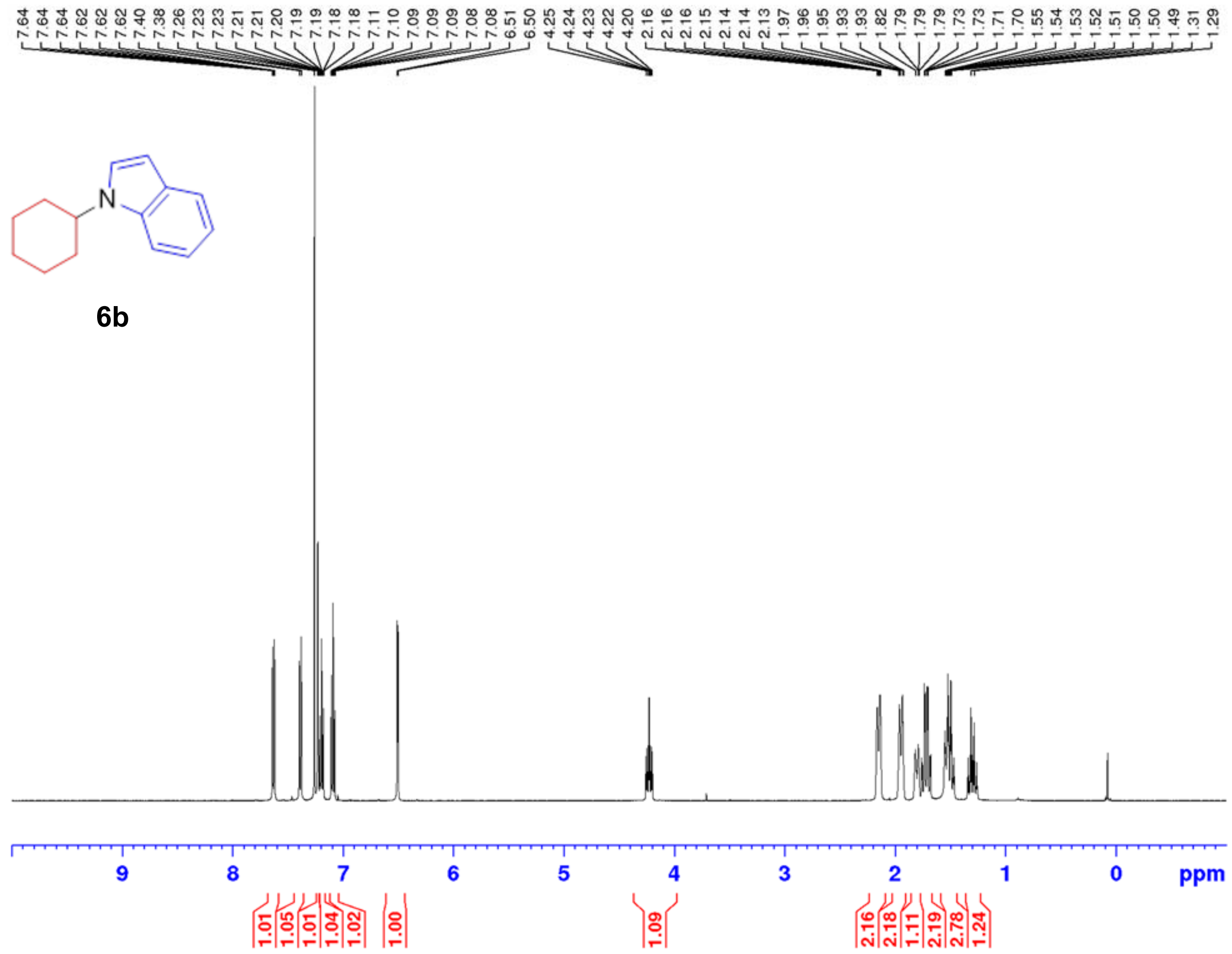
7.64 7.64 7.64 7.62 7.62 7.62 7.40 7.38 7.26 7.23 7.23 7.21 7.21 7.20 7.19 7.19 7.18 7.18 7.11 7.10 7.09 7.09 7.08 7.08 6.51 6.50 4.25 4.24 4.23 4.22 4.20 2.16 2.16 2.15 2.14 2.14 2.13 1.97 1.96 1.95 1.93 1.93 1.82 1.79 1.79 1.79 1.73 1.73 1.71 1.70 1.55 1.54 1.53 1.52 1.51 1.50 1.50 1.49 1.31 1.29
N
6b
9
8
7
6
5
4
3
2
1
0
ppm
1.01
1.05
1.01
1.04
1.02
1.00
1.09
2.16
2.18
1.11
2.19
2.78
1.24

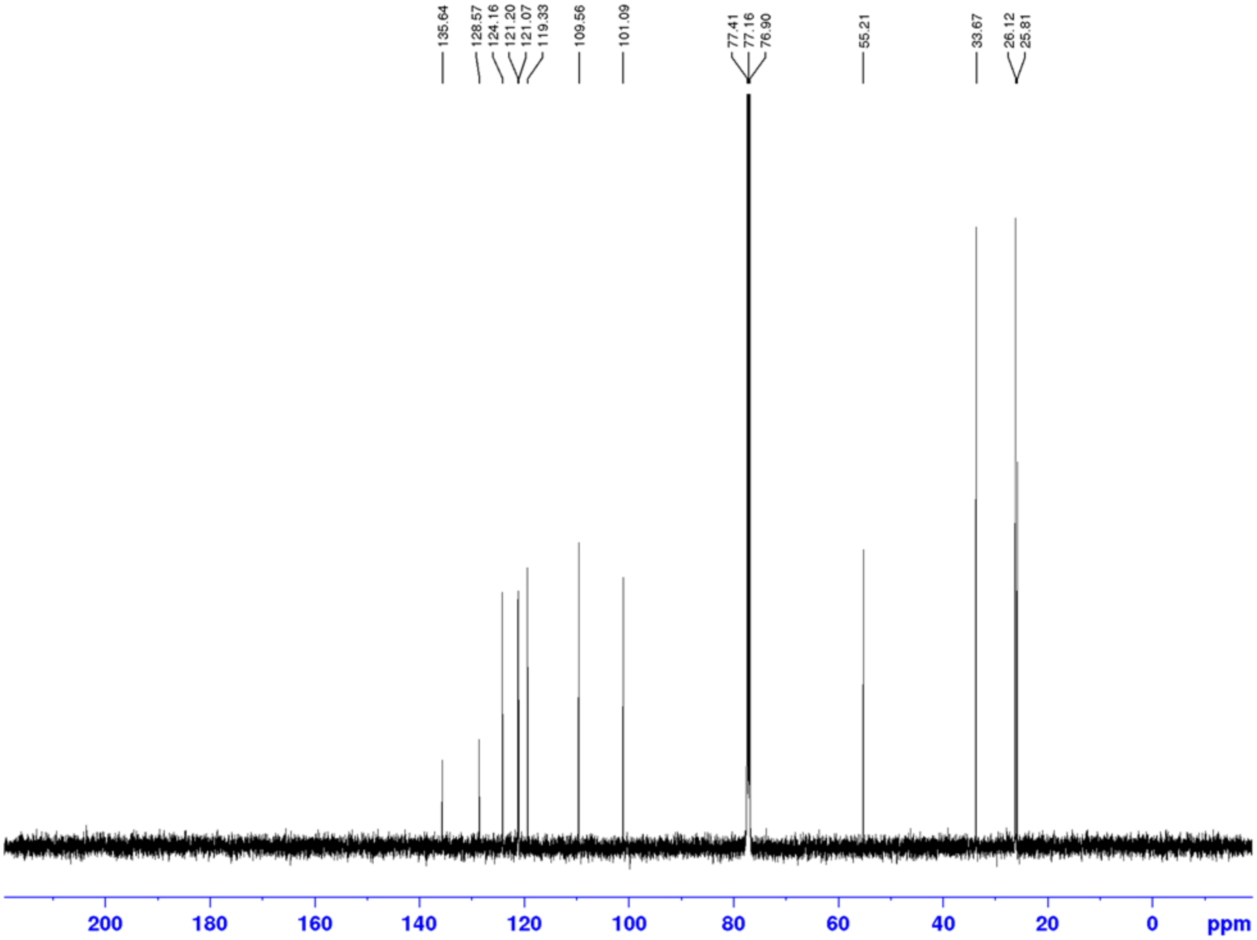
135.64
128.57
124.16
121.20
121.07
119.33
109.56
101.09
77.41
77.16
76.90
55.21
33.67
26.12
25.81
200
180
160
140
120
100
80
60
40
20
0
ppm

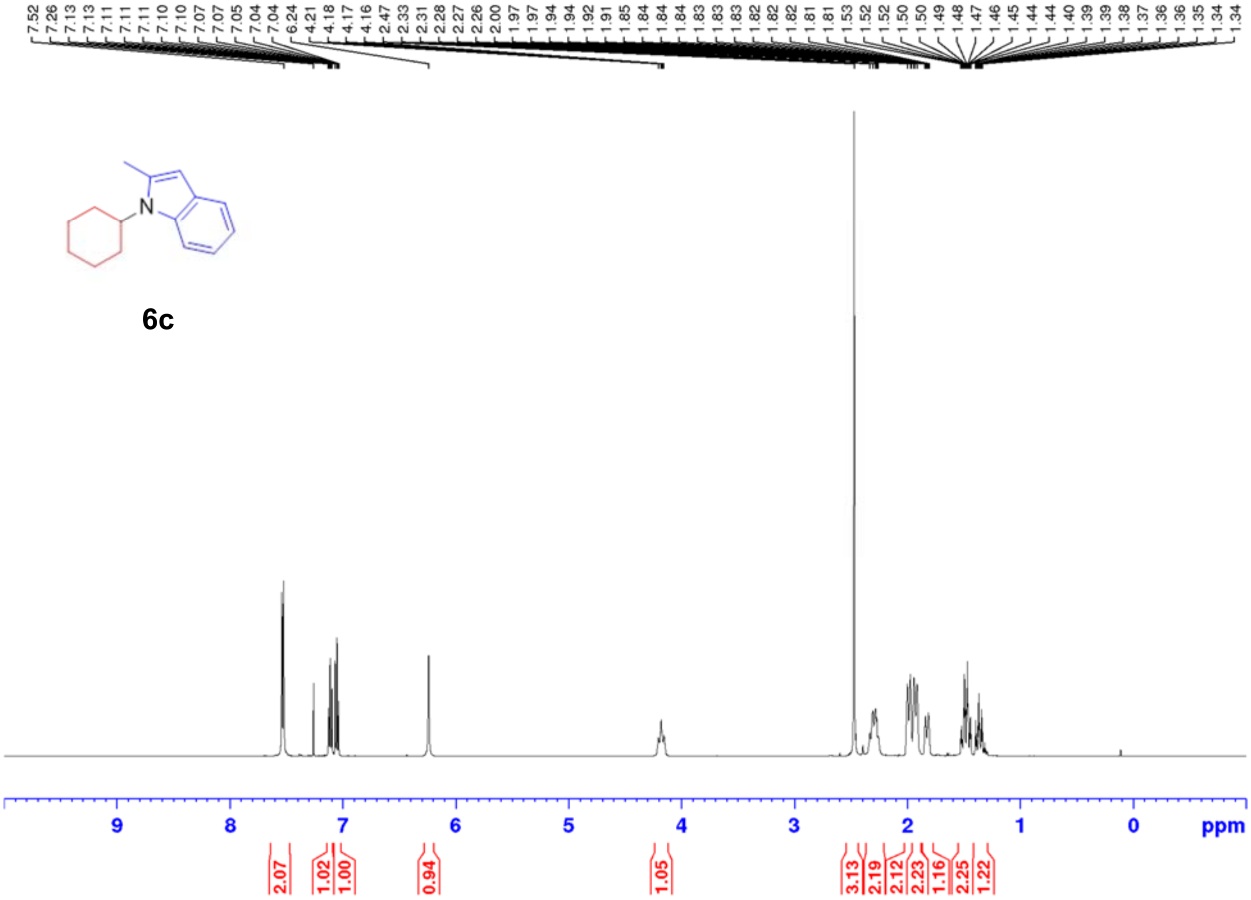
N
6c
7.52
7.26
7.13
7.13
7.11
7.11
7.11
7.10
7.07
7.07
7.05
7.04
7.04
6.24
4.21
4.18
4.17
4.16
2.47
2.33
2.31
2.28
2.27
2.26
2.00
1.97
1.97
1.94
1.94
1.92
1.91
1.85
1.84
1.84
1.84
1.83
1.83
1.83
1.82
1.82
1.82
1.81
1.81
1.53
1.52
1.52
1.50
1.50
1.49
1.48
1.47
1.46
1.45
1.44
1.44
1.40
1.39
1.39
1.38
1.37
1.36
1.36
1.35
1.34
1.34
9
8
7
6
5
4
3
2
1
0
ppm
2.07
1.02
1.00
0.94
1.05
3.13
2.19
2.12
2.23
1.16
2.25
1.22

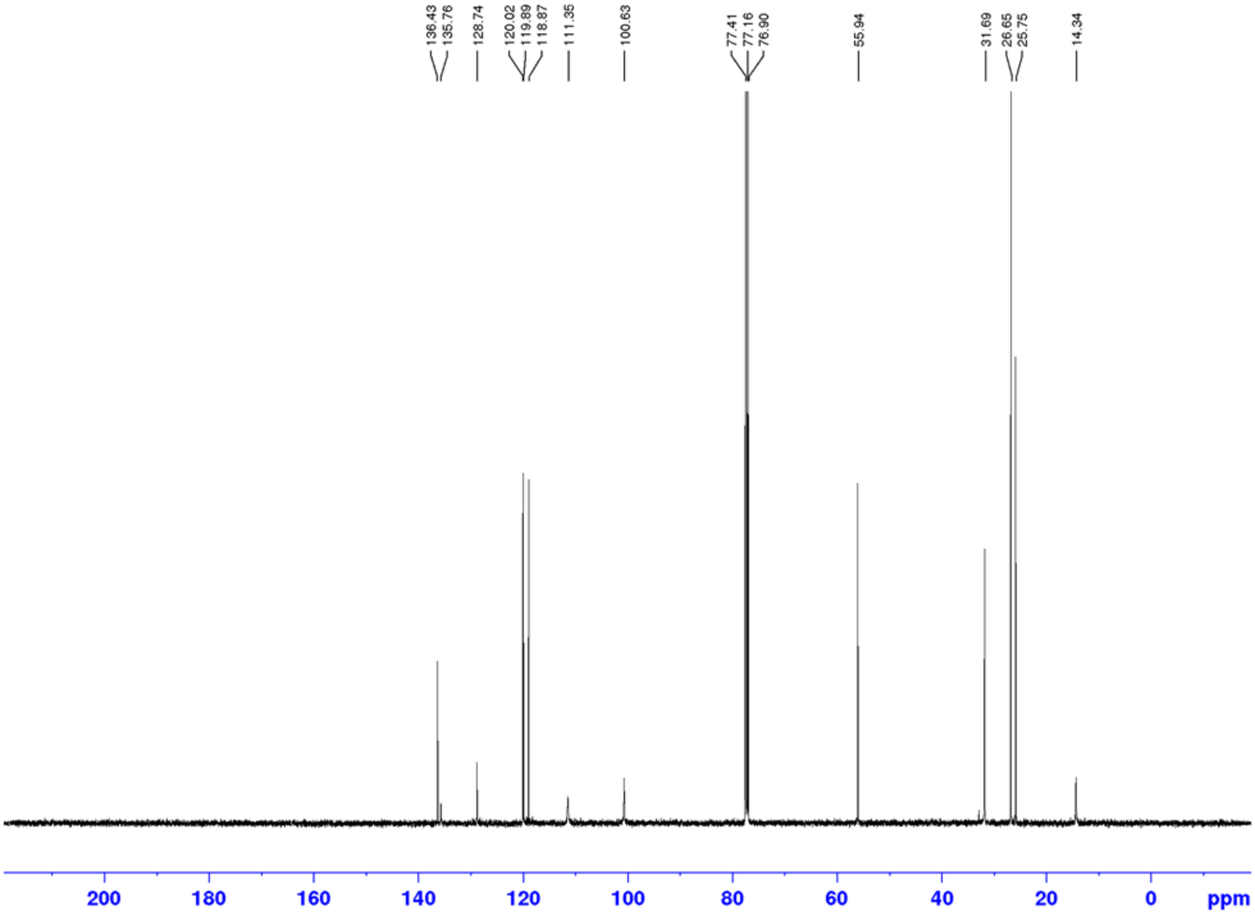
136.43
135.76
128.74
120.02
119.89
118.87
111.35
100.63
77.41
77.16
76.90
55.94
31.69
26.65
25.75
14.34
200
180
160
140
120
100
80
60
40
20
0
ppm

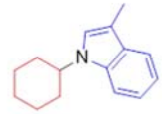

6d

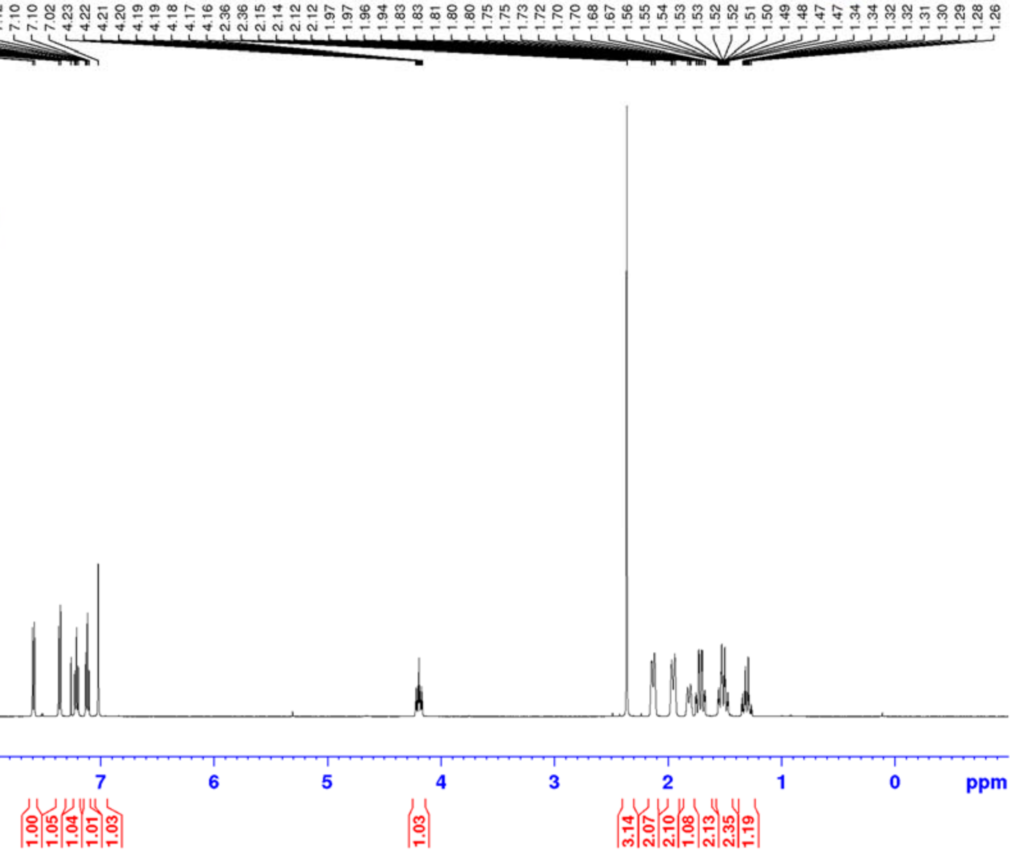
7.60
7.58
7.37
7.35
7.26
7.23
7.23
7.21
7.21
7.20
7.20
7.13
7.13
7.12
7.10
7.10
7.02
4.23
4.22
4.21
4.20
4.19
4.19
4.18
4.17
4.16
2.36
2.36
2.15
2.14
2.12
2.12
1.97
1.97
1.96
1.94
1.83
1.83
1.81
1.80
1.80
1.75
1.75
1.73
1.72
1.70
1.70
1.68
1.67
1.56
1.55
1.54
1.53
1.53
1.52
1.52
1.51
1.50
1.49
1.48
1.47
1.34
1.34
1.32
1.32
1.31
1.30
1.29
1.28
1.26
9
8
7
6
5
4
3
2
1
0
ppm
1.00
1.05
1.04
1.01
1.03
1.03
3.14
2.07
2.10
1.08
2.13
2.35
1.19

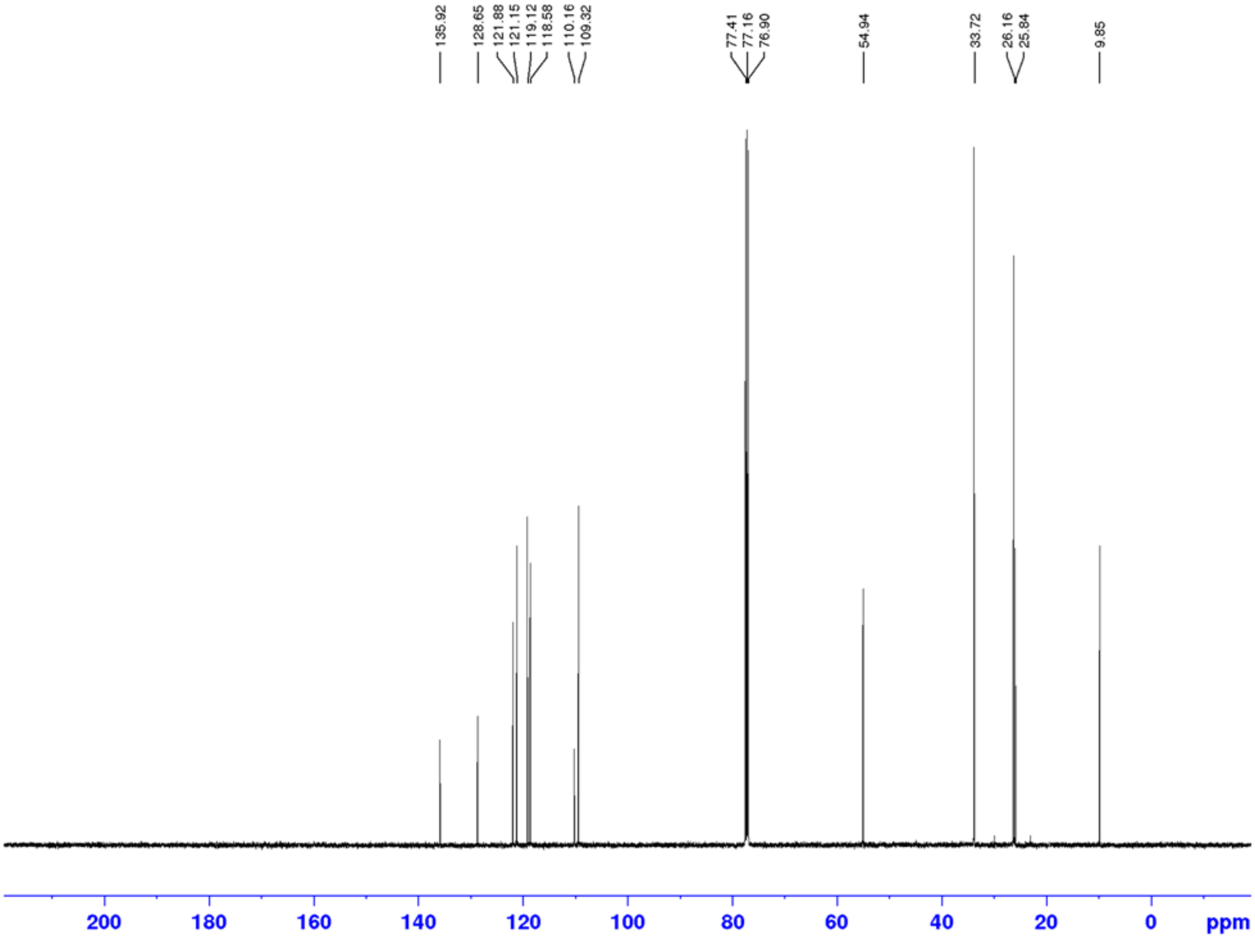
135.92
128.65
121.88
121.15
119.12
118.58
110.16
109.32
77.41
77.16
76.90
54.94
33.72
26.16
25.84
9.85
200
180
160
140
120
100
80
60
40
20
0
ppm

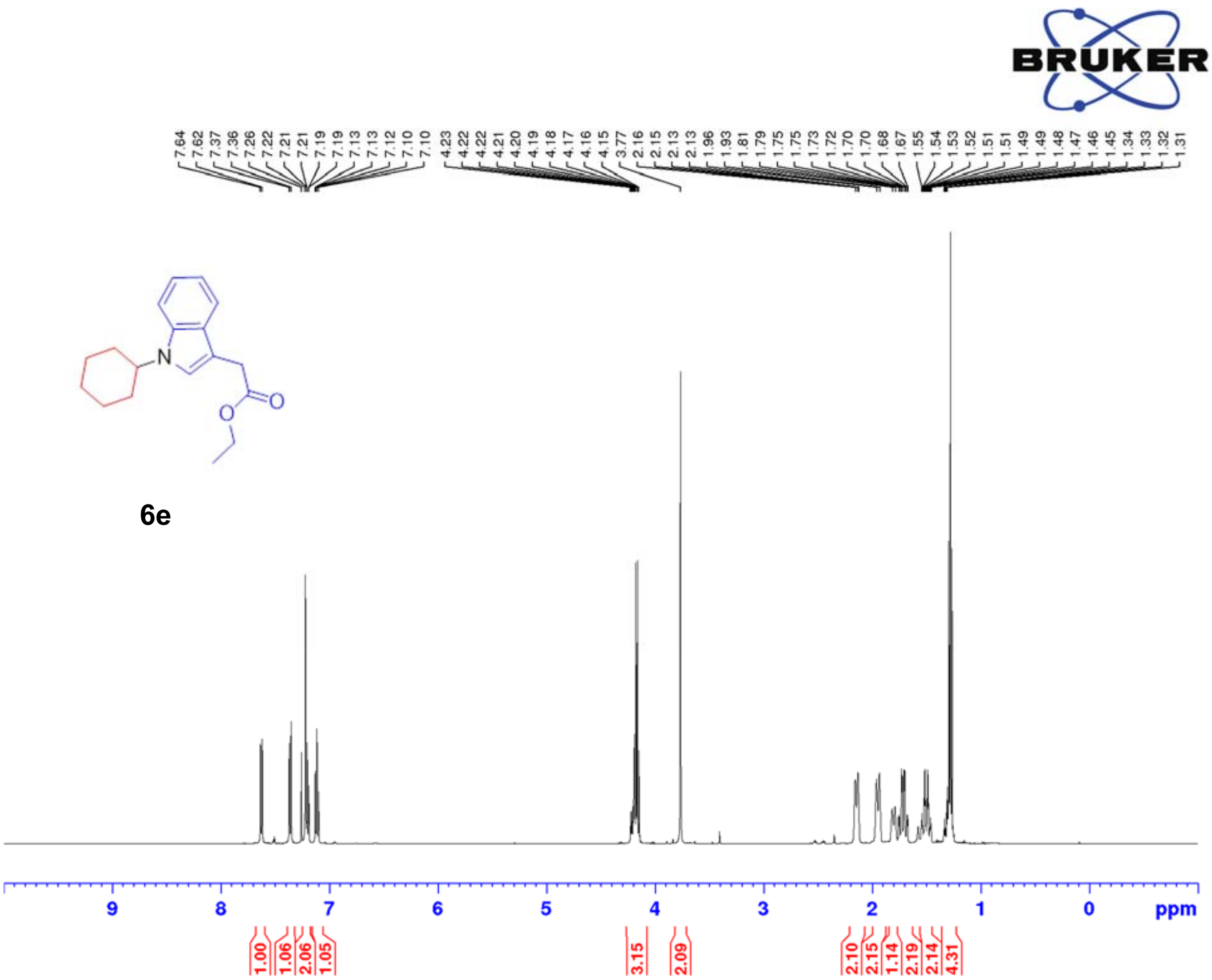
BRUKER
7.64 7.62 7.37 7.36 7.26 7.22 7.21 7.21 7.19 7.19 7.13 7.13 7.12 7.10 4.23 4.22 4.22 4.21 4.20 4.19 4.18 4.17 4.16 4.15 3.77 2.16 2.15 2.13 2.13 1.96 1.93 1.81 1.79 1.75 1.75 1.73 1.72 1.70 1.68 1.67 1.55 1.54 1.53 1.52 1.51 1.51 1.49 1.49 1.48 1.47 1.46 1.45 1.34 1.33 1.32 1.31
N
O
O
9 8 7 6 5 4 3 2 1 0 ppm
1.00 1.06 2.06 1.05 3.15 2.09 2.10 2.15 1.14 2.19 2.14 4.31

6e

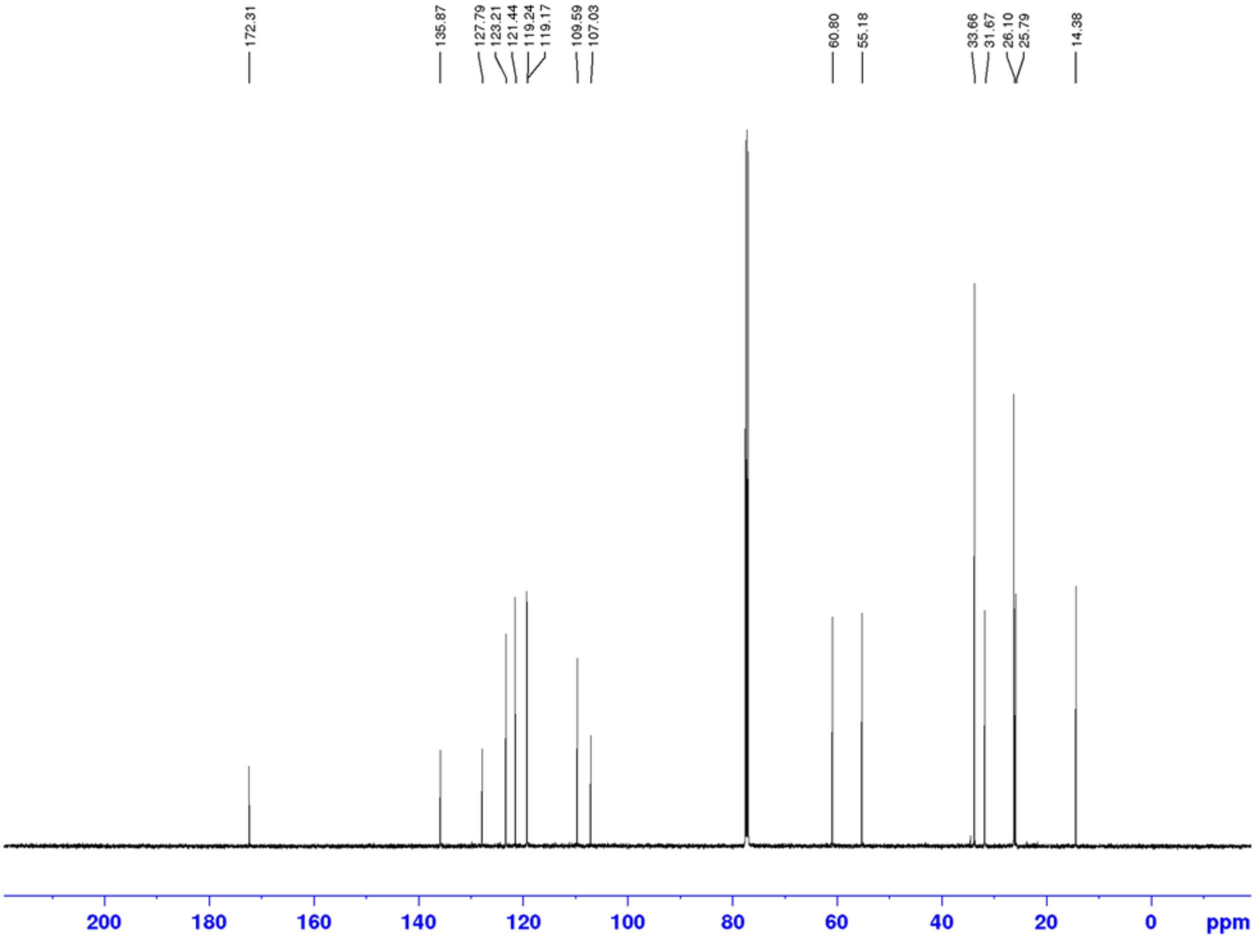
172.31
135.87
127.79
123.21
121.44
119.24
119.17
109.59
107.03
60.80
55.18
33.66
31.67
26.10
25.79
14.38
200
180
160
140
120
100
80
60
40
20
0
ppm

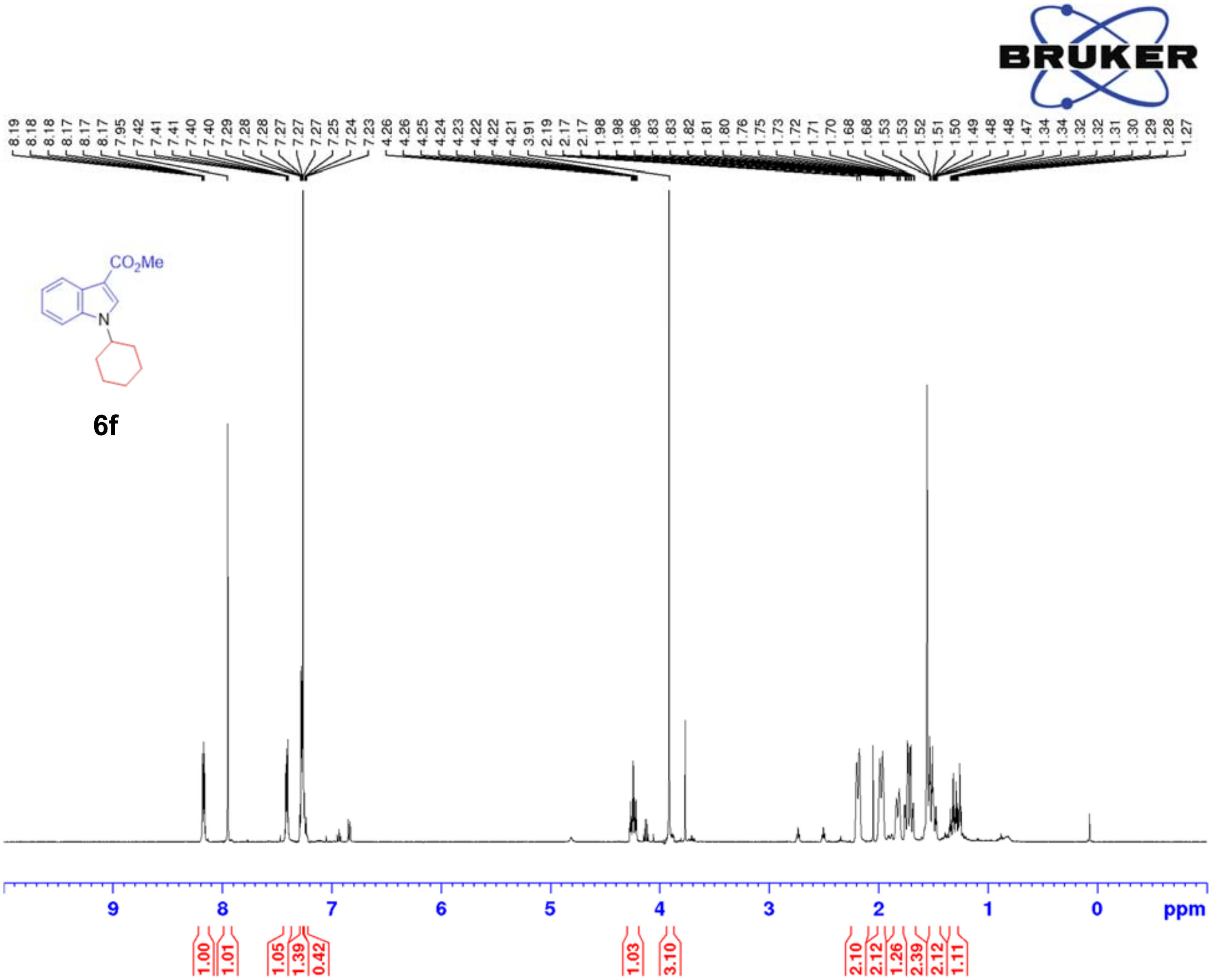
CO2Me
N
6f
ppm

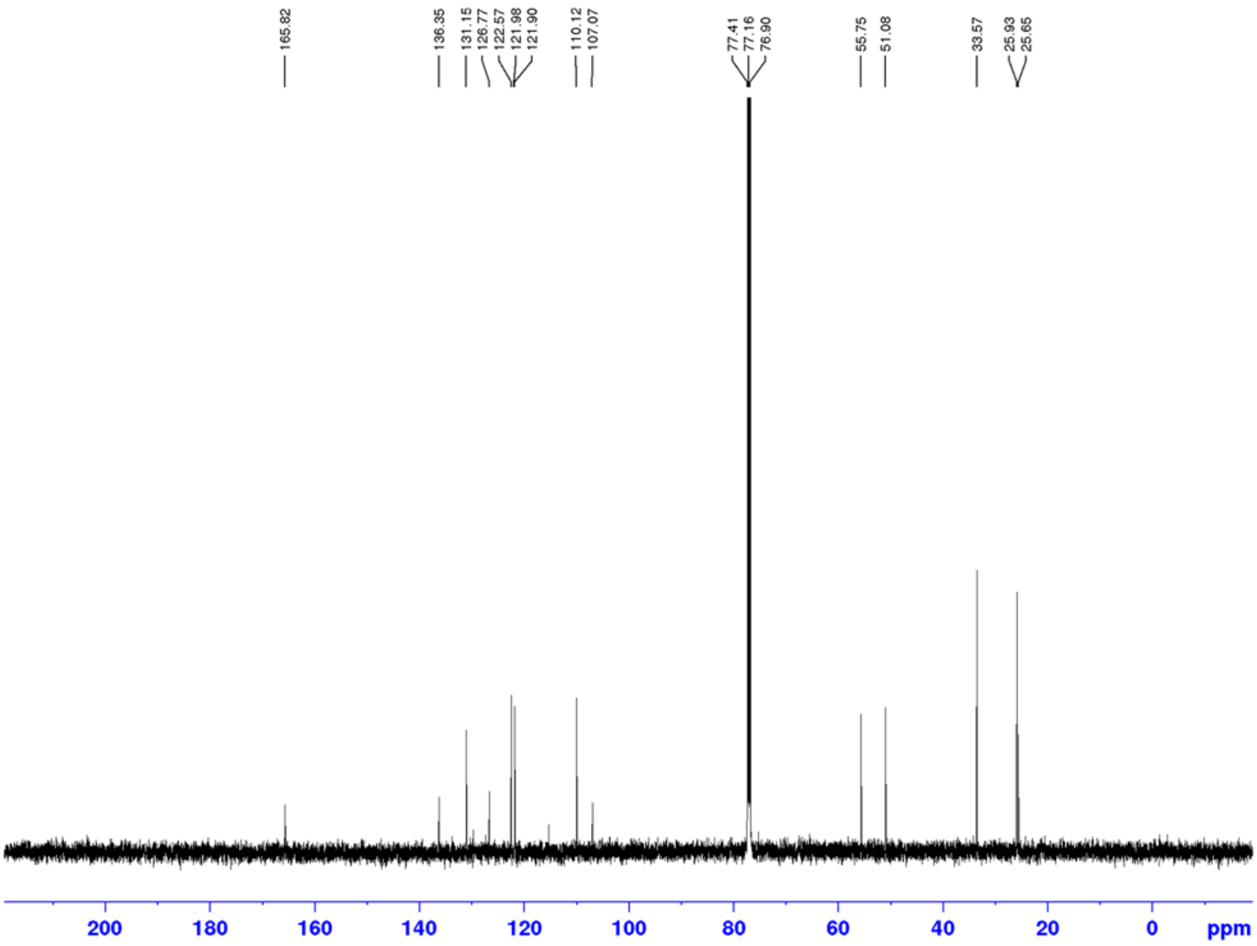
165.82
136.35
131.15
126.77
122.57
121.98
121.90
110.12
107.07
77.41
77.16
76.90
55.75
51.08
33.57
25.93
25.65
200
180
160
140
120
100
80
60
40
20
0
ppm

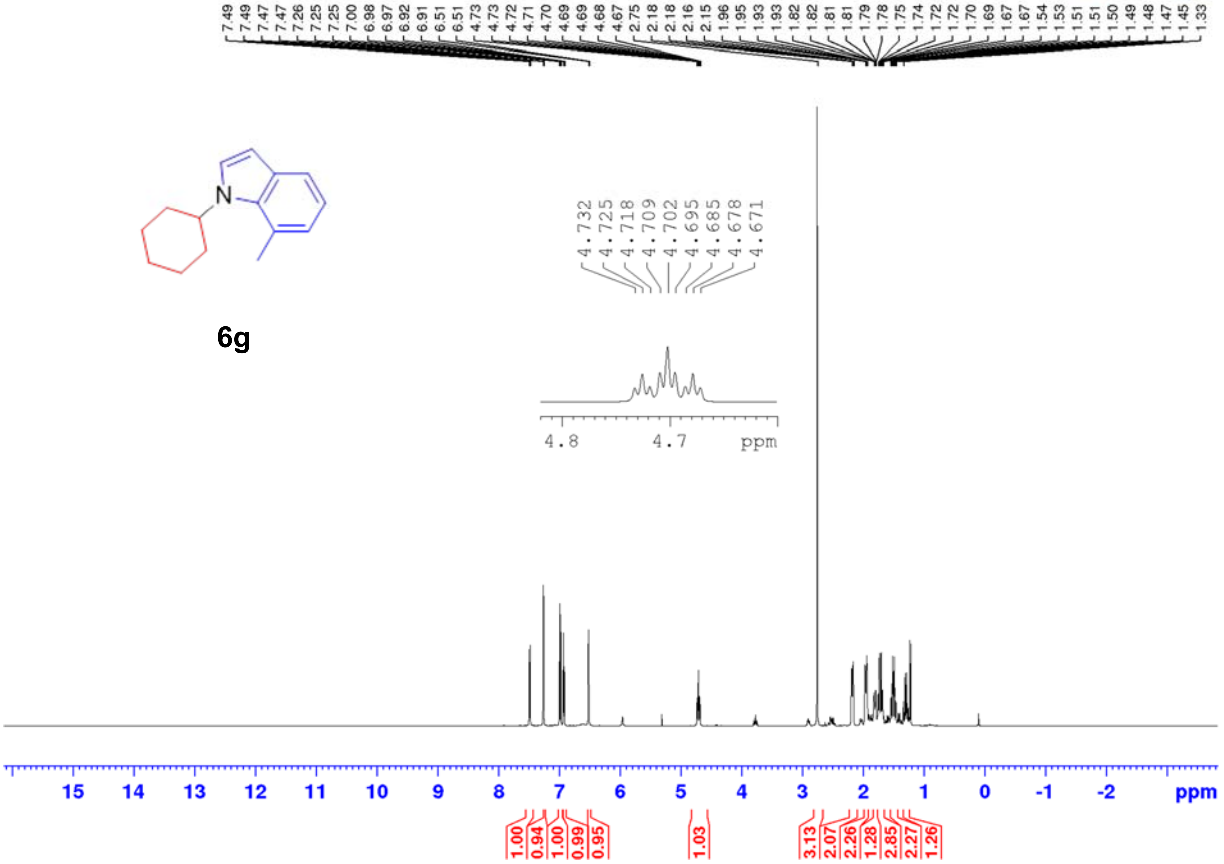

6g

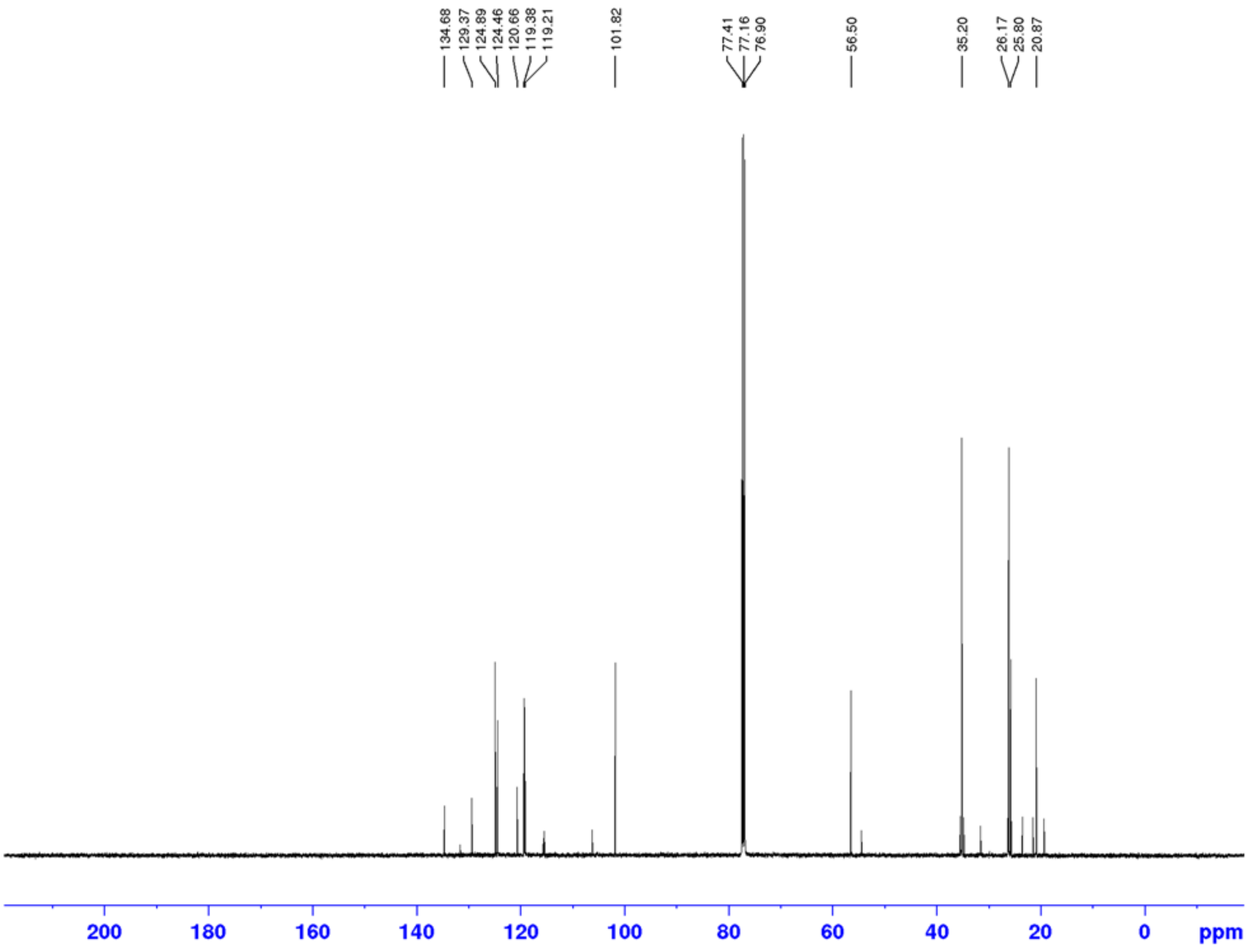
134.68
129.37
124.89
124.46
120.66
119.38
119.21
101.82
77.41
77.16
76.90
56.50
35.20
26.17
25.80
20.87
200
180
160
140
120
100
80
60
40
20
0
ppm

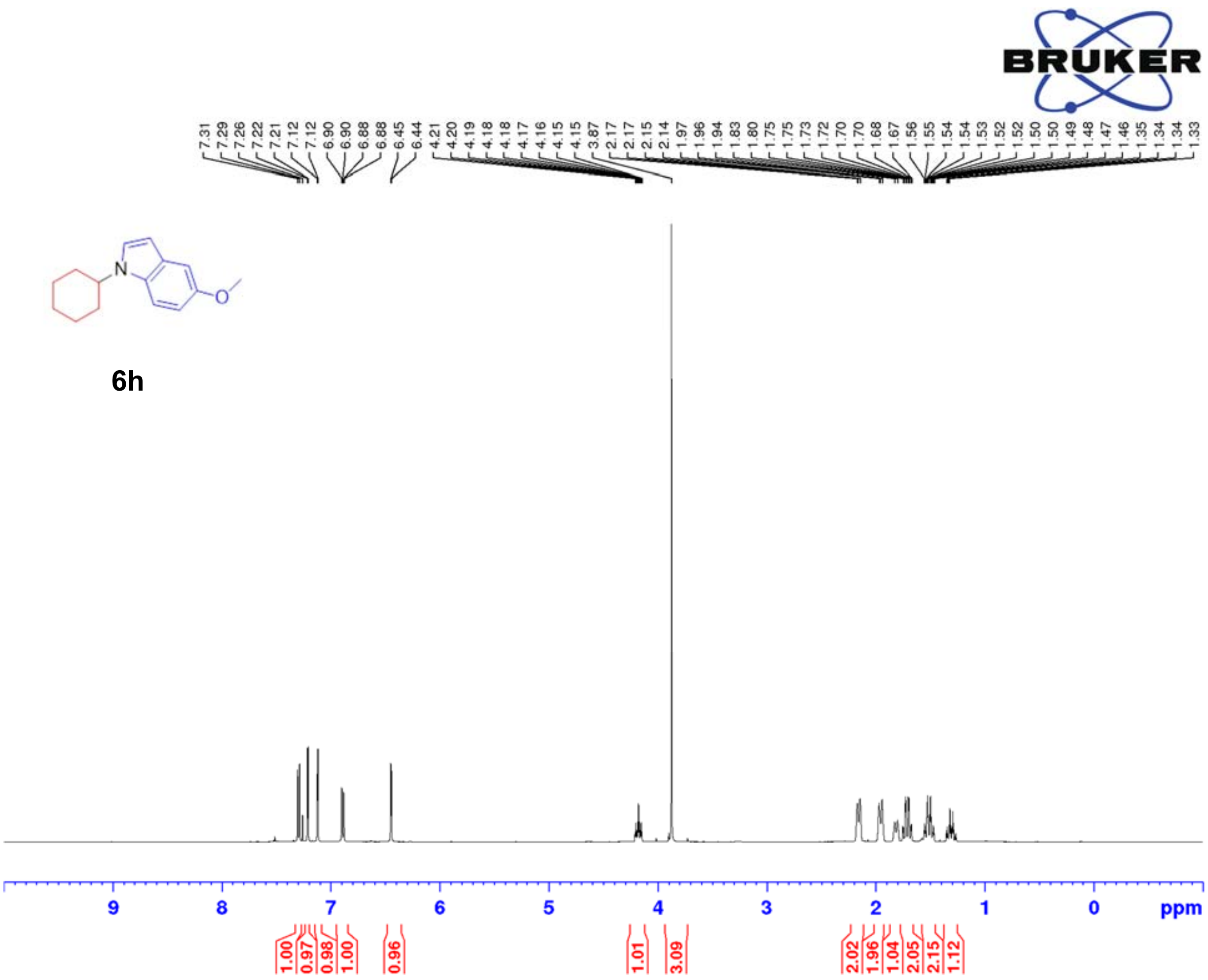

6h

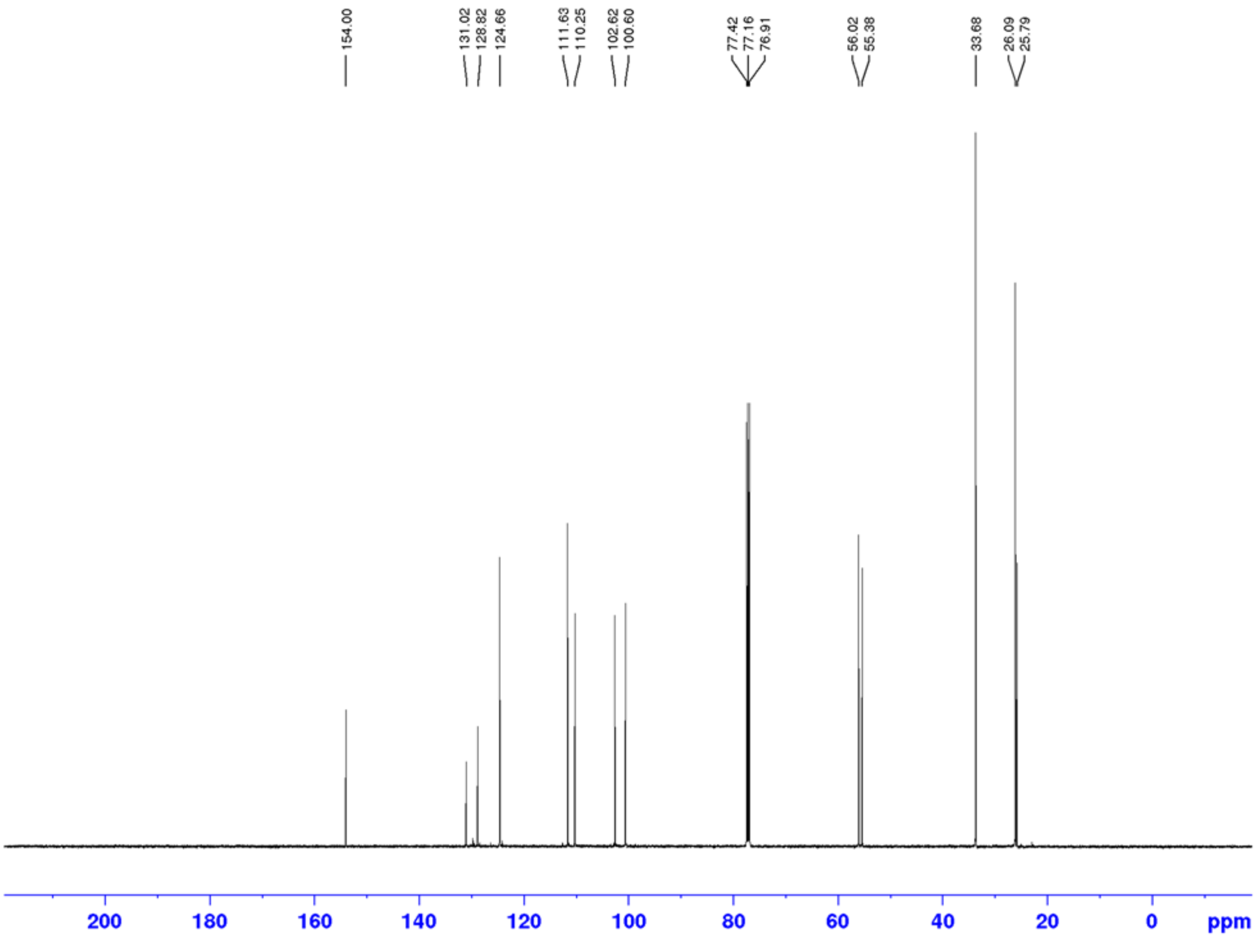

154.00
131.02
128.82
124.66
111.63
110.25
102.62
100.60
77.42
77.16
76.91
56.02
55.38
33.68
26.09
25.79
200
180
160
140
120
100
80
60
40
20
0
ppm

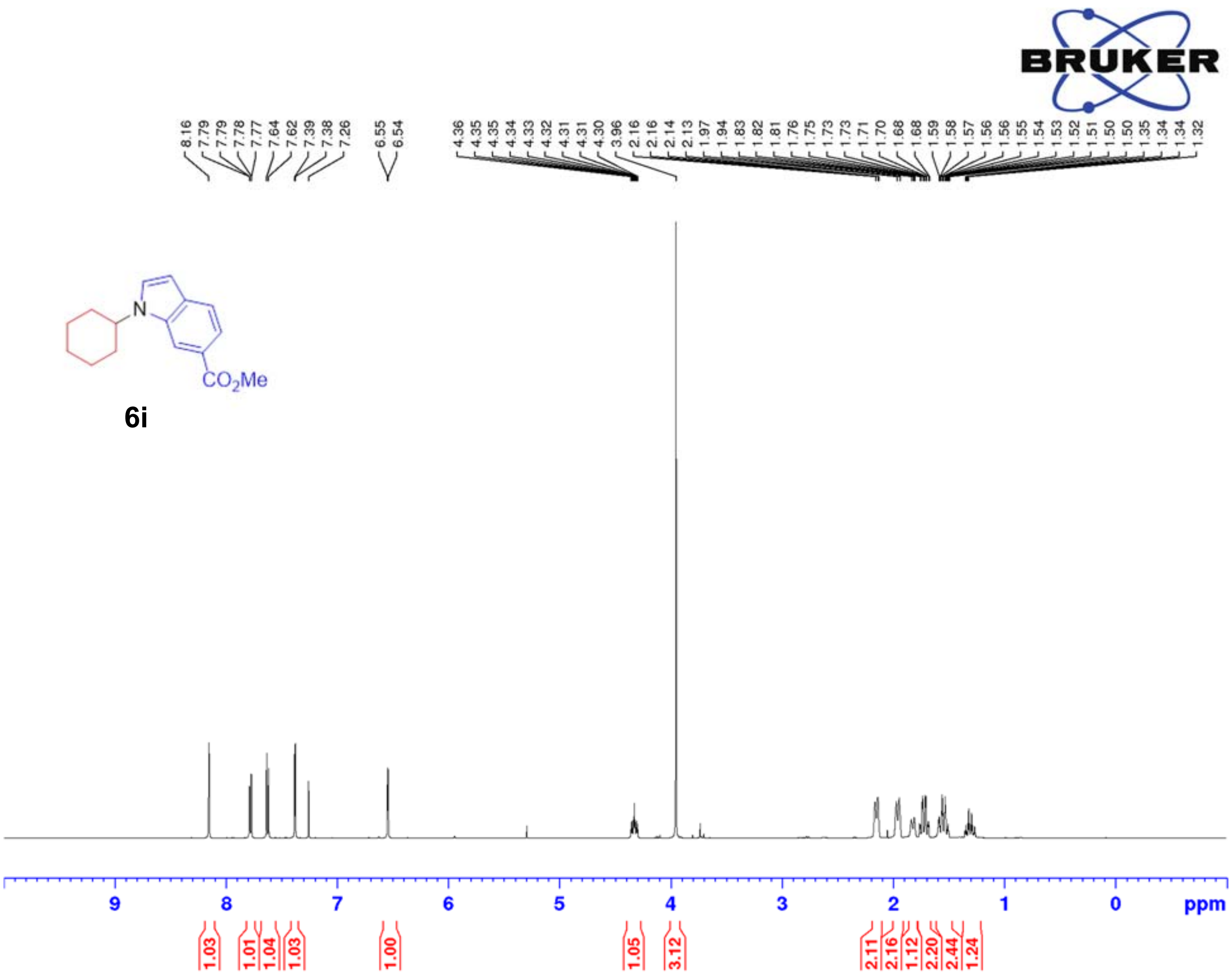

6i

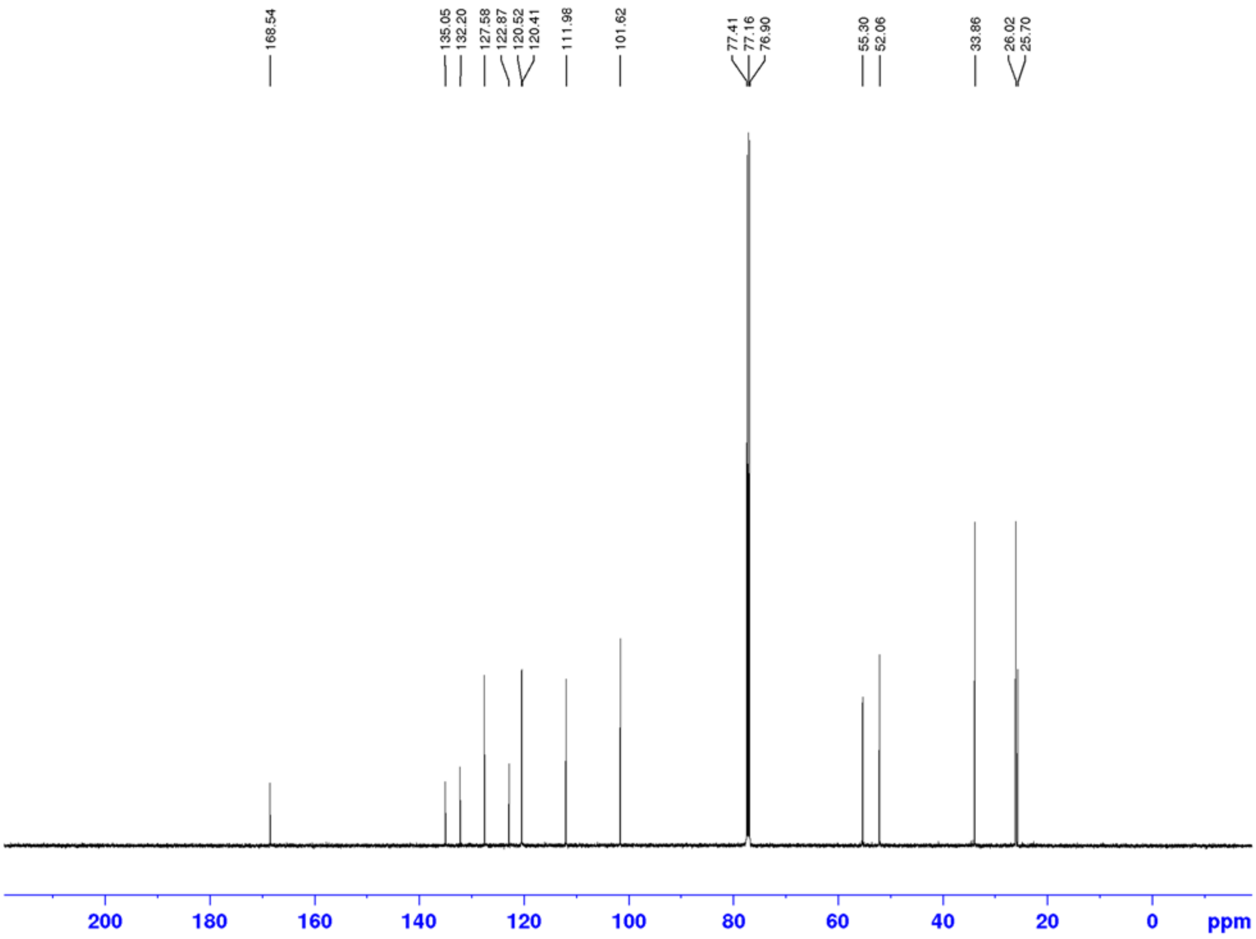
168.54
135.05
132.20
127.58
122.87
120.52
120.41
111.98
101.62
77.41
77.16
76.90
55.30
52.06
33.86
26.02
25.70
200
180
160
140
120
100
80
60
40
20
0
ppm
